# Recursive Consensus Clustering for novel subtype discovery from transcriptome data

**DOI:** 10.1038/s41598-020-67016-3

**Published:** 2020-07-03

**Authors:** Pranali Sonpatki, Nameeta Shah

**Affiliations:** Mazumdar Shaw Center for Translational Research, Mazumdar Shaw Medical Foundation, Narayana Hrudayalaya Health City, Bangalore, India

**Keywords:** Computational platforms and environments, Software

## Abstract

Large-scale transcriptomic data is used by biologists for the discovery of new molecular patterns or cell subpopulations. Clustering is one of the most popular methods for dimensionality reduction and data analysis for large scale datasets. The major problem while clustering the data is the selection of the optimal number of clusters (k) for each dataset and to discover new insights from it. We have developed Recursive Consensus Clustering (RCC), an unsupervised clustering algorithm for novel subtype discovery from both bulk and single-cell datasets. RCC is available as an R package and facilitates the generation of new biological insights through intuitive visualization of clustering results.

## Introduction

Recent advances in the field of RNA sequencing has resulted in a wealth of data which allows us to classify and study the transcriptomic subtypes/cell types in different biological systems. The clustering of transcriptomic data reduces the dimensionality of the data and allows a researcher to better analyze, visualize and interpret the data for biological insight. Clustering is an unsupervised technique that allows the grouping of similar objects and enables division of data. Though a widely used technique, researchers face the following challenges in performing clustering on transcriptomic data:

1. **Clustering of datasets with an unknown number of clusters:** Algorithmically identifying the optimal number of clusters in a dataset is a difficult mathematical problem especially for big datasets with a large number of clusters^1^. For example, algorithms like tSNE + k-means^2^ or hierarchical clustering require the user to input *k* for a given dataset which might not always be optimal. This also restricts the discovery of novel subtypes in a given dataset.
2. **Finding novel subtypes with user-friendly tools:** Subtypes that are not known beforehand are likely to be missed when applying popular clustering methods like k-means, hierarchical clustering, pam, mclust^3^, etc.^4^ As an example, consider large-scale TCGA pan-cancer dataset^5^ which includes samples from multiple cancer types (breast, prostate, brain, etc.) with each cancer type having distinct molecular subtypes. Two major publications that analyzed this transcriptome data were able to largely find clusters with tissue-specific cancers and not the subtypes within each cancer type with clinical relevance^5,6^. The novel subtypes were discovered only after using sophisticated integrated analysis of multi-omic data.

We have developed Recursive Consensus Clustering (RCC), a user-friendly R package that allows a researcher to find novel and biologically meaningful subtypes in transcriptomic datasets without requiring computational expertise (https://github.com/MSCTR/RecursiveConsensusClustering). The recursive clustering of the dataset reveals finer structures in the data leading to the identification of novel subtypes. RCC uses ConsensusClusterPlus^7^ (CCP) as the base algorithm for dividing the data. Most of the clustering algorithms are heuristic in nature and the clusters obtained using them depend on the initial seed value^8^. As a result, different runs of the same algorithm on the same dataset yield different clusterings. To deal with this issue the concept of consensus clustering was developed where you run the algorithm multiple times and take the consensus of clusters from those runs as your final result. CCP, an R package for consensus clustering uses *n* iterations of any one of the clustering algorithms (e.g. k-means, hierarchical clustering, partitioning around medoids, etc.) to divide the data into various subgroups. For each of the *n* iterations, CCP repetitively takes a subset of samples/cells and a subset of genes/features to give a consensus of these repetitions, which results in a more robust clustering output relative to the clusters obtained through a single iteration.

## Results

### Algorithm

**Figure 1:**
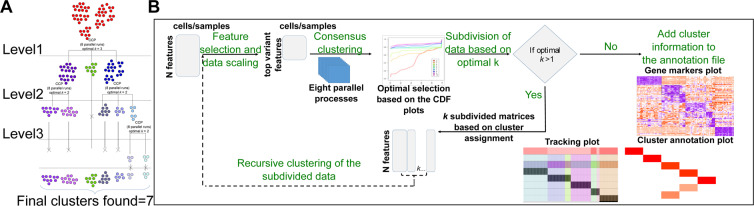
RCC workflow. (**A**) Recursive nature of RCC. As shown in the figure RCC recursively divides the datasets till the termination criteria are met. Due to the recursive nature of RCC, it produces multi-level cluster assignments, which are then concatenated to output the final number of clusters. RCC determines the optimal k for each run individually. As seen in the figure, the dataset is divided into seven clusters across three level of recursive clustering. (**B**) Overview of RCC workflow.

RCC takes the entire data matrix in the first run and finds the optimal *k* for the data as a whole. The output from this first run is termed as level one. Based on the clustering in level one, RCC then subdivides the data into respective clusters and recursively clusters each subdivision. This subsequent clustering gives us multi-level subgroupings of the data. In the end, RCC combines the clustering information at each level to return the final number of clusters present in a given dataset (Fig. 1A).

The RCC algorithm is described in five steps as shown in Fig. 1B.

1. Data input: RCC takes a quantitative transcriptomic data matrix as input along with a configuration file (Methods). Annotation matrix file for samples/cells can be provided optionally.
2. Feature selection and data scaling: For every recursive run, RCC selects the top n% of variant genes in that particular subset of the original dataset which is used for further clustering. The top n% of genes/transcripts change for each recursive run. This helps in finding the finer hierarchical structure in a given dataset, e.g. in human tissue data^9^, the brain tissue samples are further divided into 13 subtypes which include different sections of brain like cortex, basal ganglia, cerebellum, amygdala, cerebellar hemisphere, substantia nigra, hippocampus, hypothalamus and spinal cord. When we take genes which are variable across all the tissue types, the clustering algorithms are not able to divide the data at these sub-tissue levels (Figure S1B). Hence it is essential to change the feature set for clustering based on each run (Figure S1).
3. Parallel processing of ConsensusClusterPlus: RCC uses the ConsensusClusterPlus package^7^ (CCP) in R for k-means clustering of the datasets. CCP uses three input parameters; seed, pItem (proportion of samples), and pFeature (proportion of genes/transcripts), that determines the final clustering outcome. Depending on the type of dataset, a user may need to change the pItem and pFeature. To reduce user input and increase robustness, we use eight combinations of pItem/pFeature (Table S1) and run CCP eight times in parallel with 100 repeats(with different random seeds) each. In order to compare our strategy to simply running CCP 800 times we ran the RCC algorithm in two different settings; a) RCC algorithm with eight parallel processes with changes in pItem and pFeature values and 100 repetitions per process, b) RCC algorithm with no parallel processes, default pItem and pFeature values and 800 repetitions. We observed that RCC with parallel processes and variant pItem and pFeature values showed higher ARI compared to the other process (Table S2). Running eight parallel processes with 100 repetitions each was also faster than running one process with 800 repetitions (Table S2).
4. Optimal *k* selection: Based on the cumulative distribution function (CDF) plots indicative of cluster stability, produced by CCP, RCC finds the best *k*s^10^ for each parallel run, in turn, giving multiple *k* values that satisfy the best *k* selection criteria (Methods). The *k* with maximum frequency is selected as the optimal *k* for the clustering (Figure S2).
5. Subdivision of data and recursive clustering: Once the *k* for a given dataset is selected, all *k* subsets are clustered recursively till one of the termination criterion is met i.e.

1. optimal *k* = 0; i.e. the data does not show any significant variance in it or there are not enough differential genes present to indicate a true biological sub-classification.
2. the sample size of the subset data is lower than the specified minimum number of samples required for clustering

6. Output: RCC outputs final cluster information as well as cluster information at all levels in a csv format. It also outputs the clustered data in the form of tracking plot and cluster annotation plot for visualization of results facilitating biological interpretation (Results, Methods).

We have used k-means as the underlying clustering algorithm for RCC as we found it works well for bulk transcriptomic datasets of different sizes. In principle, any clustering algorithm can be substituted for k-means. As the result obtained from the k-means algorithm is highly dependent on initial seed value^11^, we run CCP eight times using a different random seed and different parameters for each run to get the most stable clusters (Methods).

**Figure 2:**
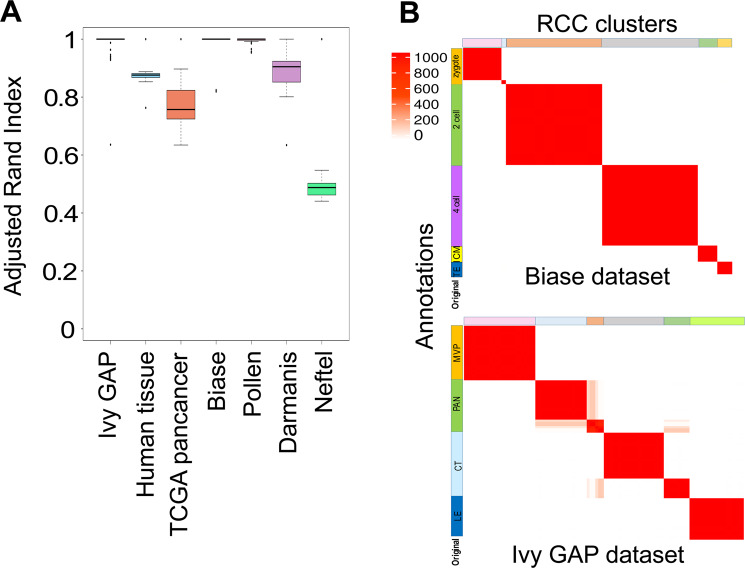
Robustness of RCC. (**A**) Adjusted Random Index (ARI) of multiple runs (1000 runs for Ivy GAP, Biase and Pollen datasets, 100 runs for Darmanis dataset and 10 runs for Human tissue, TCGA pan-cancer and Neftel datasets) where ARI is calculated for each run with that of one randomly selected run (**B**) Consensus matrix of 1000 RCC runs for Biase and Ivy GAP datasets showing the robustness of the algorithm.

To minimize user dependency and find appropriate values for all the parameters we tested this algorithm using various bulk and single-cell datasets (Table 1). All the selected datasets were annotated and their labels were obtained from original publications. RCC worked well on bulk as well as single-cell datasets. For most of the datasets, RCC was able to find biologically significant novel subtypes (Table 1). To check the stability of RCC, we ran the algorithm 1000 times on Ivy GAP^12^, Biase^13^, and Pollen^14^ datasets, 100 times on the Darmanis^15,16^ dataset and 10 times on Human tissue^9^, TCGA pan-cancer^5^, and Neftel^17^ datasets. We calculated the Adjusted Random Index (ARI) using the mclust^3^ package in R of each run with that of one randomly selected run. ARI calculates the concordance between two cluster assignments. The cluster assignment can be based on the annotation/labels from the annotated datasets or the clusters obtained through RCC and other algorithms. Higher ARI indicates better concordance between two cluster assignments with value 1 being the perfect concordance between two cluster assignments. We see highly consistent clustering results between RCC runs with ARI ranging from 0.5 to 1 (Fig. 2A). The consistency of the results for Biase and Ivy GAP datasets can be seen in the consensus matrices (Fig. 2B). The consensus matrices for the rest of the datasets are provided in Supplementary data in the individual folders. One of the features of RCC is the automatic selection of the number of clusters for a given dataset. So to benchmark RCC, we chose mclust^3^ (for bulk dataset) and SC3^18^ (for single-cell dataset) packages which also have the feature of selecting the optimal *k*. We have also compared our algorithm against tSNE + k-means^2^ and hierarchical clustering (hclust, base R package) by giving it the *k* based on known annotation from the original publications (Table S3). We were not able to run mclust on larger datasets (i.e. datasets having samples > 1000) on our system (system specifications in Methods). Overall, we found good concordance between the clusters found by RCC and labels suggested by the original authors (Tables S4, S5). In all but one case, RCC found novel subtypes, i.e. further subdivision of known attributes at the time of data generation as provided in the original study. (Table 1, Results).

**Table 1:** Datasets used to test RCC algorithm where N represents the number of cells/samples in a dataset, k represents the number of clusters identified by the authors in the original publications, fourth column states if RCC found biologically relevant novel subtypes in the respective dataset, RCC, mclust and SC3 columns state the number of clusters found by the respective algorithms.

Datasets	*k*	N	Subtype found By RCC	Clusters found by		
				RCC	mclust	SC3
Ivy GAP	4	98	Yes	6	6	NA
Human tissue	53	8739	Yes	144	NA	NA
TCGA pan-cancer	32	10042	Yes	124	NA	NA
Biase	5	56	No	6	NA	5
Pollen	11	300	Yes	13	NA	10
Darmanis	19	4055	Yes	43	NA	25
Neftel	31	7930	Yes	80	NA	61

We used the datasets mentioned in Table 1 for testing the algorithm. All the bulk datasets were normalized using the DESeq^19^ package in R and single-cell data using Counts Per Million (CPM) and then log2 transformed before running RCC. To benchmark RCC, we used the algorithms given in (Table S3) on the bulk as well as single-cell transcriptomic datasets. Due to the sparsity of single-cell data matrices, we included only the protein-coding genes for the clustering proposes. We used the default parameters for all algorithms and provided the number of clusters where needed. For the algorithms that did not have the optimal *k* selection feature, we provided the same number of clusters presented in the original publications. The optimal *k* and Adjusted Random Index (ARI) for each dataset across all the algorithms is shown in Table S4. Identification of novel subtypes results in lower ARI values. Since RCC results have a significant number of subtypes for larger datasets we see lower ARI values. To show that the novel subtypes are subsets of known attributes, we calculate cluster specificity score for each clustering result as the percentage of clusters that are composed of largely samples having the same attribute, i.e. dominant attribute type proportion > = 90%. RCC did a better job of finding novel subtypes as well as dividing the datasets accurately based on the given annotations in the original publications (Table S5).

Along with clustering, RCC package also provides the user with visualization tools for better interpretation. In case of large-scale datasets, it becomes difficult to view the cluster assignments along with the attributes of each sample/cell in the data. To make it easier to view the clustered data, RCC package provides the users with tracking and cluster annotation plots. The tracking plot makes it easy for the user to view the clustered data and interpret it compared to other visualization tools (Figure S3). Columns in the tracking plot correspond to the samples/cells in the provided dataset. The first panel *i* is the marker panel which shows the number of marker genes identified for each cluster at a given level. The first row is markers for clusters found at level one, the second one for markers found at level two, and so on. The color-scale is from white to red with white indicating zero markers found and bright red indicating the highest number of markers found. Grey color indicates that the particular cluster is not further divided and hence no new markers are found which are distinct from the previous level. The second-panel *j* is the RCC clustering level panel. It shows the cluster information for samples at each level starting with level one. The third panel *k* is the annotation panel. The annotation panel shows the distribution of samples across all the clusters. Due to the two-dimensional nature of the plot, it becomes easier to view the cluster assignments along with its annotation. The cluster annotation plot allows the user to view the cluster assignment of each sample based on a particular attribute. It shows the proportions of samples based on attributes across all the clusters allowing the user to view the most dominant attribute or label in a given cluster.

The main objective of developing RCC was to find novel subtypes automatically with minimal user input that can help in generating novel biological insights. We demonstrate this utility of RCC by applying it to seven different datasets of varied sizes and different complexities (Table 1).

### Bulk RNA-Seq data

1. **Ivy GAP dataset:** The Ivy GAP^12^ dataset contains the RNA-Seq profiles of anatomic structures in glioblastoma, grade IV brain cancer. These anatomic structures include Leading Edge (LE), Cellular Tumor (CT), Microvascular Proliferation (MVP), and Pseudopalisading Cells around Necrosis (PAN) which are described in detail in the original paper. RCC clusters not only represent all the known classes but also find two subsets of CT and PAN samples each (Fig. 3A). These subtypes of CT and PAN samples were not found by any other algorithm (Fig. 3B). The gene markers plot shows that the subtypes of CT and PAN are distinguished by a distinct proneural signature^20^ (Fig. 3C).

**Figure 3:**
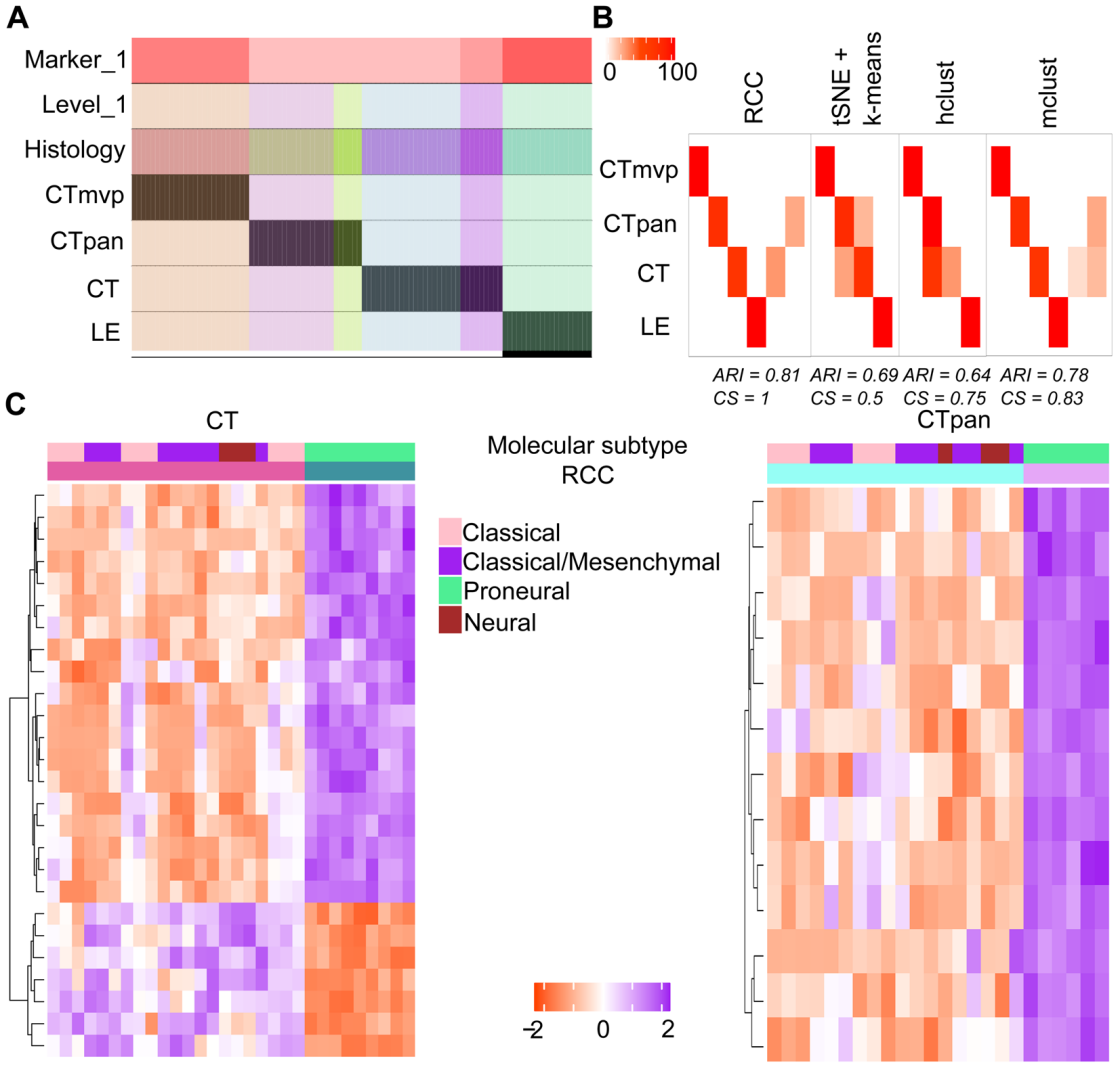
Ivy GAP data. (**A**) Tracking plot for the Ivy GAP dataset showing the multi-level clustering information. (**B**) Cluster annotation plots for RCC, t-SNE + k-means, hclust, and mclust algorithms. Each plot shows the overlap between the annotations and clusters found. Along the x-axis are the clusters found by each algorithm and along the y-axis are the anatomical features of Ivy GAP data. Red indicates a 100% overlap between the annotated class and the cluster and white indicates no overlap. (**C**) Gene marker plots for Ivy GAP data. In RCC clustering, CT and PAN samples subdivide into two clusters each. The rows represent genes and columns represent samples. The annotation bar shows the demarcation between two clusters. RCC finds a subset of CT and PAN samples that have a proneural subtype.
2. **Human tissue data:** Human tissue data^9^ contains RNA-Seq data across 53 different tissues, lymphoblastoid cell lines, and transformed fibroblast cell lines. This dataset is challenging to cluster as certain tissue types like tissues from the female reproductive system, adipose and breast tissues, tissues from different parts of the brain, etc. are difficult to distinguish when global transcriptome similarity is considered using their grade of membership model. RCC is more sensitive in comparison to other algorithms in clustering these samples due to its recursive approach (Fig. 4A,B, and S4, Table S5, Supplementary file 2). RCC could find multiple biologically relevant subtypes across 13 tissues which other algorithms were not able to find (Supplementary file 2). As an example, lung tissue for which RCC found eight clusters, tSNE + k-means and hclust could find only two and one clusters respectively (Fig. 4B). On further analysis of these eight clusters, we found that cluster one shows enrichment of myeloid leukocyte activation and cell activation gene sets and patients with decreased pulmonary function, cluster two has gene sets with extracellular matrix component and subjects who suffered fast deaths, cluster three shows enrichment of myeloid leukocyte migration and cell migration gene sets, cluster four shows genes involved in immunoglobulin receptor binding, protein activation cascade, cluster five shows up-regulation of genes involved in positive regulation of protein secretion, plasminogen activation, cluster six shows up-regulation of genes involved in cytokine activation, regulation of vasculature development, cluster seven shows up-regulation of genes involved in pulmonary embolism and complement activation and classical pathway and cluster eight has up-regulation of genes which have cilium cellular component (Fig. 4C, Table S6). We correlated the significance of clinical parameters like age, gender, and cause of death with the clusters formed. Out of 53 tissue subtypes, we found seven tissue subtype clusters that showed significant correlation with gender, nine showed significant correlation with age, and 26 showed significant correlation with the cause of death across 10 runs of RCC (Supplementary file 1). Figure 4D shows the distribution of age, cause of death, and gender across the muscle, heart and breast samples respectively.

**Figure 4:**
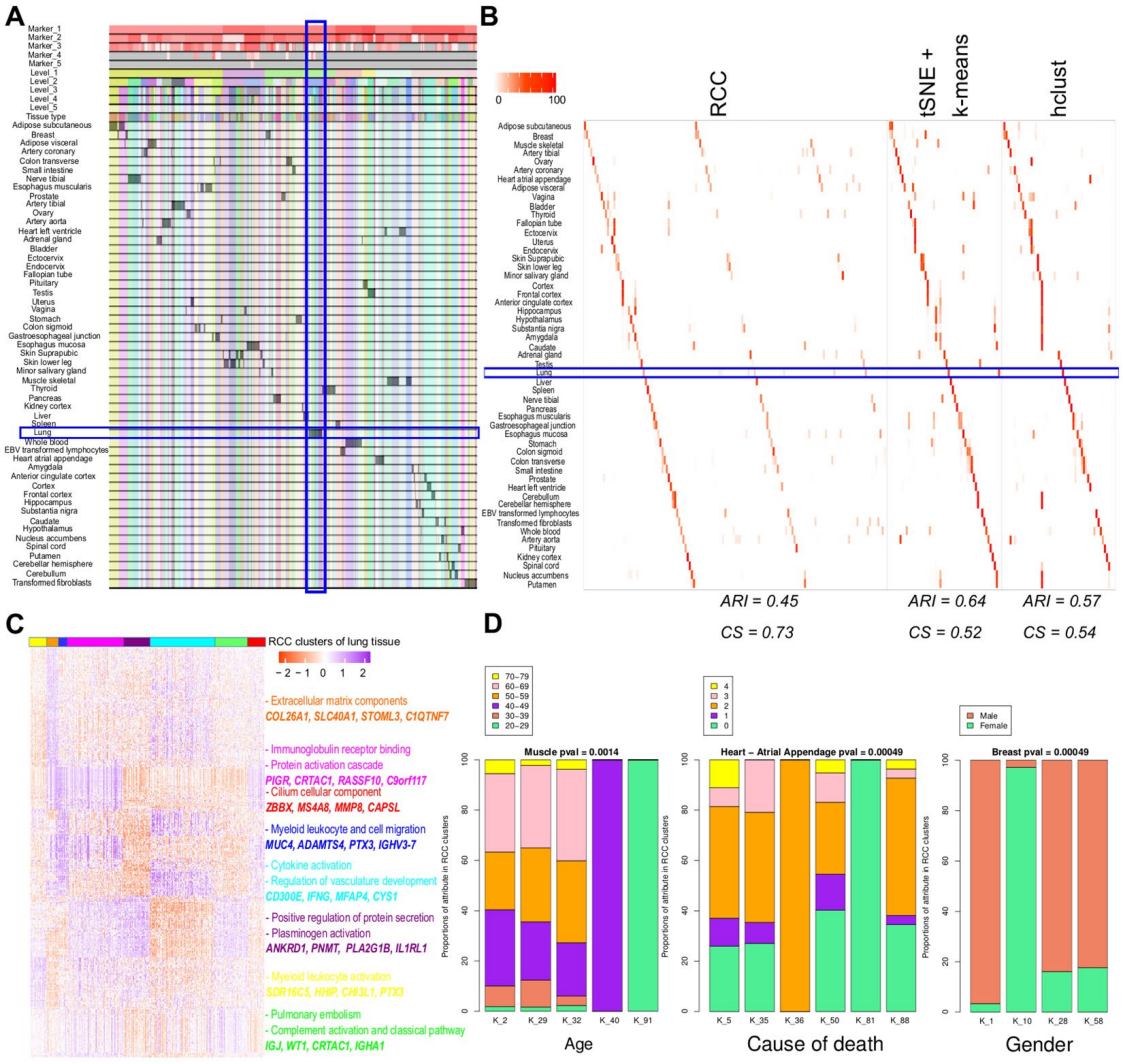
Human tissue data. (**A**) Tracking plot for human tissue data showing the multi-level clustering information. The lung tissue samples are highlighted with a blue box. (**B**) Cluster annotation plots for RCC, t-SNE + k-means, and hclust algorithms. Each plot shows the overlap between the annotations and clusters found. Along the x-axis is the clusters found by each algorithm and along the y-axis are the tissue annotations. Red indicates a 100% overlap between the annotated class and the cluster and white indicates no overlap. The lung tissue clusters are highlighted in the blue box. RCC divides the lung tissue samples into eight clusters, whereas tSNE + k-means found two clusters and hclust found none. (**C**) Gene marker plot for Lung samples from human tissue dataset (gene marker list available in Table S6). RCC divides the lung samples into eight clusters where cluster one shows enrichment of myeloid leukocyte activation and cell activation gene sets and patients with decreased pulmonary function, cluster two has gene sets with extracellular matrix component and subjects who suffered fast deaths, cluster three shows enrichment of myeloid leukocyte migration and cell migration gene sets, cluster four shows genes involved in immunoglobulin receptor binding and protein activation cascade, cluster five shows genes involved in positive regulation of protein secretion and plasminogen activation, cluster six shows up-regulation of genes involved in cytokine activation and regulation of vasculature development, cluster seven shows up-regulation of genes involved in Pulmonary embolism and complement activation and classical pathway and cluster eight has up-regulation of genes that have a cilium cellular component. (**D**) Attribute enrichment plots with RCC cluster assignments. RCC can find clusters with distinct clinical attributes. In muscle tissue data, RCC found a subset of samples with distinct gene markers and between in the age group of 40–49and 20–29. Similarly, in heart atrial appendage tissue type RCC was able to separate a set of samples where the subject was on a ventilator as well as subjects with sudden unexpected deaths. In the case of breast tissue samples, RCC can divide the data based on gender along with tissue subtype. The COD follows a Death classification based on the 4-point Hardy Scale: (1) Violent and fast death Deaths due to accident, blunt force trauma or suicide, terminal phase estimated at <10 min. (2) Fast death of natural causes Sudden unexpected deaths of people who had been reasonably healthy, after a terminal phase estimated at <1 hr (with sudden death from a myocardial infarction as a model cause of death for this category). (3) Intermediate death Death after a terminal phase of 1 to 24 hrs (not classifiable as 2 or 4); patients who were ill but death was unexpected. (4) Slow death Death after a long illness, with a terminal phase longer than 1 day (commonly cancer or chronic pulmonary disease); deaths that are not unexpected. (0) Ventilator Case - All cases on a ventilator immediately before death.
3. **TCGA pan-cancer data:** TCGA pan-cancer^5^ dataset includes RNA-Seq data of 32 different cancer types. RCC divided the data up to four levels and was able to find 124 clusters in this dataset resulting in novel cancer subtypes (Fig. 5A,B). As seen in Fig. 5A, RCC divides the data into 10 clusters at level one, which are more tissue specific. In further levels of RCC, it divides the samples into its individual cancer types and then further finds the subtypes within each cancer type. As an example, we show subtypes of melanoma (SKCM) in Fig. 5C. RCC divides the SKCM samples at level one in two distinct clusters. Then further at level four, RCC is able to find three more subtypes of the SKCM samples. In total RCC found four subtypes in the SKCM cancer samples highlighted in Fig. 5A, with significantly distinct marker genes and survival profiles (Fig. 5C, Table S7). Similar molecular profiles were previously found and discussed^21^. SKCM subtypes were not found by tSNE + k-means, whereas, hclust found four subtypes of SKCM but showed survival profile overlap between two clusters (Supplementary data, Table S8). Chen *et al*.^6^ and Hoadley *et al*.^5^ previously analyzed the TCGA pan-cancer data using various sophisticated techniques. They found ten and 28 clusters respectively to divide data based on the multi-omic analysis using the cluster of clusters approach. We performed Kaplan-Meier analysis for RCC, tSNE + k-means, hclust cluster assignments along with cluster assignments by Chen *et al*. and Hoadley *et al*. to evaluate which of the clustering assignments are clinically relevant. We used patient survival data as a measure of clinical relevance. In our comparison, we found that using RCC assignments, there are 10 cancers with subtypes which show significantly distinct survival profiles with p-value <0.01 across 10 runs, for tSNE + k-means clusters there are three, five for hclust, three for Hoadley *et al*. and none for Chen *et al*.(Table S8). As shown in Table S8, RCC did a better job of finding cancer subtypes with significantly distinct survival profiles. The survival profiles of the top five cancers (KIRC, LGG, SARC, SKCM, and UCEC) are shown in Figure S5. An elaborate example of the same is SARC, which further divides into four clusters. All the four SARC clusters show significantly distinct survival profiles with cluster four having the best prognosis and cluster three having the worst. SSGSEA analysis with the cancer hallmarks gene set^22^ of SARC clusters showed that each of the clusters have gene sets with distinct enrichment. Cluster one shows enrichment in glycolysis and DNA repair gene sets, cluster two shows enrichment in myogenesis gene set, cluster three shows enrichment of genes down-regulated in KRAS pathway and genes involved in Hedgehog signaling whereas cluster four shows enrichment in complement pathway and apoptosis gene sets (Figure S6, Table S9)

**Figure 5:**
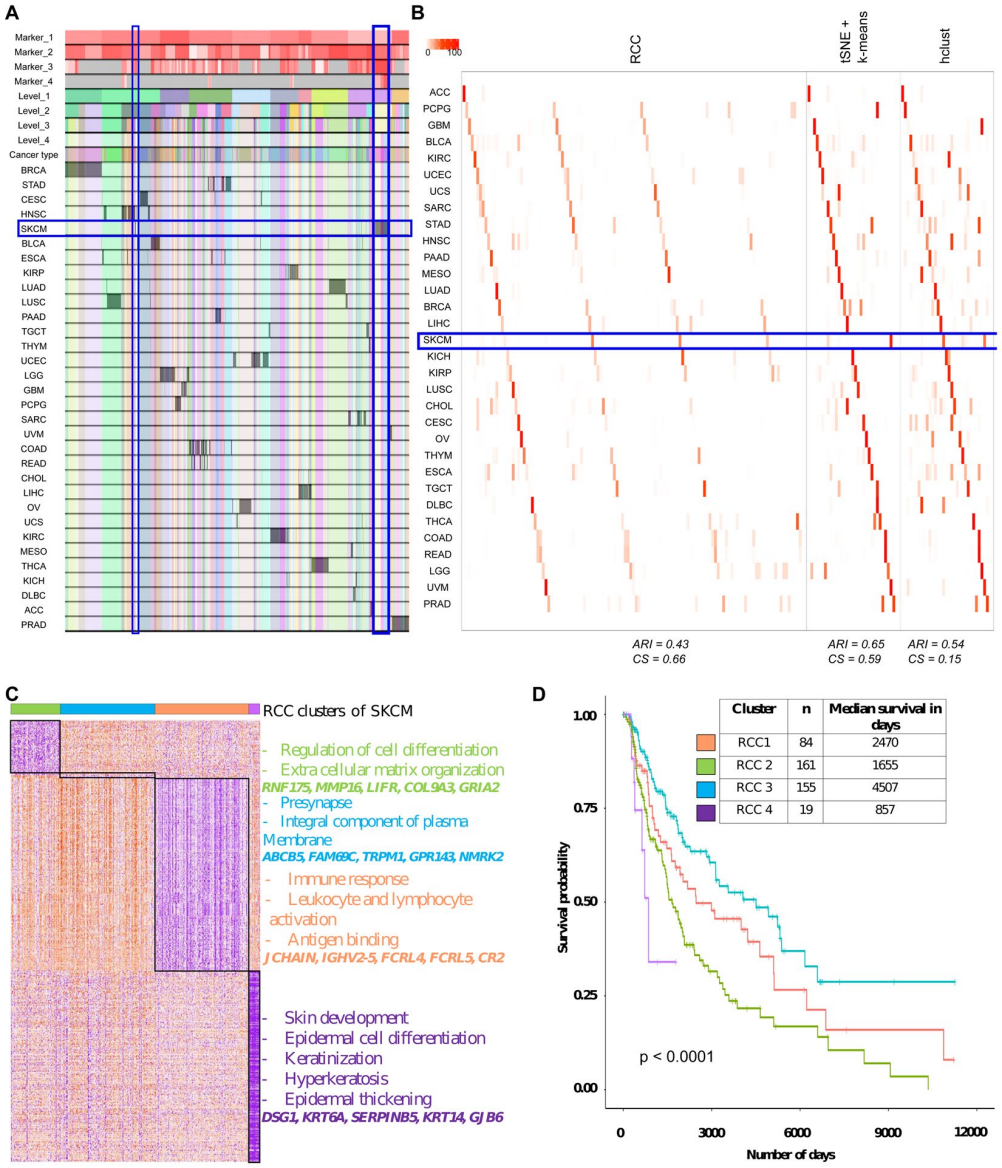
TCGA pan-cancer data. (**A**) Tracking plot for TCGA pan-cancer data showing the multi-level clustering information. The SKCM samples are highlighted in the blue box. At level one the division of samples is more tissue-specific and then at further levels, it finds the novel subtypes. (**B**) Cluster annotation plots for RCC, t-SNE + k-means, and hclust algorithms. Each plot shows the overlap between the annotations and clusters found. Along the x-axis is the clusters found by each algorithm and along the y-axis are the cancer types. Red indicates a 100% overlap between the annotated class and the cluster and white indicates no overlap. The SKCM clusters are highlighted in the blue box. RCC and hclust divide the SKCM samples into four clusters whereas tSNE + k-means divides the data in two clusters. (**C**) Cluster specific markers of SKCM clusters showed that each of the clusters has distinct marker genes. Cluster one shows enrichment of genes involved in the regulation of cell differentiation and ECM organization, cluster two shows involvement in presynapse and an integral component of the plasma membrane, cluster three shows involvement in immune response, leukocyte and lymphocyte activation and antigen binding, and cluster four shows enrichment in skin development, epidermal cell differentiation, keratinization, hyperkeratosis, and epidermal thickening (gene marker list available in Table S7). (**D**) Survival profiles for SKCM samples based on their cluster assignment. All the four SKCM clusters show significantly distinct survival profiles with cluster three having the best prognosis and cluster four having the worst.

### Single-cell RNA-Seq data

1. **Biase dataset:** Biase dataset^13^ consists of single-cell RNA-seq data of matched sister blastomeres. The single-cell data was generated for zygotes, 2-cell, 4-cell mouse embryos and blastocytes. The original publication discussed 5 subtypes of the mentioned data. RCC consistently found all the five subtypes across 1000 runs (Fig. 2B). Biase *et al*. found two subtypes of the mouse blastocytes namely inner cell mass (ICM) and trophectoderm (TE). Both tSNE + k-means and SC3 were not able to find distinct clusters of these subgroups whereas RCC was consistently able to divide these cells into two clusters at level 2, again highlighting the power of recursive clustering (Fig. 6A).

**Figure 6:**
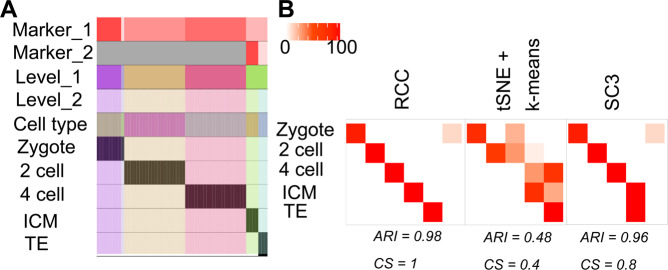
Biase data. (**A**) Tracking plot for Biase data showing the multi-level clustering information. (**B**) Cluster annotation plots for RCC, t-SNE + k-means, and SC3 algorithms. Each plot shows the overlap between the annotations and clusters found. Along the x-axis is the clusters found by each algorithm and along the y-axis are the cell-types. Red indicates a 100% overlap between the annotated class and the cluster and white indicates no overlap.
2. **Pollen dataset:** Pollen dataset^14^ includes single-cell data of 11 cell types which can be broadly divided into four subtypes namely: blood cells, dermal/ epidermal cells, neural cells, and pluripotent cells. All the cell types are grouped individually at level one itself. Further, at level two, it was observed that the BJ cell line which is pluripotent stem cells (hiPSCs) originally derived from neonatal male human foreskin fibroblasts are consistently getting subdivided into three clusters across multiple runs (Fig. 7B,C). These subgroups of BJ cells were not discovered by tSNE + k-means or SC3 algorithms (Fig. 7B). On further analysis we found that cluster two consisted of cells with expression of genes involved in RNA binding, structural constituent of ribosome and establishment of protein localization to the endoplasmic reticulum, cluster three showed enrichment of genes involved in post-embryonic development and post-transcriptional regulation of gene expression, whereas cluster one had cells which did not show enrichment of genes up-regulated in either of the clusters (Fig. 7D, Table S10).

**Figure 7:**
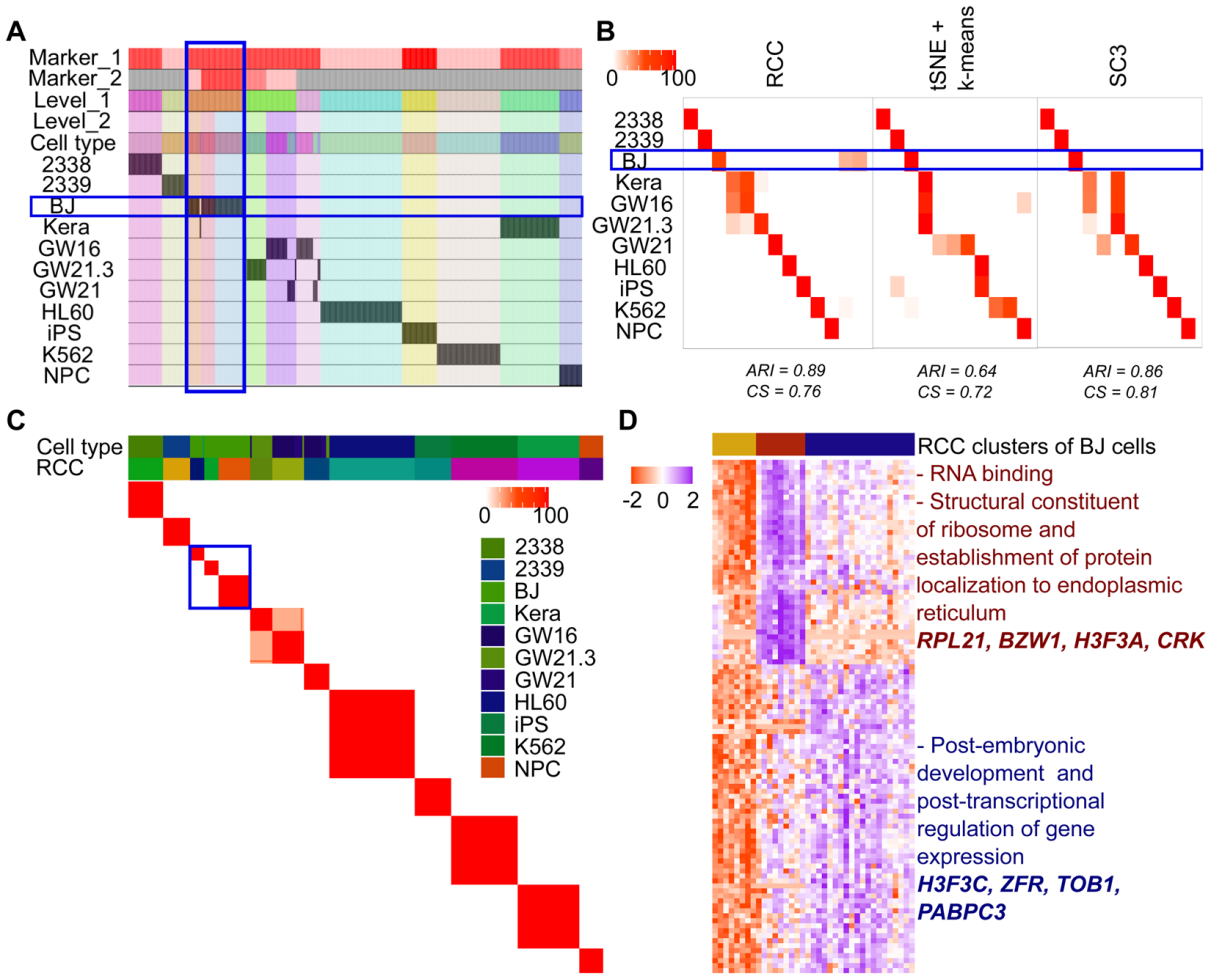
Pollen data. (**A**) Tracking plot for Pollen data showing the multi-level clustering information. The BJ cell line is highlighted in the blue box. At level one, RCC divides the cells based on their cell types. Further recursive clustering of data finds the subtypes of the BJ cell line. (**B**) Cluster annotation plots for RCC, t-SNE + k-means, and SC3 algorithms. Each plot shows the overlap between the annotations and clusters found. Along the x-axis is the clusters found by each algorithm and along the y-axis are the cell-types. Red indicates a 100% overlap between the annotated class and the cluster and white indicates no overlap. The BJ cell line clusters are highlighted in the blue box. RCC found three clusters of the BJ cell line with distinct gene markers. These clusters were not found by tSNE + k-means and SC3. (**C**) Consensus Cluster Matrix for 1000 runs of RCC showing consistent subgroups of BJ cell line highlighted by the blue box. (**D**) Gene marker plot of BJ cell-types where cluster two consisted of cells with expression of genes involved in RNA binding, structural constituent of ribosome and establishment of protein localization to the endoplasmic reticulum, cluster three showed enrichment of genes involved in post-embryonic development and posttranscriptional regulation of gene expression, whereas cluster one had cells which did not show enrichment of genes upregulated in either of the clusters (gene marker list is available in Table S10).
3. **Darmanis dataset:** Darmanis *et al*.^15,16^ have generated two single-cell RNA-Seq datasets: One in 2015 using human adult cortical samples and another one in 2017 using four glioblastoma multiforme (GBM) patient samples. We combined both the datasets which include 4055 cells and 15 known cell types in total. RCC found 43 clusters across these cell types (Fig. 8A). RCC divided the non-malignant cells based on their individual cell types irrespective of their source tissue whereas the malignant cells showed to be divided by the patient types as one would expect. RCC also divided the oligodendrocytes (2015 and 2017 combined) into three subtypes (Fig. 8B). The oligodendrocyte clusters showed enrichment of kinase binding and mRNA splicing genes in cluster one, regulation of protein polymerization, myelin sheath and ferric ion binding genes in cluster two and ganglioside GT1b binding, membrane raft polarization and myelination genes enrichment in cluster three (Fig. 8C, Table S11). Though tSNE + k-means and SC3 can differentiate between the oligodendrocyte cells from adult cortical samples and GBM samples, they do not subdivide the GBM oligodendrocytes. Similarly, RCC also divides endothelial and fetal quiescent cells into three clusters each (Fig. 8D,E, Tables S12 and S13). In endothelial cells, cluster one shows genes involved in the blood vessel and vascular development processes, cluster two shows cell adhesion specific gene markers involved in blood vessel morphogenesis process and, cluster three markers showed to be playing a part in extracellular matrix formation (Table S12). tSNE + k-means divides the endothelial cells in two clusters, whereas SC3 forms only a single cluster of all the endothelial cells. In fetal quiescent cell types, cluster two shows enrichment of genes involved in axon guidance pathway and semaphorin receptor activity, cluster three shows enrichment of genes involved in kinase activity and neurotrophin TRKC receptor binding whereas cluster one had cells which did not show enrichment of genes up-regulated in either of the two clusters (Table S13). RCC clusters the fetal quiescent and fetal replicating cells together at level one, but at level two they are further subdivided into their individual clusters. tSNE + k-means and SC3 algorithms were not able to differentiate between these two cell types.

**Figure 8:**
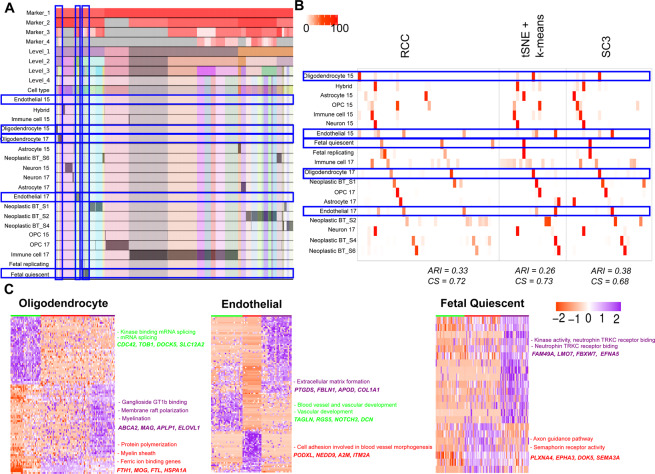
Darmanis data. (**A**) Tracking plot for Darmanis data showing the multi-level clustering information. The oligodendrocyte, endothelial, and fetal quiescent cell types are highlighted in the blue box. RCC divides the data into four clusters at level one. RCC then proceeds to recursively divide the cells into individual groups at further levels. (**B**) Cluster annotation plots for RCC, t-SNE + k-means, and SC3 algorithms. Each plot shows the overlap between the annotations and clusters found. Along the x-axis is the clusters found by each algorithm and along the y-axis are the cell-types. Red indicates a 100% overlap between the annotated class and the cluster and white indicates no overlap. The oligodendrocyte, endothelial, and fetal quiescent clusters are highlighted in the blue box. RCC divides oligodendrocytes, endothelial, and fetal quiescent cells into three clusters each. tSNE + k-means and SC3 are not able to find these novel cell types clusters. (**C**) Gene marker plot for oligodendrocytes, endothelial, and fetal quiescent cells in the Darmanis dataset (Gene marker list available in Tables S10, S11, S12). The clusters are formed irrespective of their origin (i.e. from the normal brain or tumor samples). The oligodendrocyte clusters show enrichment of kinase binding and mRNA splicing genes in cluster one, regulation of protein polymerization, myelin sheath, and ferric ion binding genes in cluster two and ganglioside GT1b binding, membrane raft polarization and myelination genes enrichment in cluster three. In Endothelial cells, cluster one shows genes involved in the blood vessel and vasculature development processes, cluster two shows cell adhesion specific gene markers involved in blood vessel morphogenesis process and, cluster three markers showed to be playing a part in an extracellular matrix formation. In Fetal Quiescent cells, cluster two shows enrichment of genes involved in axon guidance pathway and semaphorin receptor activity, cluster three shows enrichment of genes involved in kinase activity and neurotrophin TRKC receptor binding whereas cluster one had cells which did not show enrichment of genes up-regulated in either of the two clusters.
4. **Neftel dataset:** Neftel dataset^17^ includes single-cell RNA- sequencing of 28 glioblastoma tumors (pediatric and adult). The dataset we used has 7930 cells and 31 different known (three non-malignant and 28 patient wise malignant) cell types. Neftel *et al*. showed that the malignant GBM cells are majorly found in four cellular states which are determined by six meta modules based on the following gene signatures; Hypoxia independent (MES1like) and hypoxia dependent (MES2like) mesenchymal related gene sets, astrocytic (AClike) marker gene set, oligodendroglial (OPClike) lineage marker gene set, and stem and progenitor cell signatures (NPC1like and NPC2like) as well as two cell cycling modules namely G1S and G2M. They concluded after extensive analysis that there are largely four cellular states of glioblastoma cells (MES, AC, OPC, NPC) and each patient has varying proportions of these states, the proportions which are influenced by genetics and microenvironment. At the gene expression level, the cells are more similar to each other based on their cellular state rather than based on their parent tumor. Results of RCC recapitulates these findings as can be seen in the pie charts demonstrating cells from each tumor clustering based on their meta module score (Figs. 9C and S7). RCC divides the data in two clusters at level one where cluster one includes the immune cell types and cluster two includes the malignant cells (Fig. 9A). At level two of RCC clustering, it divides the malignant cell types based on the source tissue i.e patient types. Due to the recursive nature of RCC, it is also able to further divide the patient specific clusters to find the meta module specific subtypes. Based on the findings of the original paper if we take as to attribute the cellular state which is defined by the highest meta module score for each cell and label each cluster based on the highest average meta-module score for cells in that cluster, then we can see that RCC clusters cells with similar cellular state well (Fig. 9C). Figure 9C shows the concordance between the single-cell module assignment vs. the cluster module assignment. RCC clusters are more homogeneous with respect to the cellular states of cells as compared to the other algorithms. tSNE + k-means, as well as SC3, are not able to identify clusters of highly proliferating cells as determined by the overepxression of cell cycling genes (highlighted in Fig. 9C). We are also able to find subtypes in non-malignant cells not described in the original study. At level two of clustering, the macrophage clusters reflect different cell populations which are either microglia like (brain resident) or macrophage-like (blood-derived)^20^ and are in different tumor microenvironment (PAN - hypoxic vs. LE - non-hypoxic) (Fig. 9D, Table S14).

**Figure 9:**
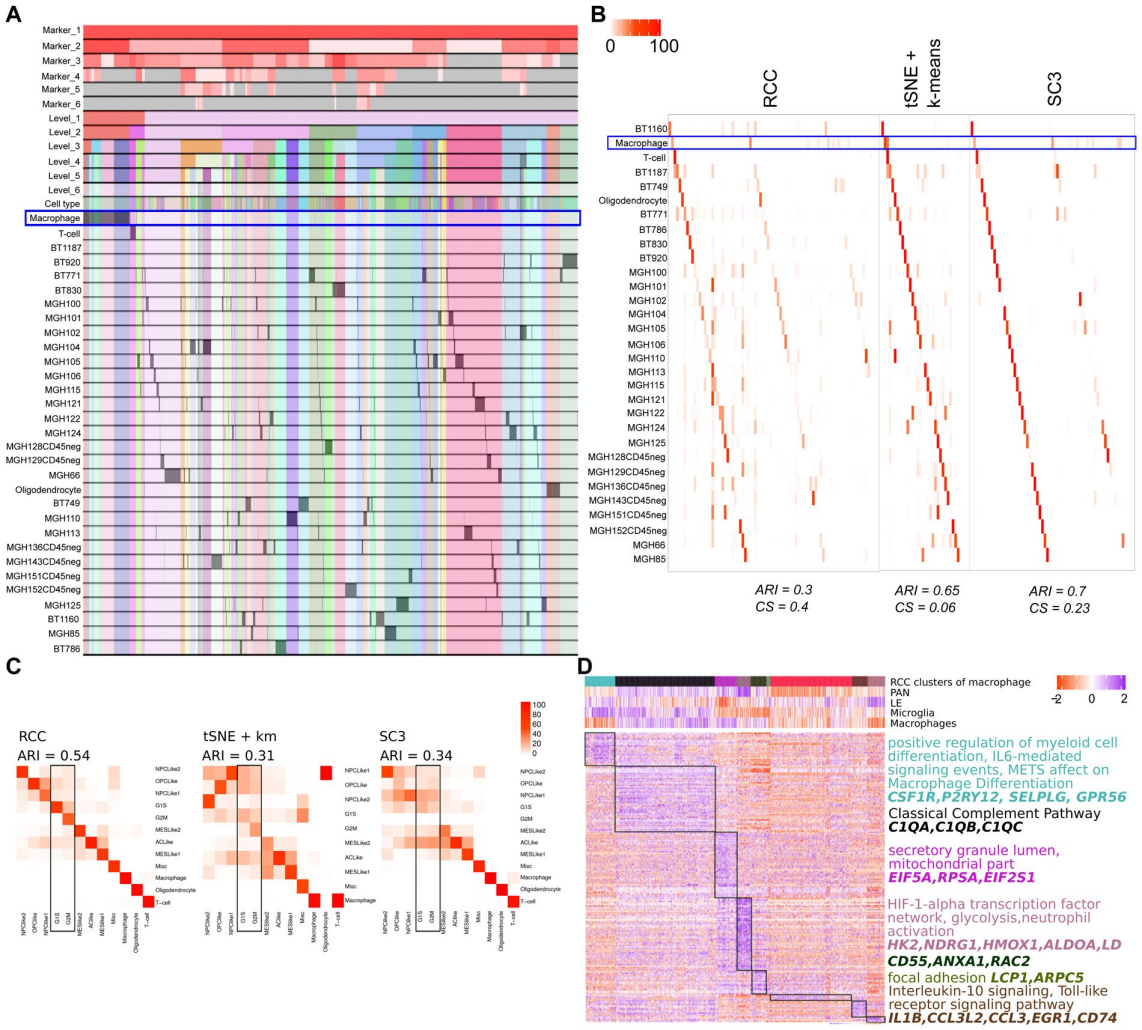
Neftel data: (**A**) Tracking plot for Neftel data showing the multi-level clustering information. The macrophage cells are highlighted in the blue box. RCC divides the data into two clusters at level one with cluster one having immune cells and cluster two having the malignant cells. RCC further recursively clusters these clusters into various sub-clusters where the malignant cells are divided based on the source tissue. (**B**) Cluster annotation plots for RCC, t-SNE + k-means, and SC3 algorithms. Each plot shows the overlap between the annotations and clusters found. Along the x-axis is the clusters found by each algorithm and along the y-axis are the cell-types. Red indicates 100% overlap between the annotated class and the cluster and white indicates no overlap. The macrophage clusters are highlighted in the blue box. RCC divides the macrophages into eight clusters whereas tSNE + k-means and SC3 divides the macrophages into three and nine clusters respectively. (**C**) Cluster annotation plots for RCC, tSNE + k-means and SC3 with cellular states on the x-axis and cluster label representing the dominant cellular state on the y-axis. (**D**) Markers for macrophage clusters and their enrichment across nine clusters. The annotation panel shows the SSGSEA score for tumor periphery (LE - leading edge), hypoxia (PAN - pseudopalisading cells around necrosis), microglia, and macrophage gene sets.

## Discussion

Large-scale transcriptomic data and especially single-cell sequencing have now become the mainstay tools for understanding molecular mechanisms in disease and developmental biology. With datasets ranging from a few hundred samples to thousands (millions in case of single-cell data), there is a significant challenge in analyzing these datasets to gain biological insights. Clustering is always used for such analyses but automatically identifying the underlying number of clusters remains a challenge. A typical pipeline that is used involves taking known annotation of samples and then selection of clusters using manual identification through visualization. The problem with this approach is 1. reliance on known information when the objective is to unearth new information 2. Partial exploration of data and 3. An extensive amount of work requiring computational expertise. To address these limitations of current clustering methods, we introduce the concept of recursive clustering with an approach to automatically identify the optimal number of clusters in transcriptome datasets. We show that using our algorithm, RCC, available as an R package, with just a few commands we can recapitulate findings obtained through multi-step complex analysis and also identify additional subtypes not described in the original studies. We can fully explore the datasets in just a few steps without the need for domain knowledge. We have compared our method to the most popular methods and shown that we are uniquely able to identify novel subtypes and interpret results conveniently by visualization of intuitive output plots. Also, it works equally well for both bulk and single-cell data.

One of the limitations of our algorithm is computational time. This time is dependent on the underlying base algorithm which currently is consensus clustering with k-means. As new efficient clustering algorithms become available, k-means can be replaced by those for improved performance. To truly harness the power of single-cell transcriptomics for a better understanding of biology, the next step is the analysis of large integrated datasets with millions of cells. Our method, when combined with methods like scanorama for integration of multiple datasets^23^ and geometric sketching for subsampling of data that preserves heterogeneity^24^, will be an essential approach to maximize the gain of biological insights from large-scale transcriptomic datasets.

In conclusion, RCC is a user-friendly data clustering algorithm that can be used on both bulk and single-cell transcriptomic datasets. RCC analysis facilitates novel subtype discovery in the transcriptomic data allowing the user to tease out the unknown finer structure in large datasets through intuitive visualization of clustering results.

## Methods

### RCC algorithm

RCC algorithm has six major steps: 1) Data input 2) Feature selection and data scaling 3) Parallel processing of ConsensusClusterPlus 4) Optimal *k* selection 5) Recursive clustering and 6) Output. Each step is described in detail below.

1. **Data input:** RCC takes an expression matrix (X) and configuration file as input. The expression matrix is a *csv* file where the columns represent samples/cells and the rows represent expression values for genes/transcripts. The input matrix should be normalized and log-transformed after adding a pseudo count of 1. Along with the expression matrix, the user also has the option of uploading a sample information file which contains clinical information or attributes for the samples/cells to be clustered. The configurable parameters with default values are discussed at the end of this section.
2. **Feature selection and data scaling:** The current clustering algorithms either use all the available genes or top variant genes or top principal components (PCA) or Single-cell Hierarchical Poisson Factorization (ScHPF)^25^ factors which are low dimensional representations of the datasets. These methods give us only a global view of a dataset and may not reveal the finer subgroups within the dataset. In RCC, initially all samples and top n% variant genes are selected, then normalized and clustered as described in steps 3–4. Based on the previous level of clustering, the feature set changes for subsequent recursive runs. We tested multiple datasets with different top n% variant genes and found that the top 1% of variant genes work well for the single-cell transcriptomic data, whereas the top 3–5% genes work well for bulk tissue data. This is a user-defined parameter in RCC where the user can select top n% of variant genes/features to be selected for further clustering. For a bulk-transcriptome dataset, a minimum of 500 genes/features are required for further clustering. If the top n% variant genes/features are <500, RCC by default takes the top 500 variant genes for further analysis. For single-cell transcriptomic data the number of genes detected per cell can be as low as 200 hence no minimum criteria is applied. After subsetting the top n% variant genes/features, data scaling is done using the following formula:
  $$z=X-\mu $$
  where X is the expression measure of a gene in a cell/sample, μ is the mean expression measure of a gene across all the cells/samples and σ is the standard deviation of expression measure of a gene across all the cells/samples.
3. **Parallel processing of ConsensusClusterPlus:** The base algorithm used for RCC is ConsensusClusterPlus^14^ (CCP) which performs k-means clustering on the dataset. CCP is run in parallel eight times with a different seed each time for increased robustness. We chose eight parallel runs of RCC, with each run implementing *n* iterations of CCP as it reduced the processing time and improved the robustness of algorithm. The output of CCP includes stability evidence for a given number of clusters (*k*) and their assignments. CCP is run each time with the following parameters:
  Maximum number of clusters: The maximum number of clusters (*maxK*) for each run is calculated by taking the minimum between 10 and 1/10th of the number of samples. *maxK* changes for each recursive run of RCC. We limit the *maxK* to 10 because based on our testing, the best *k* selection is not reliable for larger *k*.
  Number of repeats for each run: Each of the eight parallel processes has a repeat count of 100.
  Proportion of items and features to sample (pItem and pFeature): As discussed earlier, RCC runs CCP in parallel eight times to get stable clusters. To enable selection of the most robust clusters, the pItem and pFeature values are changed for every run. The selected pItem values range from 0.6–0.9 whereas the pFeature values used are 0.8 and 1. Different combinations of the mentioned values are as shown in Table S1.
  Clustering algorithm: RCC uses k-means algorithm with Euclidean distance. K-means is applied on the z-scored data matrix with the Hartigan and Wong algorithm. By default, the number of centers is set to 10 and the maximum number of iterations is set to 10^9 in the k-means function.
  Seed: The k-means clustering algorithm requires an initial seed number to generate clusters. The initial seed value is very crucial for the clustering of data as it effects the repeatability and reproducibility of the results. To find robust clusters RCC generates random seed values for all of the eight parallel runs of CCP.
4. **Optimal** ***k*** **selection:** Based on the cumulative distribution function (CDF) plots produced by CCP, RCC finds the best *k*^10^ (Simulated data in methods) for each parallel process as described below in turn giving eight *k* values. The *k* with maximum frequency is selected as the optimal *k* for that run of RCC. An example CDF plot of consensus matrices for *k* = 2:6 is shown in Figure S2. The consensus matrix of *N* samples for 100 runs of clustering is an *N x N* matrix with each cell containing the number of times the row and column samples cluster together. For a given consensus matrix *M*, CDF is defined over the range of 0 to 1.

$$CDF(c)=\frac{{\sum }_{i < j}\{M(i,j)\leqslant c\}}{N\times (N-1)/2}$$

Consensus index *c* varies from 0 to 100. The perfect CDF plot for a given *k* (number of clusters), where for every run we get the same result, will have a CDF line with slope 0° starting at *c = 0* and ending at *c = 99*. The perfect CDF value will be:

$$CDFp(0-99)=1-\mathop{\sum }\limits_{i=1}^{k}{N}_{i}\times ({N}_{i}-1)/2$$

Figure S2 shows an example of CDF plots for *k* = *2,3,4,5,6*. Top panel shows the consensus matrices for each *k* and the bottom panel shows the CDF plot with slope and line length calculations. The dot- dash line shows the maximum CDF value for each *k*.

#### Line length and slope calculation

The clustering of real data typically does not result in perfect CDF value so we create an allowance of **±**0.5. We take all CDF values in that range and fit a line through it. If the slope of the line is > minimum slope threshold, we trim the line till we get the slope below threshold or the line length is smaller than the minimum line length.

#### Intra cluster stability calculation

$${\rm{I}}{\rm{n}}{\rm{t}}{\rm{r}}{\rm{a}}\,{\rm{c}}{\rm{l}}{\rm{u}}{\rm{s}}{\rm{t}}{\rm{e}}{\rm{r}}\,\mathrm{stability}\,={\bar{M}}_{i,j}\,{\rm{w}}{\rm{h}}{\rm{e}}{\rm{r}}{\rm{e}}\,i,j\in \mathrm{same}\,{\rm{c}}{\rm{l}}{\rm{u}}{\rm{s}}{\rm{t}}{\rm{e}}{\rm{r}}$$

#### Inter cluster overlap calculation

$${\rm{I}}{\rm{n}}{\rm{t}}{\rm{e}}{\rm{r}}\,{\rm{c}}{\rm{l}}{\rm{u}}{\rm{s}}{\rm{t}}{\rm{e}}{\rm{r}}\,\mathrm{overlap}\,={\bar{M}}_{i,j}\,{\rm{w}}{\rm{h}}{\rm{e}}{\rm{r}}{\rm{e}}\,i,j\in {\rm{d}}{\rm{i}}{\rm{f}}{\rm{f}}{\rm{e}}{\rm{r}}{\rm{e}}{\rm{n}}{\rm{t}}\,{\rm{c}}{\rm{l}}{\rm{u}}{\rm{s}}{\rm{t}}{\rm{e}}{\rm{r}}{\rm{s}}$$

#### Differentially expressed genes

K-means clustering algorithm tends to find stable clusters even when the sample distribution is random but asymmetric resulting in sub-classification without biological relevance^22^. This parameter helps in selecting only biologically meaningful clusters for a dataset. To find the clusters with biological significance, RCC calculates differentially expressed genes across all the clusters for a given run. The clustering is selected only if it has n% of genes up regulated with FDR <0.01 in at least one of the clusters.

#### Best *k* selection

We select the best *k* based on the following criteria:

- Line length is > minimum line length (default: 30)
- Line slope is < minimum slope threshold (default: 10°)
- Intra cluster stability > 0.8
- Inter cluster overlap < 0.2
- Differentially expressed genes > minimum % of genes in at least one of the clusters (default: 20% for large dataset, 10% for small dataset)
- If multiple *k* values are selected then we break the tie assigning weights to each *k*. We assign weights to each *k* if the slope of k is ≤ 5° and if line length ≥ 40. The *k* with maximum weight is selected as the best *k* (if multiple *k*s have equal weight all of them are selected as the best *k*).

In the example Figure S2, it can be seen that *k = 2,3,4,5* satisfy the criteria a:d. All the *k*s also satisfied criteria e. Based on f, *k* = *3,4,5* is selected as the best *k*. We get similar values from the remaining eight runs which in turn gives us an array of best *k*s. The optimal *k* is selected out of these best *k*s based on frequency distribution. The best *k* with the highest frequency is selected as the optimal *k*.

5. **Recursive clustering:** Once the optimal *k* for a particular level is selected, each of the subdivided datasets go on for further clustering. The samples/cells get recursively clustered until one of the following criteria is met:
  Number of samples/cells in the dataset is lesser than the minimum number of samples required for clustering
  Optimal *k* is zero; I.e no best *k* is selected in all the parallel runs of RCC.
6. **Output:** Upon submission of an expression matrix and their respective annotations, RCC gives the following output:
  **Cluster information file (ClusterInfo.csv):** The basic output of RCC is the cluster information file which has the cluster assignment of all the samples in the submitted datasets at every level along with the final cluster assignment.
  **Cluster annotation plot (atrribute_vs_algorithm.pdf):** The cluster annotation plot allows the user to view the cluster assignment of each sample based on a particular attribute using the *clusterAnnotation* function. The Cluster annotation plot shows the distribution of all the attributes (e.g. cell types or tissue types) across all the clusters. The columns represent the attributes present in the dataset and the rows represent the clusters (Figure S3).
  **Tracking plot (trackingPlot.pdf):** The *trackingPlot* function is a visualization tool that allows the user to view the clustered data in an easy and interpretable manner (Figure S4). Columns in the tracking plot correspond to the samples/cells. The first panel *i* is the marker panel which shows the number of marker genes identified for each cluster at a given level. The first row is markers for clusters found at level one, the second one for markers found at level two, and so on. The color-scale is from white to red with white indicating zero markers found and bright red indicating the highest number of markers found. Grey color indicates that the particular cluster is not further divided and hence no new markers are found which are distinct from the previous level. The second-panel *j* is the RCC clustering level panel. It shows the cluster information for samples at each level starting with level one. The third- panel *k* is the annotation panel. The annotation panel shows the distribution of samples across all the clusters.
  **Gene markers plot (Level_markers.pdf):** The *geneMarkers* function in RCC calculates the specific genes which are significantly up regulated in the clusters at each level with FDR <0.01 and log2 fold change values> 1. These genes are the specific markers for their representative clusters. RCC calculates and plots the markers across the levels making it easier for the user to visualize the data clustering. In the marker plots, the columns represent the samples/cells and the rows represent the genes.
  **SSGSEA analysis (ssgsea.pdf):** To find the biological significance of the clusters, RCC allows the user to perform a single sample gene set enrichment analysis (SSGSEA)^26^ using the function *ssgsea*. This function allows the user to find the enrichment of particular gene sets in all the samples of the clusters. The user can input the gene sets of their importance in CSV format to perform the SSGSEA analysis. The SSGSEA heatmap shows the enrichment of all the gene sets across all the samples. In the plot, the columns represent the samples/cells and rows represent the gene sets. For example, Figure S6 shows the SSGSEA plot of sarcoma samples from the TCGA dataset which allows us to look into the enrichment of cancer hallmarks in each cluster.
  **Cluster attribute enrichment analysis (atrribute_vs_algorithmFE.csv):** When a sample information file that contains clinical information or attributes is provided the user can perform cluster attribute enrichment analysis using the function *clusterAttr*. This function implements Fisher’s exact test to find a significant correlation between the attributes (categorical variable) and clusters. This helps in understanding the biological/clinical significance of the cluster.
  **Kaplan**–**Meier analysis (survival.pdf):** RCC allows the user to perform survival analysis for all the clusters using the function *clusterSurvival*.

Output of RCC analysis described in this manuscript are available as Supplementary Data. All the gene enrichment analysis was done using Toppfun^27^.

### RCC configurable parameters

Along with the input matrices, RCC also requires an input configuration file. This file allows the user to adjust the clustering parameters or use the default ones based on their requirements. The configuration file is in the*.csv* format where the first column indicates the parameter name and second column indicates the parameter value. The configurable parameters are:

1. Input expression matrix.
2. Input annotation file: This is an optional parameter. If one does not have any annotation for the sample dataset this field can be left as NA.
3. Minimum slope threshold: The default value is 10°. Lower the threshold tighter the clustering. Recommended values are between 5–15°.
4. Minimum number of samples required for clustering: This option allows the user to decide the minimum sample size that is sufficient for further sub grouping. The default value is 20. A smaller number will likely result in a larger number of clusters.
5. Minimum line length: The default value is 30. Perfect clustering will have a line length of 99. Higher the value tighter the clusters. Recommended values are between 30–60.
6. Percentage of genes/features to be used for clustering: We use the top 3–5% of variant genes in bulk data and the top 1% of variant protein-coding genes in single-cell data for clustering. For bulk data, the recommended values are between 2–5% and for single-cell data, it is 1–2%.
7. Minimum percentage of genes that are differentially expressed: The default value for large datasets (i.e. datasets having more than 1000 cells/samples) is 20% and for small datasets, it is 10%.
8. Type of dataset: RCC accepts both bulk as well as single-cell transcriptomic datasets. This parameter is used by RCC for determining feature selection criteria. If the data is bulk tissue, then a minimum of 500 or top n% genes/features (whichever is higher) are taken for clustering.
9. Output directory: Absolute path to the folder where the user wants to output the results

### **Best*k*selection using simulated datasets**

We generated simulated datasets using the CIDR^28^ package in R to check if RCC was able to select the best *k*. Two simulated matrices were generated where *mat1* had five sub- groups and each subgroup had an exact number of samples in them, whereas *mat2* had five subgroups with varying proportions of samples in them. The best *k* found by RCC is the same as true *k* in the simulated dataset (Figure S8).

### Cluster stability

To check for the stability of our algorithm we ran RCC 1000 times on Ivy GAP, Biase and Pollen datasets, 100 times on the Darmanis dataset and 10 times on Human tissue, TCGA pan-cancer, and Neftel datasets. We calculated ARI (Fig. 2A) and plotted the heatmap of the Consensus Clustering Matrix to see the consistency of clustering in dataset (Fig. 2B, Supplementary data).

### Level cutoff

The novel feature of RCC is its recursive nature. RCC keeps dividing the data until there is no significant variance in it. Once RCC is run, the user can use the tracking plot generated by RCC to visualize the clustering of the dataset for sample attributes. The tracking plot provides an intuitive and convenient way to interpret the results and derives biological insights. In case the user requires clustering up to a particular level as any further clustering might not be relevant for the user, they can use the *cutoff* function to allow RCC to cluster the data points only up to a particular level. This feature allows the user to control the subdivision of their data where needed. Figure S9 shows the before and after cutoff clustering for the TCGA pan-cancer data. Initially, RCC finds a total of 138 clusters with division up to four levels. After applying the *cutoff* function at level three, RCC finds a total of 118 clusters.

### System configurations

All the algorithms were run on a computer with an Intel Intel® Xeon(R) CPU E5–2630 v4 processor running at 2.20 GHz × 40 using 251.8 GiB of RAM, running Ubuntu version 16.04.

## Availability of data and materials

Project name: RecursiveConsensusClustering

Project homepage: https://github.com/MSCTR/RecursiveConsensusClustering

Operating system: Platform independent

Programming language: R

License: GPL

Any restrictions to use by non-academics: None

Datasets used: https://www.msctr.org/2019/05/30/recursive-consensus-clustering/

Datasets:

**Table:** 

Dataset	Link
Ivy GAP	http://glioblastoma.alleninstitute.org/
Human tissue data	https://www.gtexportal.org/home/datasets
TCGA pan-cancer data	https://portal.gdc.cancer.gov/
Biase	GSE57249
Pollen	SRP041736
Darmanis	GSE67835 and GSE84465
Neftel	GSE131928

## Supplementary information

Supplementary information.
