# Supplementary material for: Recursive Consensus Clustering for novel subtype discovery from transcriptome data
Source: Sci Rep. 2020 Jul 3;10:11005. doi: 10.1038/s41598-020-67016-3 (PMC7335086; doi:10.1038/s41598-020-67016-3)
Supplement: Supplementary file 1 — Supplementary information. [file 41598_2020_67016_MOESM1_ESM.pdf]

Recursive Consensus Clustering for novel subtype discovery from transcriptome data

Pranali Sonpatki[1] & Nameeta Shah[1]*

[1]Mazumdar Shaw Center for Translational Research, Mazumdar Shaw Medical Foundation, Narayana Hrudaya- laya Health City, Bangalore, India

[*]nameeta.shah@gmail.com

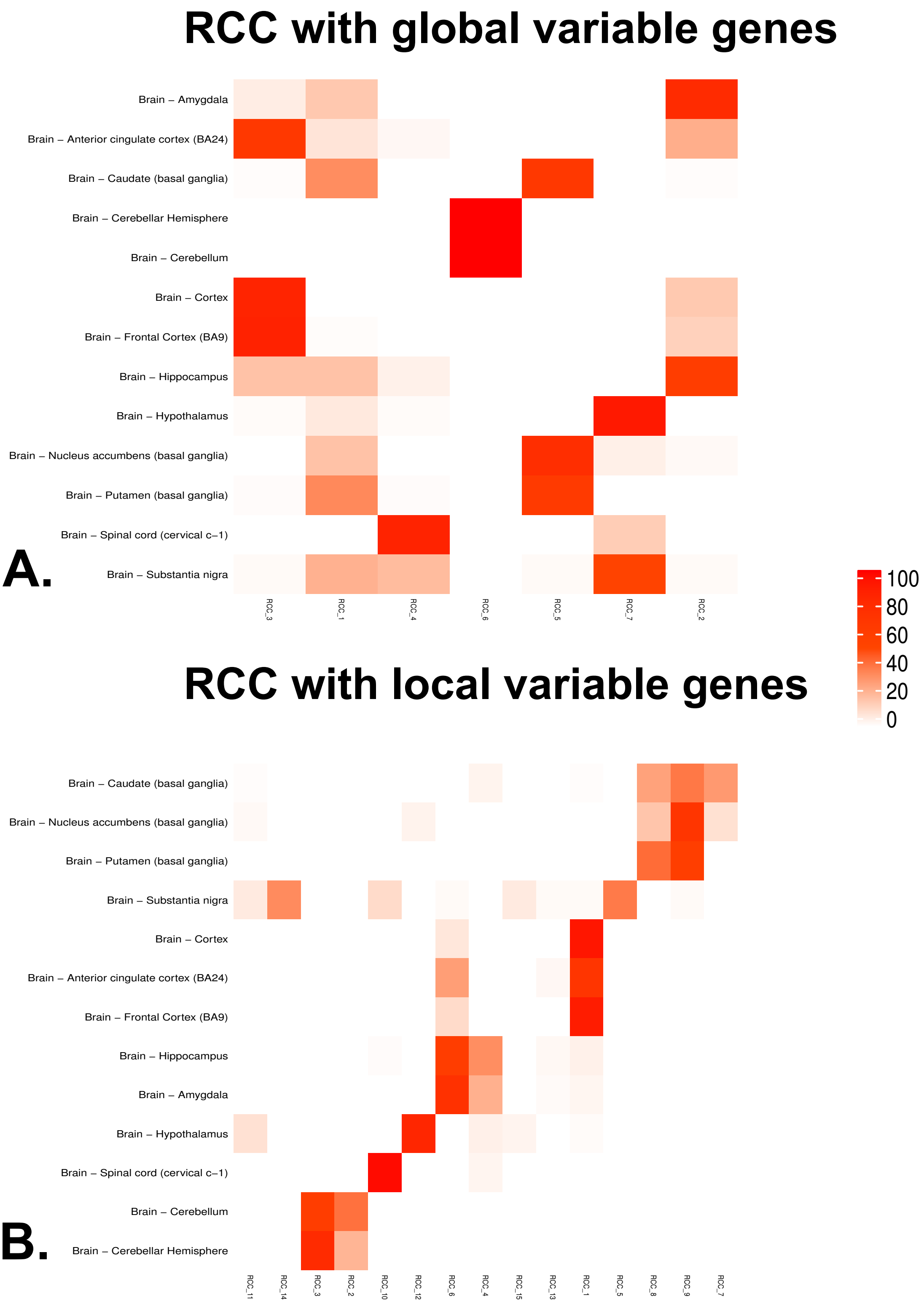

Figure S1: *Feature selection in RCC. A) RCC clustering of Brain tissue data using the top 2% variable genes across 53 tissue types. B) RCC clustering of Brain tissue data using the top 2% variable genes in the brain tissue samples. The selected features changing based on the current clustering hierarchy instead of being constant based on the entire dataset results in discovery of the finer structures of the data.*

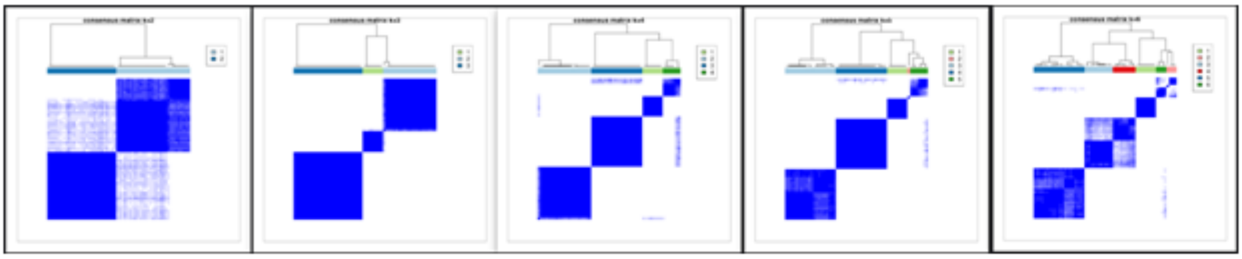

A.

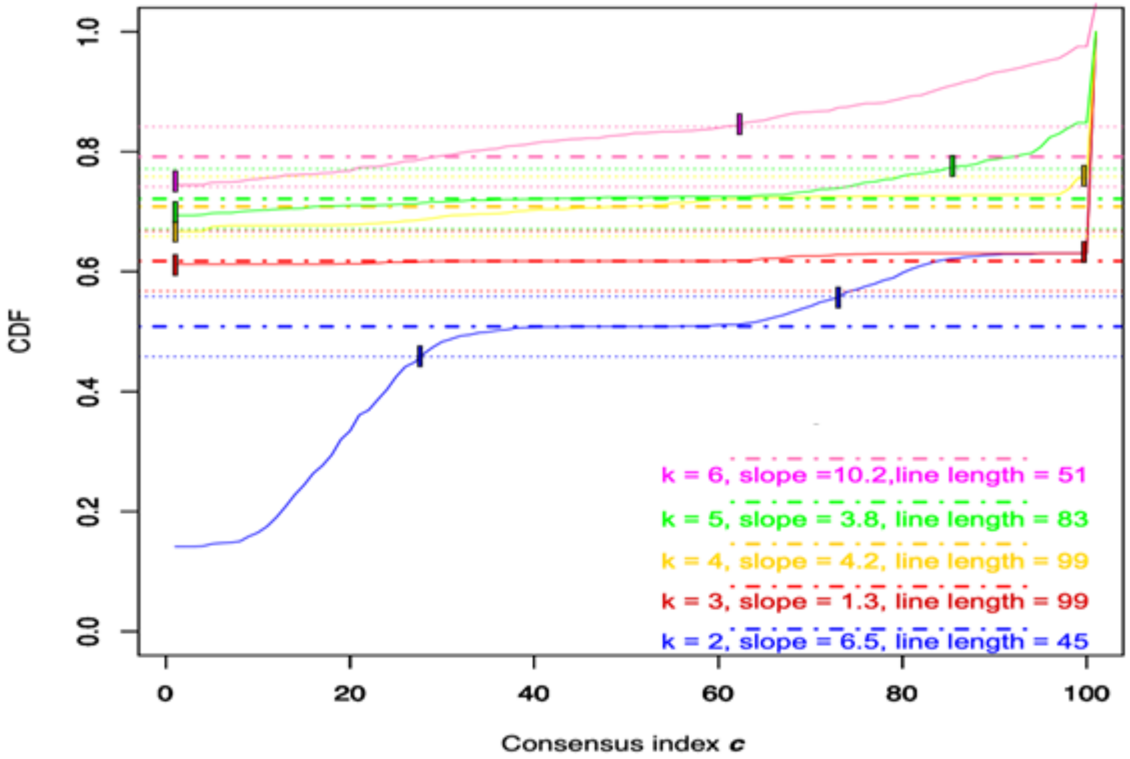

B.

Figure S2: *A. Consensus matrices for k = 2:6. B. CDF plot for optimal k selection. The dot-dash line shows the maximum possible CDF value for each k.*

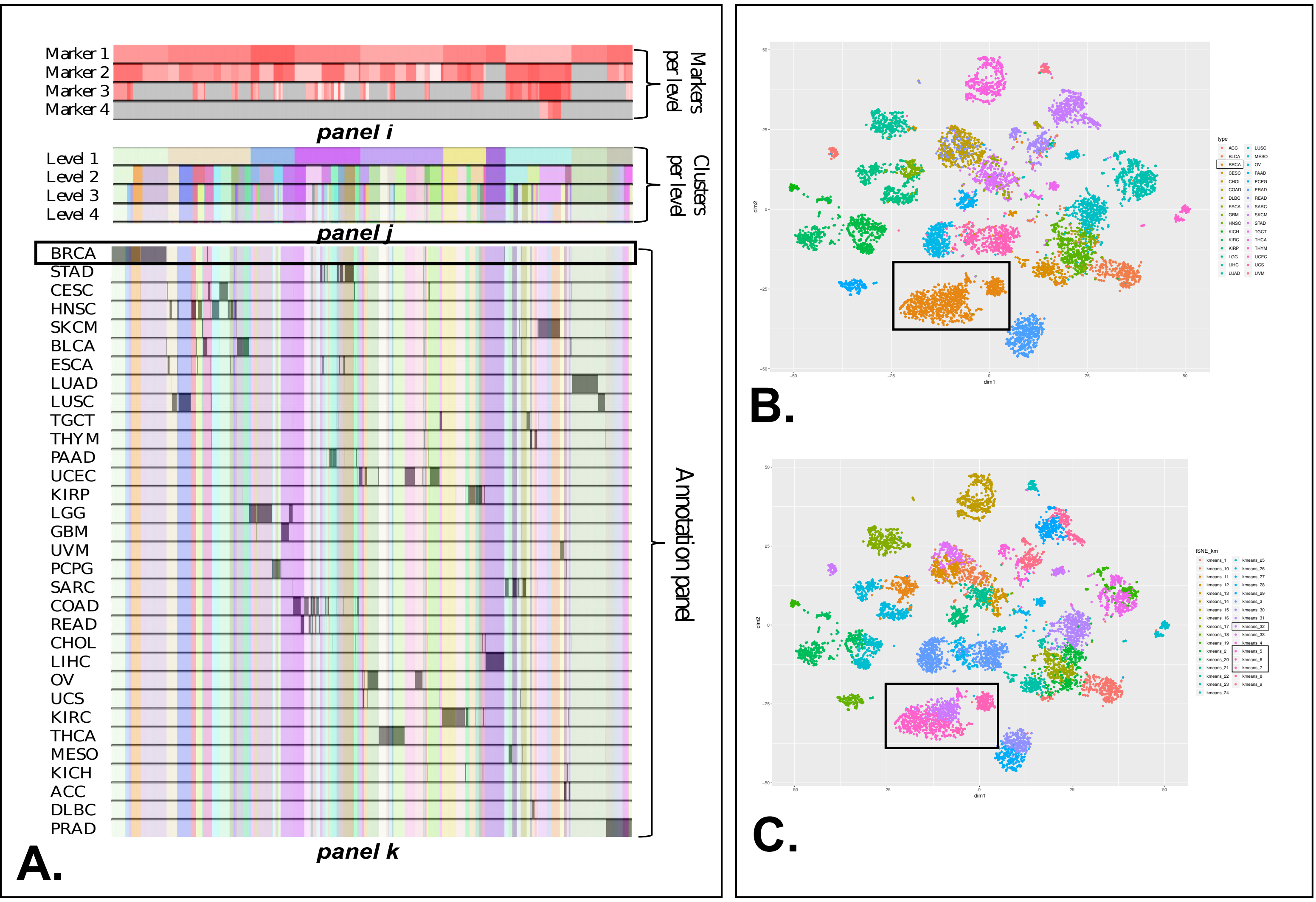

Figure S3: *Cluster visualization of TCGA pan-cancer data. A) Tracking plot showing clustering information for TCGA pan-cancer data using the RCC cluster assignment along with the cluster distribution. Breast cancer samples (BRCA) are highlighted with black box and it can be seen they divide in four major clusters. B) tSNE + k-means plot using tSNE + k-means cluster assignment and data points colored by cancer type. BRCA samples are highlighted with black box. C) tSNE + k-means plot using tSNE + k-means cluster assignment and data points colored by tSNE + k-means cluster assignments. BRCA samples are highlighted with black box and it can be seen they divide into multiple clusters. The major clusters are highlighted in the legend. Using tSNE + k-means plots B and C, it is difficult to visualize or interpret the clustered data whereas RCC provides an easy and visually convenient alternative of tracking plot A for the same.*

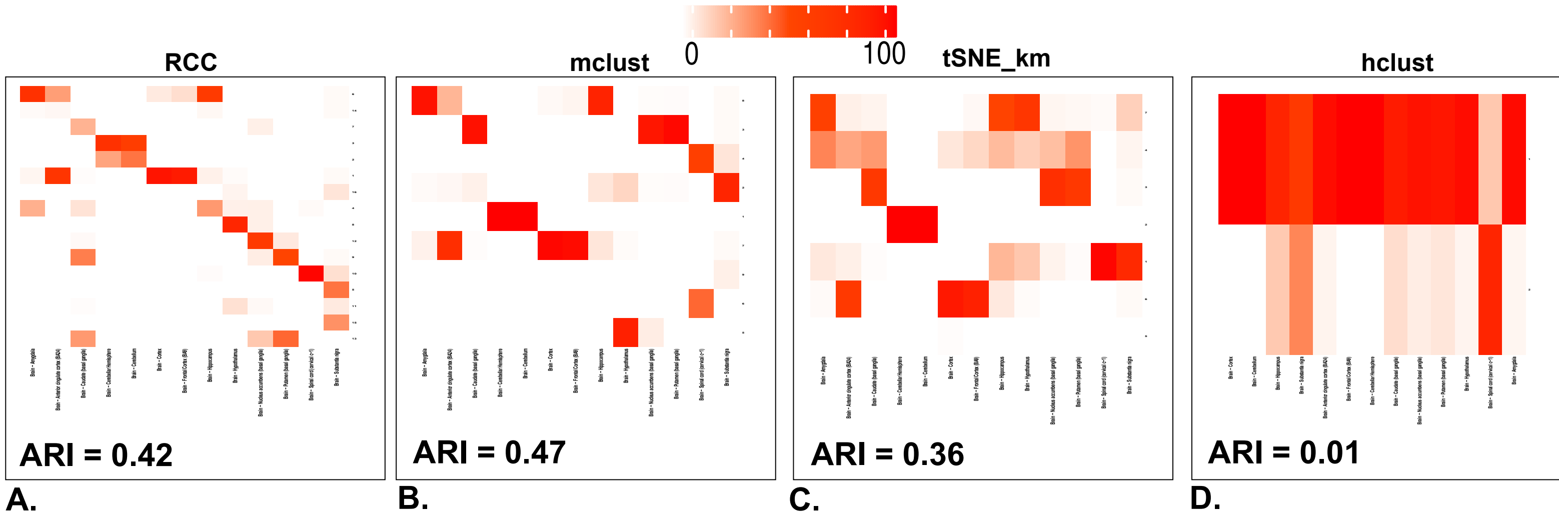

Figure S4: *Cluster annotation plot of brain samples from human tissue dataset. Except for mclust, all the algorithms used the entire human tissue data for clustering where optK of 53 was provided for tSNE + k-means and hclust algorithms. Mclust doesn't work for the full dataset, hence we took the subset of brain samples and ran mclust separately on them. The brain samples were divided into 16 clusters by RCC, seven clusters by tSNE + k-means, two clusters by hclust and nine clusters by mclust. Based on the cluster annotation plots and ARI, RCC does a better job at dividing the brain samples at sub tissue levels.*

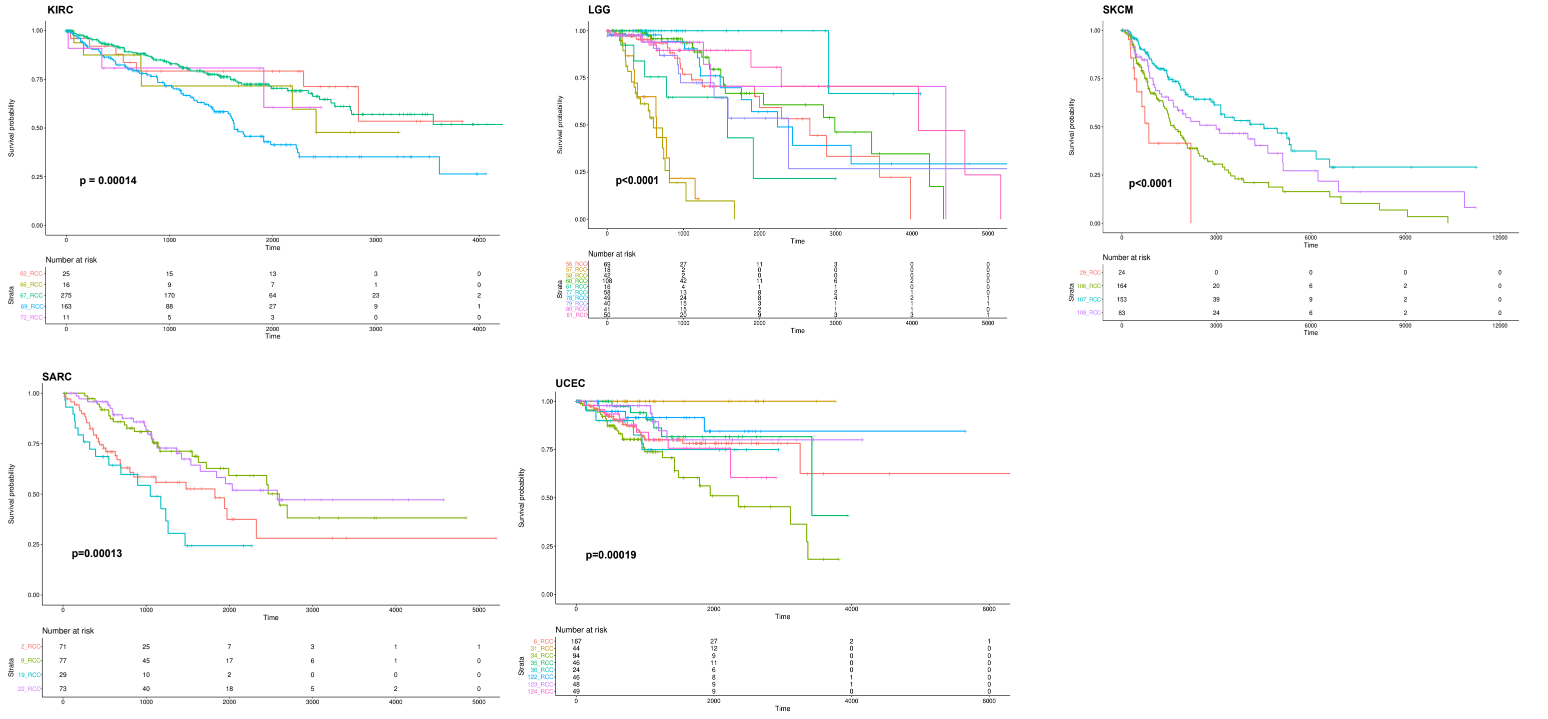

Figure S5: *Survival plots for KIRC, LGG, SARC, SKCM, and UCEC cancer types.RCC divided these cancers with significantly distinct survival profiles with p < 0.01 consistently across 10 runs.*

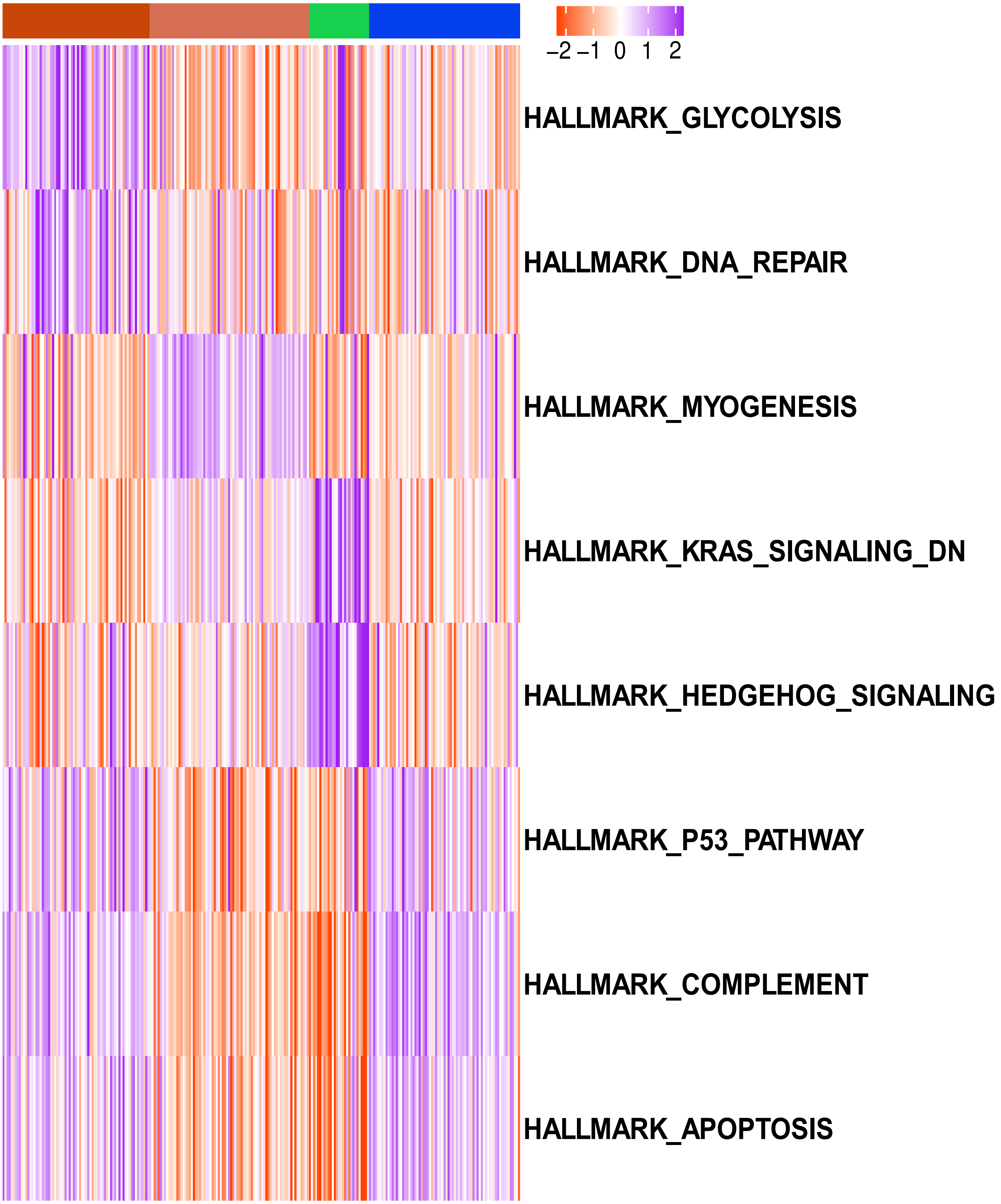

Figure S6: *SSGSEA analysis of SARC clusters. Cluster one shows enrichment in glycolysis and DNA repair gene sets, cluster two shows enrichment in myogenesis geneset, cluster three shows enrichment of genes downregulated in KRAS pathway and genes involved in Hedgehog signaling whereas cluster four shows enrichment in complement pathway and apoptosis gene sets. The gene sets were downloaded from Molecular Signatures Database (MSigDB)*

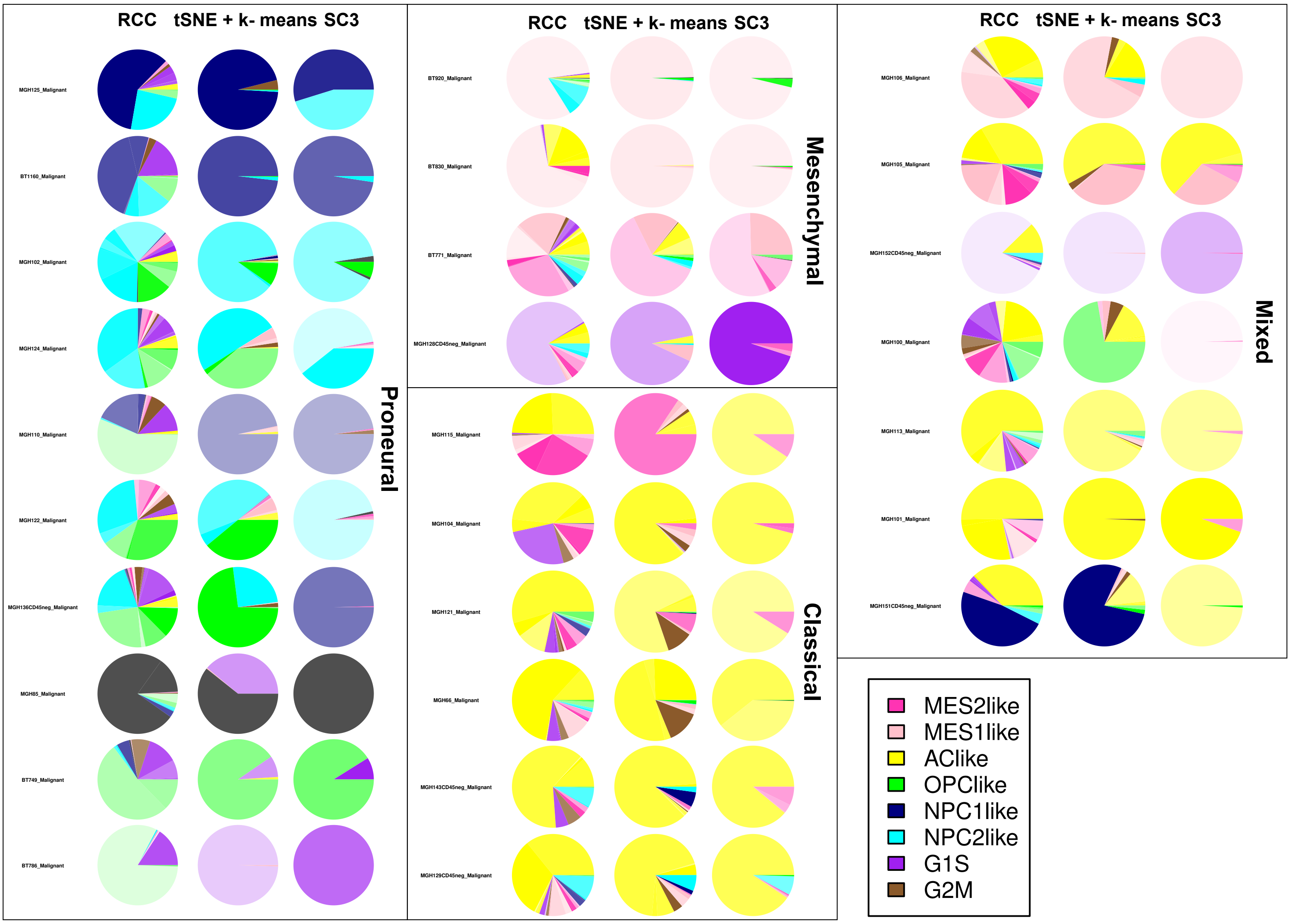

Figure S7: *Pie Charts showing cellular states across 28 tumors for Neftel dataset[ref]. RCC clusters cells based on their cellular states (MES1like, MES2like, AClike, OPClike, NPC1like, NPC2like, G1S, G2M) across all 28 tumors which are not replicated well by tSNE + k-means and SC3.*

Single-cell equal distirbution simulated data

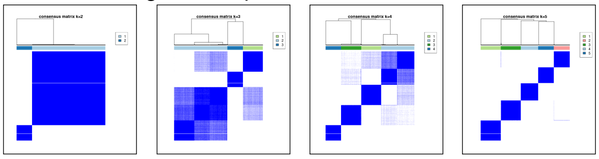

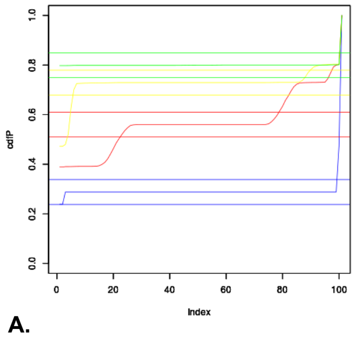

k	Line Slope	Line Length	weight	Intra cluster Stability	Inter cluster Overlap	Differential Genes
2	0.0059261529	98	2	0.997490445	0.002111673	0
3	0.0287025452	55	2	0.9473145987	0.0346224999	0
4	0.0104547335	83	2	0.9823840815	0.0078368782	0
5	0.0027686889	99	2	1	0	0

A.

Single-cell unequal distirbution simulated data

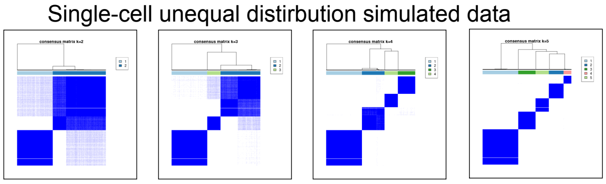

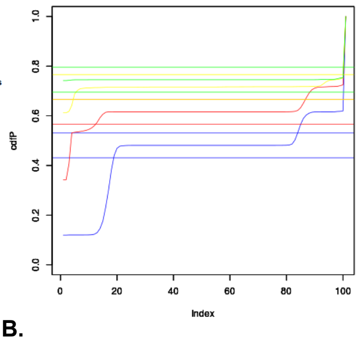

k	Line Slope	Line Length	weight	Intra cluster Stability	Inter cluster Overlap	Differential Genes
2	0.0035751401	87	2	0.9886063462	0.02968588	1
3	0.0168174221	89	2	0.9847969958	0.0078029043	1
4	0.0088536148	99	2	0.9971106649	0	4
5	0.001873813	99	2	1	0	12

B.

Figure S8: *Best k selection for simulated datasets. A) Based on the selection criteria RCC selects k = 2,4,5 as best ks for all the runs. As k = 5 has the highest frequency across all the runs and is the highest k compared to other best ks, k = 5 is selected as the optimal k which is the true k. B) Based on the selection criteria RCC selects k = 2:5 as the best ks for all the runs. As k = 5 has the highest frequency across all the runs and is the highest k compared to other best ks, k = 5 is selected as the optimal k which is the true k.*

A.

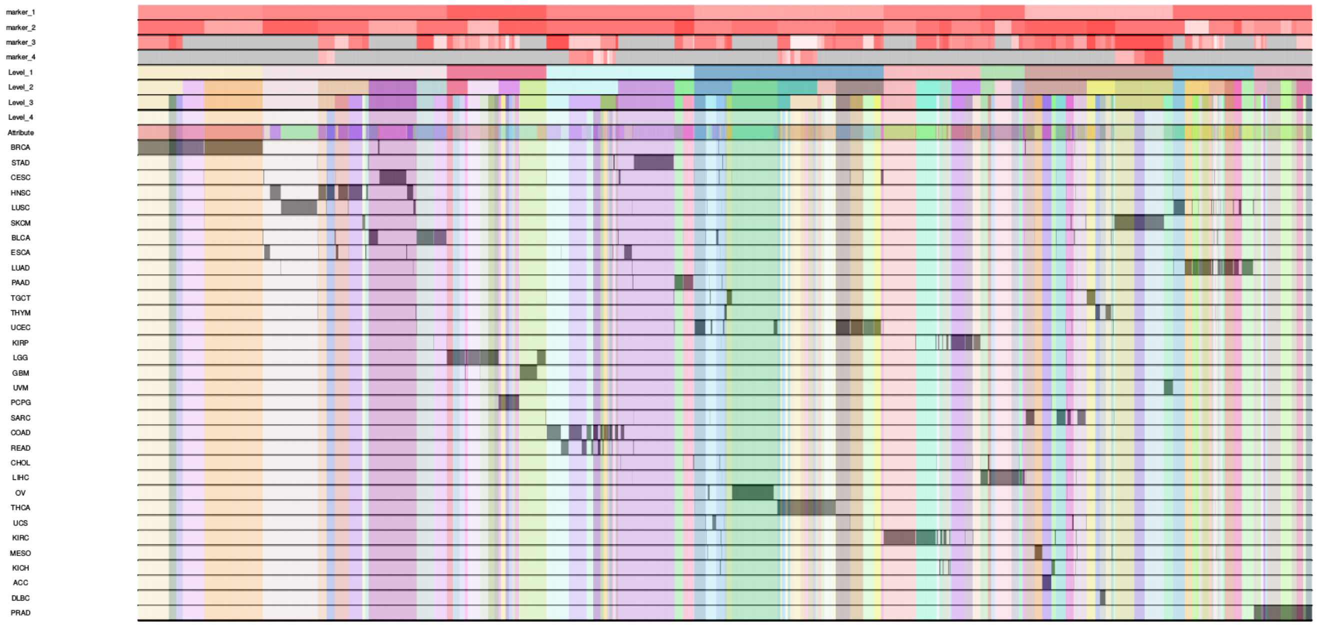

B.

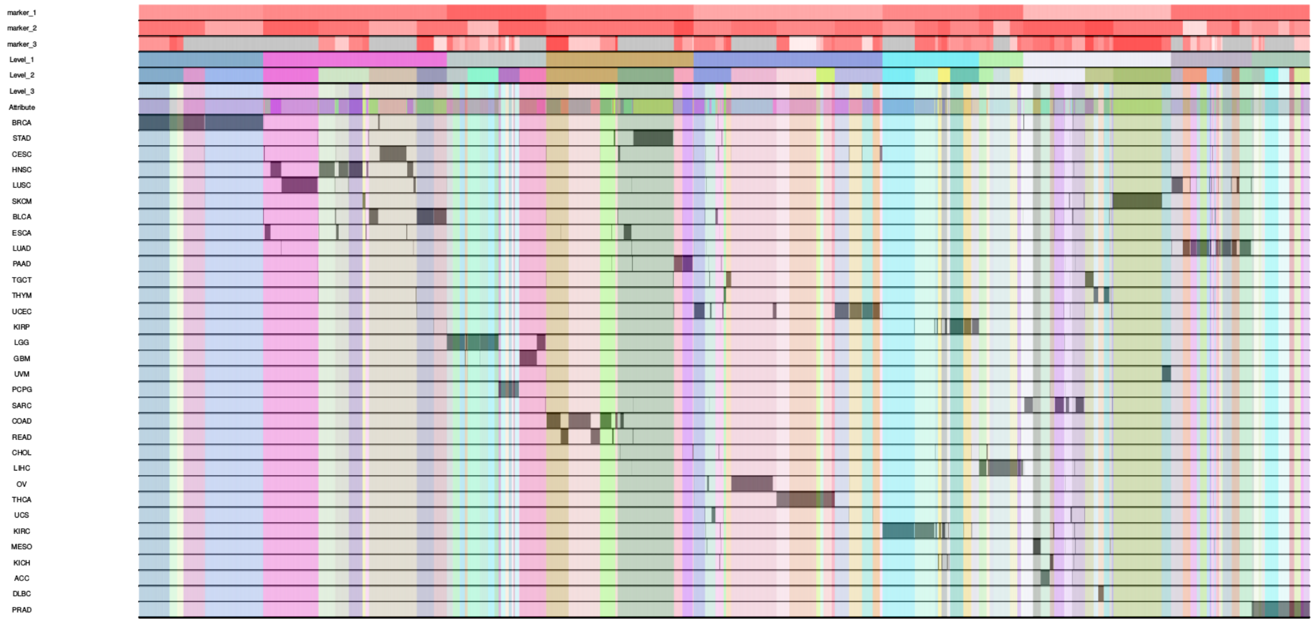

Figure S9: *Tracking plots of TCGA pan-cancer data A) Tracking plot before the level cutoff function was applied. There are 138 RCC clusters B) Tracking plot after the level cutoff function with 118 clusters.*

Table S1: Different combinations of pItems and pFeatures values across eight runs of RCC

	pItem	pFeature
CCP run 1	0.6	0.8
CCP run 2	0.7	0.8
CCP run 3	0.8	0.8
CCP run 4	0.9	0.8
CCP run 5	0.6	1.0
CCP run 6	0.7	1.0
CCP run 7	0.8	1.0
CCP run 8	0.9	1.0

Table S2: Comparison between eight parallel runs of CCP with 100 iterations each and single run of CCP with 800 iterations

	Accuracy (percentage of runs where ARI = 1)		Time/run (seconds)	
	Ivy GAP	Pollen	Ivy GAP	Pollen
RCC (n=1000)	83%	81%	54.99	57.29
8X100 (n=100)	75%	50%	295.72	310.8

Table S3: Algorithms used for benchmarking RCC

Algorithm	Function	Optimal k selection	Dataset
Gaussian Mixture Modelling for Model-Based Clustering	mclust	Determines k	Bulk transcriptomic Datasets
Hierarchical clustering	hclust	Number of clusters to Be generated is given by the user	Bulk transcriptomic Datasets
t-Distributed Stochastic Neighbor Embedding + k-means	Rtsne	Number of clusters to Be generated is given by the user	Bulk as well as single-cell transcriptomic Datasets
Single-Cell Consensus Clustering	SC3	Determines k	Single-cell transcriptomic Datasets

Table S4: Adjusted Random Index (ARI) and optimal k provided to or determined by RCC, hclust, mclust, tSNE + k-means and SC3

	ARI				optK*			
	RCC	tSNE + k-means	hclust	SC3	RCC	tSNE + k-means	hclust	SC3
Ivy GAP	0.8174	0.6991	0.6418	NA	6	4	4	NA
Human tissue	0.4532	0.6496	0.5746	NA	144	53	53	NA
TCGA pan-cancer	0.4311	0.6576	0.5433	NA	124	32	32	NA
Biase	0.9869	0.4813	NA	0.9676	6	5	NA	5
Pollen	0.8978	0.6473	NA	0.8609	13	11	NA	11
Darmanis	0.3373	0.2600	NA	0.3867	43	19	NA	25
Neftel	0.3056	0.6560	NA	0.7680	80	31	NA	60

*optK is automatically determined by the algorithm in case of RCC and SC3. For tSNE + k-means and hclust, optK is provided as user input. We chose the optk as the number of clusters **k** given in the original publication for that dataset (Table 1).

Table S5: Cluster specificity for all the datasets across all the algorithms

	RCC	hclust	mclust	tSNE + k-means	SC3
Ivy GAP	1.00	0.75	0.83	0.50	NA
Human tissue	0.73	0.54	NA	0.52	NA
TCGA pan-cancer	0.66	0.15	NA	0.59	NA
Biase	1.00	NA	NA	0.40	0.80
Pollen	0.76	NA	NA	0.72	0.81
Darmanis	0.72	NA	NA	0.73	0.68
Neftel_1*	0.62	NA	NA	0.45	0.90
Neftel_2*	0.40	NA	NA	0.06	0.23
** Neftel_1 shows cluster specificity of cluster assignment against cell types					
** Neftel_1 shows cluster specificity of cluster assignment against cellular states					

Table S6: Gene markers for Lung tissue samples from Human tissue dataset

K_1	K_2	K_3	K_4	K_5	K_6	K_7	K_8
SDR16C5	COL26A1	MUC4	PIGR	ANKRD1	CD300E	IGJ	ZBBX
HHIP	SLC40A1	ADAMTS4	CRTAC1	PNMT	RP11-373D23	WT1	C9orf117
CHI3L1	STOML3	PTX3	RASSF10	ALDH1L1-AS2	AC073410	CRTAC1	MS4A8
PTX3	C1QTNF7	IGHV3-7	C9orf117	SDR16C5	RP11-561I11	IGKV1-39	RP11-166N17
LIPG	LINC00310	ADAMTS9	HHIP	CRTAC1	KRT8P50	IGKV2-28	MMP8
CD177	MS4A8	MT2A	ALOX15B	HHIP	HIST1H3D	IGHA1	CCDC33
CCL7	TACR3	IGHV4-39	SDR16C5	PLA2G1B	IFNG	OMD	CAPSL
ROS1	LRRTM4	MT1A	C1orf158	IL1RL1	COL4A2-AS2	HHIP	KRT8P50
ALOX15B	BEND5	IGHV3-11	MFSD2A	EDN3	SNORD3C	SDR16C5	TSPAN19
CYP19A1	AC018647	CCL11	C11orf88	NCMAP	MFAP4	IGKJ4	MAP3K19
S100A12	SCN4B	RP11-373D2	EFCAB1	RP11-401P9	AL139147	EDN3	SPAG6
MFSD2A	CYS1	CD300E	MAPK15	ALOX15B	RP11-166N17	PIGR	FAM92B
FGL1	RP11-114H24	MAP3K19	MAP3K19	KCNS1	ADAMTS4	IGKJ3	RP11-373D23
METTL7B	AC004947	LIPG	AGTR2	PIGR	CYS1	ALOX15B	STOML3
CRTAC1	FAM216B	MS4A8	BMP3	ROS1	RP1-99E18	IGKV3D-15	HIST1H3D
ORM1	RP11-166N1	ZBBX	ROS1	SLC34A2	RP11-713H12	ROS1	ADGB
SAA2	DKFZP434H	SFN	STOML3	PCSK2	CCL11	IGKV3-15	C1orf158
NME1	NR1H4	FAM92B	CHI3L1	AGTR2	PRPH	IGLC6	C11orf88
ATP13A4-AS1	RP11-713H1	RNASE3	CD163	MFSD2A	IL8	PLA2G1B	AC073410
CTC-573N18	AVPR2	MUC5B	NCMAP	PRODH	RP11-114H24	IGLV1-44	C4orf22
CD163	RP11-449D8	THBS1	CCDC33	DPCR1	RP11-247I13	IGLV2-18	FAM216B
LBP	TPTE2P3	RP11-247I13	SLPI	CD163	PTGS2	IGLJ1	PLEKHS1
AGTR2	LRRN3	MUC16	FGG	SLC30A3	SH2D1A	KCNS1	CCDC60
DMBT1	RFX6	AC073410	CCDC60	BMP3	SEMA3A	PCSK2	HHLA2
CD177P1	CAPSL	PLEKHS1	SLC34A2	RP1-193H18	LIF	DPCR1	EFCAB1
ORM2	C1QTNF2	COL4A2-AS2	RP11-789C1	COL11A1	IER3	AGTR2	ANKFN1
ZDHHC19	SPAG6	SLC39A14	PLA2G1B	CSF2	C2CD4A	IGKV2-30	MUC16
SLC34A2	NLRP14	MMP8	SGPP2	MT-ND6	HIST1H4E	IGLV8-61	MAPK15
SAA1	C1orf158	RP11-325F2	FAM92B	HPN	DUSP2	IGHV3-7	BPIFB1
MMP8	AC095067	RP11-47I22	MT-ND6	AZGP1	TRAV8-2	IGHV4-34	AP000688
SGPP2	ACE	CAPSL	KCNS1	CYP4B1	CYTIP	IGKV2D-40	RP11-409C19
RP11-153M7	LINC00908	CCDC33	MT-TE	RP11-789C1	AC096579	MFSD2A	PRPH
CSF2	OLFML2A	RP11-165P7	IGHV3-7	DUOX1	PRRX1	BMP3	ARX
PIGR	KANK4	ADGB	C4orf22	CHI3L1	TBC1D27	IGKJ5	C1orf110
FGG	SGCG	RP11-170L3	SPAG6	SFTA3		IGHV3-11	SIX1
FGA	RP11-295M1	HAPLN3	CAPSL	SLPI		SLC34A2	SCGB2A1
CHI3L2	TAS1R1	BPIFB1	PRODH	MT-TE		IGKV1-5	MIR205HG
DEFB4A	APLNR	RP11-503N1	TSPAN19	APOH		IGLV2-23	GSTA2
DGAT2	HLA-AS1	PDLIM4	CFTR	DACT2		IGLJ3	RP11-356K23
CD300E	ZBBX	STOML3	ZBBX	SGPP2		CD163	CYP24A1
KRT16P2	AC073410	TNFAIP6	DPCR1	FGG		IGKV3-11	KLHL32
HP	SLC51B	SPAG6	PLEKHS1	RASSF10		IGLV1-41	PIP
HAMP	RP11-626H1	HIST1H3D	ADGB	MGST1		MZB1	NELL2
RP11-789C1	GPR158	CCDC60	IGLV1-44	MYBPHL		RP11-401P9	CCDC19
SIGLEC10	FBP2	FAM216B	FAM216B	OLAH		IGHJ4	HNF4G
MMP9	TMIE	LIF	SCTR	CFTR		IGKV1-16	LGSN
KRT16P1	CDC20P1	C1orf158	HP	FOXA2		IGHV4-59	RP5-881L22
RP11-1220K2	HCG23	AL139147	ATP13A4-AS	DGAT2		SCTR	LPAR3
RP11-184M15	EFCC1	TFPI2	IGHV4-39	IRX5		MT-ND6	C1orf168
CR1	ADGB	C9orf117	SFTA3	SCTR		IGKV4-1	HNF4A
CFTR	RP11-247I13	RP11-478C6	IGHV3-11	CLEC4E		SFTA3	TNNI3
NTSR1	KIAA0087	KRT8P50	IGHV3-21	WNT7B		IGKV3-20	MSMB
FPR1	FAM92B	RP11-214O1	CSF2	CEACAM4		CHI3L1	
KCNS1	WARS2-IT1	TSPAN19	HPN	CLDN3		IGHJ2	
LIPM	PRPH	KRT17	MS4A8	LINC00261		MT-TE	
FPR2	AC004538	SERPINE1	AZGP1	CTD-2245E15		IGHV5-51	
HPN	LEFTY2	TEX26-AS1	FAM181A	SFN		SLPI	
DPCR1	INSC	EFCAB1	ANKFN1	HOXD-AS1		NCMAP	
NCMAP	CLEC4F	RP1-99E18	IGHV3-23	RP11-311D14		IGHV3-23	
SCTR	RCOR2	IFNG	LBP	KCNJ15		DACT2	
PSAT1	CCDC33	C4orf22	PCSK2	GGT3P		FCRL5	
PYCR1	SNORD14E	SCGB2A1	MGST1	MIR142		RP11-789C1	
NFE2	LINC01031	AP000688	CLDN3	PTCSC3		FGG	
BMP3	RP11-542B1	RP1-102K2	IGKV1-16	FPR2		PRODH	
SFN	RSPO2	CCL2	IGHJ4	IRX1		CFTR	
ADM2	ITIH5	EGR3	IGHV4-61	PRR15L		IGLV2-14	
MGST1	AC016885	C11orf88	SFN	FGA		SLC30A3	
KCNJ15	RP11-95H3	RP11-166N1	FGA	RNASE3		HP	
SLPI	FAM87A	MMP10	IGKV3-11	ATP13A4-AS1		IGHV1-18	
RP11-91K9	OLFML1	C2CD4A	GSTA2	ATP6V0A4		HPN	
OLAH	TSPAN19	ANKFN1	KCNJ15	PITPNM3		IRX5	
CLEC4D	MAP3K19	RHOV	DGAT2	FAM181A		APOH	
SIGLEC9	RP11-111M2	DIO2	IGHV4-34	NKX2-1		IGHV3-15	
RP11-311D14	RP11-561I11	PIGR	MYBPHL	IRX2		IGHV3-21	
CLEC4E	AC092652	CALCA	WNT7B	TFAP2C		IGHV4-61	
STEAP4	ST8SIA6	MIR205HG	ARX			IGHV3-53	
CEACAM4	PRRG3	SIX1	HOXD-AS1			IGHV1-46	
TREML2	AGAP1-IT1	ARTN	MIR142			MYBPHL	
HMOX1	IL12A	ARX	DUOX1			IGLV6-57	
	RP11-297M9	PRPH	CTD-2245E15			IGHV3OR16-13	
	C4orf22	RP11-404P2	RP5-881L22			IGHV4-39	
	NTNG1	HHLA2	SCGB3A2			ATP13A4-AS1	
	RP11-345M2	CHST6	IGKV3D-15			SGPP2	
	RP11-713H1	HUS1B	MIR205HG			MGST1	
	EFCAB1	CNTN5	FOXA2			HOXD-AS1	
	LDLRAD4-AS	MAPK15	STK33			COL11A1	
	LRRC55	SLCO5A1	GGT3P			CSF2	
	SLITRK5	RP11-7K24	SCGB2A1			STEAP4	
	BDNF-AS	TMPRSS4	KLHL32			WNT7B	
	CCDC60	CCDC19	BPIFB1			RP11-311D14	
	TATDN2P3	TNNI3	HHLA2			PTCSC3	
	RP11-88I18	TNS4	SIX1			KCNJ15	
	LYPD6	HNF4G	CLIC6			RP1-193H18	
	CDH8	ATP12A	RP11-311D14			CD79A	
	C9orf117	B4GALNT4	CYP24A1			SFN	
	C11orf88	CYP24A1	RP6-24A23			CTD-2245E15	
	NKX3-2	BDKRB1	C1orf110			OLAH	
	S1PR5	C1orf110	IRX5			GGT3P	
	HOXA4	IL8	IGKJ3			AZGP1	
	NXPH3	L1CAM	NELL2			DUOX1	
	KRT8P50	RP11-294O2	HNF4G			FGA	
	PLEKHS1	CP	DACT2			RASSF10	
	GDF10	CXCL6	PIP			ATP6V0A4	
	REM1	KLHL32	MRAP2			VSIG4	
	ANKRD20A1	MUC5AC	FAM83A			FOXA2	
	AL009178	CLIC6	ATP6V0A4			NKX2-1	
	MTUS2	PIP	CCDC19			PITPNM3	
	TRIM45	GSTA2	MUC16			CES3	
	RP11-513N2	NELL2	TNS4			CLDN3	
	TNR	TTC23L	RP11-409C19			NKX2-1-AS1	
	RP11-373D2	RP11-519G1	LINC00908			TRIM71	
	BPIFB1	DFNB31	PRPH			FAM181A	
	HHLA2	LGSN	MSMB			PRR15L	
	MFAP4	GPR87	ARHGAP40				
	LINC00535	MSMB	LGSN				
	ZNF683	CTA-363E6	GPR87				
	PPP1R3G	RP11-409C1	RP11-356K23				
	FOXS1	VNN3	HNF4A				
	LINC00607	RP11-412H8	TRIM55				
	IFNG	SERPINB11	FER1L6				
	RP11-276H1	CCDC181	SERPINB11				
	RP11-561I11	RP11-64D22	ALDH3A1				
	GADL1	MFAP4	C14orf39				
	MTTP	HNF4A	C1orf168				
	CYP4F12	LPAR3	RAB3B				
	CCL11	SERPINB3	STRA6				
	MIR205HG	RP11-356K2	CES3				
	TET1	RP5-881L22	PITPNM3				
	RGS11	STRA6	HS6ST2				
	HOXB-AS3	FAM83A	BPIFA2				
	TMEM35	RP3-467K16	TFAP2C				
	RP11-295M1	FCRL5	PVRL4				
	VWC2						
	COL4A6						
	KLHL32						
	FAT3						
	GSTA2						
	RP11-409C19						
	NREP						
	ADIRF						
	RP11-521D12						
	FMO6P						
	ZNF704						
	CAV3						
	CTC-203F4						
	XKR4						
	SH2D1A						
	MUC16						
	NELL2						
	RP11-532F6						
	ACTN2						
	C1orf110						
	HNF4G						
	LINC00648						
	LPAR3						
	RASSF10						
	LGR5						
	RP11-481H12						
	MAPK15						
	C6orf123						
	ANKFN1						
	RP5-978I12						
	CTD-2554C21						
	SIX1						
	CYP24A1						
	PCDH11X						
	EDN3						
	ARX						
	MSMB						
	LINC00327						
	DUSP2						
	RP11-356K23						
	CD207						
	PGM5						
	RP6-24A23						
	PDZRN4						
	GPR112						
	TBC1D27						
	CSPG4P13						
	PIP						
	RSPO1						
	L1CAM						
	MYOZ1						
	C1orf168						
	OMD						
	RP11-561C5						
	DIO2						
	SPHKAP						
	AC0050774						

Table S7: Gene markers for SKCM clusters from TCGA pan-cancer dataset

K_1	K_2	K_3	K_4
KRT5	FAM69C	JCHAIN	MMP16
SERPINB5	NMRK2	IGHV2-5	GRIA2
KRT14	ABCB5	FCRL4	RNF175
PKP1	GPR143	TNFRSF17	COL9A3
TRIM29	OCA2	TRBV10-3	ESM1
DSG1	TYRP1	SHISA3	C1QTNF3
DSC3	TRPM1	FCRL5	EDN3
COL17A1	TSPAN10	IGHV3-48	SYTL5
GJB6	GMPR	SPIC	LINC00189
DSP	PMEL	POU2AF1	LINC01579
S100A14	TFAP2A	BEND4	HNF4G
CLCA2	MGAT5B	IGKV6-21	LOXL4
KRT6A	MME	PLA2G2D	PPP1R1C
CALML3	SEMA6A	LY9	CLGN
KRT16	TRIM63	FCRL2	PPP1R9A
DSG3	CA14	LAX1	ZEB1
SFN	ABCC2	CD79A	GDNF
ADGRF4	LURAP1L-AS1	CR2	HAPLN1
EVPL	SLC6A17	FAM30A	SCRG1
CERS3	C1QL4	FCRL1	ANGPT1
FAM83C	KCNH1	TNFRSF13B	FGF2
RAB25	LINC00518	LINC02397	DNAH9
GJB2	PNMA6A	PNOC	ANO3
CWH43	ST8SIA6-AS1	TRBV19	AGMO
PKP3	LINC01531	IGHV1-58	LIFR
SDR16C5	MLANA	CD19	MIR100HG
KRT6B	LINC01317	HSH2D	CYP26A1
GPR87	TFAP2A-AS1	IGKV2-30	ST8SIA5
KRTDAP	SLC5A10	MS4A1	SEMA3A
SBSN	TYR	ITK	CMTM5
SH3RF2	SLC45A2	LINC01781	ANKRD45
ANXA8	IRF4	IGKV4-1	ITGB3
SPRR1B	TMPRSS13	IGKV3OR2-268	FREM2
S100A7	LINC01320	IGKJ5	GAP43
BBOX1	RASGRF1	P2RY10	TMEM178B
KRT6C	MLPH	TRBV29-1	SYT1
ZNF750		TMEM156	ABCA8
ANXA8L1		NUGGC	MAGEA10
KRT17		FCER2	HOXD13
IVL		IGHV4-34	LOXL3
MIR205HG		ICAM3	B3GALT1
FGFBP1		IGHM	CSMD3
PLA2G4F		TRBV7-9	GAPDHP14
GJB3		CD27	MEOX2
DUOXA1		AMPD1	PTPRZ1
TNS4		IGHV3-15	PTN
CASP14		CR1	PLCB4
KLK11		IGHV3-49	PDE1C
DSC1		IGHV3-30	SERPINA5
LY6D		MIR4539	DNAH2
CALML5		MZB1	SLC5A3
PPP2R2C		IGLV3-19	GPC4
KLK7		VPREB3	FREM1
TMEM40		IGHV3-23	AARD
FERMT1		TRAV12-1	F5
ALDH3B2		RHOH	IL13RA2
ABCA12		IGKV3-15	SERPINA3
GJB5		P2RX5	DLX1
POF1B		IGHV1-18	SORL1
PLA2G4E		IGLV7-43	SNX22
SERPINB13		C7	SOX2-OT
MUC15		IGLV1-40	ZFPM2-AS1
CAPNS2		IGKV1-16	LAMA4
TP63		TRBV5-1	MEOX2-AS1
SLC6A11		SLAMF6	C4orf19
CBLC		SP140	DLX2
KLK5		MIR8071-2	TRIM9
AP1M2		IGHV3-33	MAP9
PPL		CD38	ITIH6
PDZK1IP1		TRAV21	ADRB1
PROM2		MIR4538	GAL3ST1
FAM83B		IKZF3	MAGEA1
TTC22		IGKV1-17	ERRFI1
FOXN1		CLEC17A	NID1
FAM83F		IL21R	ITIH5
FAM110C		IGKV2D-30	UNC80
ESRP2		FCRL3	ABCA6
CD24		TNFSF11	PCDH9
OVOL1		IGLV2-11	ART3
C2orf54		GPR174	SLC30A8
KLK8		TRAC	PCSK1
PRSS8		BANK1	LINC00652
TMPRSS13		KCNA3	SOX11
PERP		TRAV13-2	TMEM100
WFDC5		IGHV4-39	SEMA3E
TACSTD2		TRAV12-3	SLC26A4-AS1
KRT1		STAP1	FAM135B
C1orf116		TRAV2	SPP1
GRHL2		PIK3AP1	PLPPR1
SCEL		CYTIP	TMPRSS9
DMKN		IGHV1-2	ATP1B2
FAM83A		CXCR2P1	TNFRSF19
LGALS7B		IGKV1-8	GABRA3
TMEM184A		IGLV1-47	PALM3
EHF		IGHV6-1	ATP2B1
SPINK5		IGKC	IGFBP2
BNIPL		IGLV2-14	STK32B
PGLYRP4		IGHV3-53	SPRY4
LAD1		IGKV2-24	ZFHX4-AS1
RHOV		IGLV3-1	CSPG5
BNC1		PTPRC	NPTX2
NECTIN4		IGKV3D-11	IGFBP3
INAVA		IGLV5-45	GRIK3
CDHR1		TRAV14DV4	ALDH1A3
VSNL1		IGKV3-20	CHRM3
C6orf132		IGKV3-11	CDH19
PGLYRP3		TRBV5-4	AKAP6
EPN3		SCIMP	DOCK4
IRF6		IGKV2D-40	TESC
KRT75		IGKV1-5	SFRP1
CRYBG2		TRBV3-1	CFI
NIPAL4		IGHV3-73	KCNA4
SDR9C7		SIT1	GDNF-AS1
IL22RA1		SSTR3	NEGR1
PRSS3		ANKRD36BP2	SLC26A4
KRT80		TRAV8-3	LINC02458
FAT2		CD247	ANO4
CA12		IGHV1-3	PRSS35
KRT78		TRAV29DV5	TRIL
IL36RN		TBC1D27P	ADAMTS9
KCNK1		CD5	ST6GALNAC5
SPRR1A		IGHG3	AFAP1
KLC3		ZBP1	CNTN4
KLK10		HAVCR1	GPR37
ARHGEF4		IKZF1	AQP7P1
CXCR2		IGHV4-28	TF
SDR42E1		TIMD4	HRASLS5
EPPK1		IGHV3-21	SCN9A
CHMP4C		IGHV3-11	LGI4
S100A2		TRAV3	TMEM47
AQP3		ADGRG5	NGEF
GGT6		CD3E	PRKAA2
PLA2G4D		IGHV3-7	MYBPC1
IL20RB		IGLL5	COL22A1
SPRR2E		CD3D	C3orf70
GRHL3		MIR8071-1	LINC01711
BPIFC		BTLA	FRAS1
KDF1		IGHV4-59	CHRDL1
DEFB1		TRBV6-6	CYP27C1
A2ML1		CCR9	VAT1L
BICDL2		SH2D1A	BAGE2
HES2		CD3G	JPH1
SLC39A2		TRAV9-2	DGKB
DLX3		CD79B	BMPER
NCCRP1		TRAV13-1	ITGA10
CLDN1		IGLV3-12	ADAMTS20
IL20RA		ZNF831	CLDN11
S100A8		ADAMDEC1	CLCN3P1
IRX2		IGKV1D-12	BAALC
PNPLA1		IGLV4-69	EPHA3
CSTA		TRBV2	NLGN3
DSC2		TRAT1	ELOVL2
TENM2		TRAV8-2	FOLH1
LYPD3		TESPA1	HMGA2
FGFR3		SLA2	SCN1A
DUOX1		IGLC2	SV2A
FAM84A		IGHG1	C5orf58
SLPI		SIRPG	PLCXD3
LY6G6C		RHEX	COL11A2
C19orf33		TRBC2	RGS5
GRHL1		IGHV3-13	SLC38A11
KLK13		LCK	LCTL
SCNN1B		PAX5	STK33
MUCL1		IGHV3-74	CHL1
USH1G		SAMSN1	EYA1
TRPV3		MIR4537	ZNF660
CAMSAP3		IGHV1-46	KLHDC8A
TMPRSS4		IGLV2-8	LPAR4
GSDMC		IGLV10-54	C1GALT1C1L
BARX2		IGHGP	ATP2B1-AS1
TP53AIP1		LY75	TSPEAR-AS1
CDH3		IGLV3-16	MGAT4C
LAMC2		FCAMR	ELOVL2-AS1
NRG1		IGLV2-23	PDE7B
TGM5		TRAV8-4	DZIP1
FOXE1		ABCD2	KCNIP1
SPTSSB		CD96	COL19A1
CKMT1A		LINC00861	RN7SL417P
CKMT1B		CNR2	FAM131B
SCNN1A		IGLV9-49	KCNA2
IRX4		DNAJC5B	MAGEC2
WNT7B		IGHV3-66	OTOL1
CRB3		CCR7	MEGF10
BSPRY		IGKV1-27	SLC22A17
PICSAR		CXCL13	SLC35F1
HOPX		TRAV8-6	GRIA4
SERPINB2		LINC01684	SLCO1A2
SERPINB3		IGHJ3	CNR1
CD164L2		TLR10	LINC00237
AKR1B10		ITGAL	LINC01945
C10orf99		LINC01215	SDC2
CDS1		TBX21	ZNF536
ALOX12B		IGHV5-51	DAB1
LARGE2		IGHJ3P	SEMA3D
FAM160A1		IGHV4-31	PRRX1
WNT7A		IGLV3-25	MGP
SERPINB7		HLA-DOB	TRPM3
FAM25A		IGHV3-72	CNTN6
PLA2G3		TIGIT	ADGRG6
KPRP		CD6	TAGLN3
BCL2L10		IGKV1-9	FAM198A
SULT2B1		IGKV1D-16	BACH1-IT1
SPRR2G		PLAC8	DNALI1
GRB7		IGKV5-2	DNM1P51
CYP4F22		CCR2	CSAG3
EPB41L4B		ACAP1	LRRC17
MPZL3		LINC02362	CREB5
RASAL1		LTA	STARD4-AS1
C1orf210		IGLV3-9	TMEM229A
TINCR		LILRB1	PMP2
CPA4		IGHG2	LINC00941
PTK6		CXCR3	RBMS3
CXCL14		SLAMF1	GALNT8
EPHX3		IGHJ1	RNF157-AS1
COL4A6		IGKV1-6	ADAMTS9-AS2
TPRXL		ZAP70	APCDD1L-AS1
OVOL2		TRBV11-2	MRO
RHOD		PARP15	KLHL14
SLC47A2		TRBV6-1	ZNF157
TMEM45B		LRMP	LINC00327
DIO2		UBASH3A	OLFM3
ITGB6		CD2	KCNMB4
TGM1		IFNG-AS1	SEC14L4
LCE3D		TLR8	IGFBP5
RAET1E		IGLV6-57	ATP10B
RDH12		IL2RG	GPC6
CALML3-AS1		CD52	HPN-AS1
EPHA1		IGLV7-46	CEP126
TMEM154		CXCR5	COL9A1
POU2F3		C16orf54	PLEKHB1
KRT15		TRGV10	TFAP2C
PLCH2		IGLV1-44	SLC45A1
LINC02560		TRAV12-2	FOXA3
ANKRD35		IGLV1-51	OTOGL
ALOXE3		CD180	CHL1-AS1
PCSK9		TAGAP	FAM133A
MPP7		THEMIS	PCDHB18P
PPP1R14C		IGKV1-39	FABP7
UNC93A		SIGLEC11	KIF5A
GPRIN2		TRBV28	CMTM8
TUBA4A		IGHV2-26	FAM84B
DEGS2		LINC02384	NFIA-AS2
RNF222		IGHV4-4	ARHGAP20
PLA2G2F		TRBV7-6	NCAM1
ST14		BLK	LGALS12
CDSN		IGKV1D-13	COCH
DAPL1		IPCEF1	ZNF860
CRCT1		IGKV1-12	DHH
CNFN		EXOC3L4	MIR181A1HG
WFDC12		RAB37	LINC00326
SOX15		IGKV3D-15	C2orf40
CHP2		IGHV4-61	TRHDE
S100A9		TRAV17	VCAN
RASSF10		GZMK	LIFR-AS1
SPRR2D		SELL	STK31
KRT23		TFEC	CSAG2
ACER1		IGLC3	PPP1R3C
AKR1B15		TCL1A	GABRR1
ALDH3A1		IGHV1-24	RRM2P3
HCAR2		IGHV5-78	SBSPON
MAB21L3		CYBB	C8orf31
SPRR2A		SLAMF7	SLC9A7P1
SPINT2		IGKV3D-20	LINC01482
SLC15A1		SFRP4	ADAMTS9-AS1
TMEM125		PDCD1	PDZRN4
COL7A1		CLECL1	MYH15
ZBTB7C		LINC02325	
IGFL1		TRGV2	
CRYBG1		ART4	
S100A7A		GPR18	
LCE3E		TRAF3IP3	
SLURP1		IGKV3-7	
GJA1		UGT2B17	
RBBP8NL		IGHV2-70	
HAL		LINC02446	
LGALS7		CD5L	
SOWAHB		TRBV20-1	
CST6		TRAV26-1	
ARHGEF37		SNX20	
SYT8		LTB	
ANK3		IGKV1D-8	
KLF5		LINC02416	
FOXQ1		RTP5	
ASPG		TRBV6-5	
SLC5A1		IGHV3-64	
CRABP2		GPR171	
IL1RL2		IGLV4-60	
JUP		CD8B	
HOXC13-AS		CALHM6	
PRSS22		LINC01857	
PI3		GBP5	
CLIC3		SPOCK2	
KRT16P6		CLEC6A	
GPX2		IGHV4-55	
PITPNM3		SIGLEC10	
SPRR2B		PCAT29	
ENDOU		IGHV1-69	
FUT3		IGHV3OR16-9	
MICALCL		IL2RA	
F2RL1		SPIB	
MPZL2		TRAV22	
RNASE7		RGS18	
PCP4L1		SIGLEC8	
WFDC21P		TRBV9	
EPGN		FAM129C	
LINC01343		FAM46C	
LCE1F		IGKV2D-29	
FAAH2		PIK3CG	
FGFR2		PRKCQ	
ENTPD3		IGHD	
XKRX		TRGC2	
NEBL		TBC1D10C	
TMPRSS11D		BIN2	
PAK6		TRAV4	
CD1A		TNFRSF9	
SERPINB4		CD48	
IGSF9		TRBV4-2	
BTBD11		GVINP1	
SH2D3A		CARD11	
KLK6		XCR1	
ITGB4		IGLV3-21	
SH3PXD2A-AS1		VNN2	
FADS6		TRAV19	
GSDMA		PTPN7	
FLG2		TRBV5-6	
CNTNAP2		SPN	
MYOM3		HNF4G	
AHNAK2		CD69	
C12orf56		IGLC7	
XDH		NCR3	
P2RY2		APOL3	
RAET1L		CD7	
CDC42BPG		PRKCB	
ALS2CL		CSF2RB	
CYP27C1		CCL5	
LCE1C		IGLJ1	
IL36G		CD72	
POU3F1		TRAV16	
CIDEA		GPR65	
KRT16P1		PIM2	
EGFR		ADAM28	
GPR1		IGHA1	
LOR		TRBV4-1	
RPTN		FTH1P22	
DUOX2		CD53	
KRT16P4		OR2I1P	
IGFL2		PCED1B-AS1	
CARD18		IGLV8-61	
KRT10		TRAV8-1	
ELF5		NLRP7	
SPTLC3		IGHD3-3	
HAS3		TRAV24	
LIPM		IRF8	
SYTL1		IL32	
LCN2		LINC00582	
RNF223		FCGR3A	
PITX1		GIMAP5	
ZNF185		SAMD3	
PTGS1		CD8A	
RDH16		FYB1	
AKR1C2		CXCL9	
NMU		CD40LG	
GBP6		LINC01934	
ESYT3		IDO1	
ATP2C2		IGHV3-20	
TPRG1		SASH3	
MAPK13		PYHIN1	
ASPRV1		TIFAB	
SLC26A9		ARHGAP25	
S100P		IGLV3-10	
SDC1		LAG3	
SFTPD		IGHV3OR15-7	
NKPD1		UBD	
ACPP		CD84	
C1orf68		MIXL1	
PNLIPRP3		CRTAM	
CEACAM19		S1PR4	
MAP7		ZC3H12D	
RAB27B		PLEK	
TGM3		PCED1B	
SLC24A3		P2RY8	
MIR200CHG		IGLV3-27	
RHBDL2		TRGC1	
ADGRF2		IGLV2-18	
LCE1A		IGHV1OR15-2	
ELOVL4		IGLV1-36	
GJB4		FLT3	
HS3ST6		DOCK2	
KRT42P		BHLHA15	
TNFAIP8L3		ICOS	
ANO9		VAV1	
PSAPL1		TRBV27	
CALB2		SIGLEC7	
CLDN4		SLC8A1	
SGPP2		LINC02422	
LCE2C		PTPN22	
CD207		TRAV25	
SOX21-AS1		XCL2	
KRT17P3		LINC00402	
SOX21		INPP5D	
SLC22A3		IL2RB	
LYPD6B		PLCL2	
SLC6A2		P2RX1	
CYP4F12		DTHD1	
NECTIN1		TRAV26-2	
TFCP2L1		RASAL3	
PLEKHN1		CCL19	
GDPD2		P2RY13	
KRT16P3		LINC01146	
KRT2		CYSLTR1	
FAM83A-AS1		RCSD1	
CNKSR1		CD28	
HEPHL1		TRAV27	
LINC01527		ARHGAP9	
SLC28A3		MEI1	
C6orf141		CORO1A	
CDA		SHISAL2A	
CEACAM6		TRAV41	
WDR66		NCF1B	
SLC6A14		SLFN12L	
SCNN1G		IL12RB1	
MARVELD3		WDFY4	
SPINT1-AS1		CD226	
EXPH5		CARMIL2	
NDRG4		TTC24	
TMEM30B		JAKMIP1	
LINC00302		IGKV6D-21	
LINC02437		KLRD1	
GCOM1		NKG7	
LCE2D		IL7R	
RNF225		AOAH	
AADACL2		GZMA	
WNT10A		EVI2B	
FLG		IGLJ2	
ARG1		AICDA	
RAET1G		FASLG	
LCE1B		AKAP5	
PADI1		SLC12A3	
CEACAM5		MIR5195	
MYZAP		TRAV23DV6	
KRT16P5		IGHV3-71	
ARHGAP40		LINC01871	
SPINT1		IGHV3OR16-8	
LAMA3		NCKAP1L	
KCNK7		CXCR6	
PRODH		LINC01484	
EPS8L2		TRAV35	
LCE2B		TRBV30	
OR7E62P		USP30-AS1	
ELMO3		TOMM20P2	
CYSRT1		TRBJ2-7	
FAM83G		TLR7	
RNF39		LINC00996	
LCE2A		CD37	
LINC01806		SIRPG-AS1	
CYP4F3		IGKV2-29	
INA		SUSD3	
PLEKHG6		LILRA4	
KRT34		IGHV3OR16-6	
CYP2C18		CD80	
EPHB6		IGLC6	
GALNT6		PRF1	
XG		IGHD3-9	
EREG		PTPN6	
FCGBP		TRBV5-5	
THEM5		EAF2	
TNNT2		TRAV36DV7	
SLC16A9		LINC01501	
GNA15		IGKV2OR22-4	
LIPK		HSF5	
MARVELD2		SLA	
OTOP3		IGHV3-62	
C5orf38		LINC00426	
CNGB1		ITGB7	
CDH1		IGHV3-22	
NTF4		HK3	
TMEM45A		IGKV1D-43	
SERPINA12		APBB1IP	
ELOVL7		IFNG	
VIPR1		CXorf21	
LCE6A		IGHV1-67	
DENND2C		TRAV1-2	
LIPN		IGLV5-48	
PPP1R13L		IGHV3-43	
FZD10		GZMH	
MPIG6B		ADGRE1	
WNT3A		MYO1G	
IL1RN		IGKV1D-17	
LINC02159		HTRA4	
ACKR2		HSD11B1	
RAPGEFL1		GIMAP4	
ACP7		IGKV1OR2-108	
NHLH2		IGHG4	
IL36B		GZMB	
ATP13A4		HLA-DOA	
KCNK6		LILRB4	
IGFL3		NLRC3	
KCNK10		NCF1	
FUT2		SEPT1	
ELF3		GBP4	
FGF11		IGKV2-28	
WNT4		CCR4	
KLRG2		CXCR4	
TTC9		TRAV39	
SPRR4		SKAP1	
GLB1L3		IGHV3-60	
PSORS1C2		POU2F2	
SP6		GIMAP7	
KRT77		LINC01281	
SDCBP2		HCLS1	
STON2		IGHV3-52	
OR7E91P		PRAMENP	
FETUB		IGHV3OR16-13	
PRSS27		TRAV38-1	
CDCP1		LCP1	
NGEF		ZNF80	
TYRP1		IRF1	
SERPINB12		IGHJ2	
EPS8L1		PSTPIP1	
CXADR		FGL2	
RBP1		NAPSB	
C6orf15		IGKV2D-24	
ESRP1		LINC02273	
SLC1A6		KLHDC7B	
MASP1		CCR5	
USP43		SOWAHD	
EGLN3		TRAV20	
ATP12A		IGHV1-45	
ARHGEF5		CD74	
S100A12		TNFSF8	
LYPD5		TRBV21-1	
CASZ1		IGHA2	
NOD2		ARHGAP30	
TRIM7		IL21-AS1	
IL18		TRAV5	
SPINK7		TSHR	
ABLIM1		LYZ	
GATA3		TRBJ2-1	
TMEM79		IGHD3-22	
LTB4R		FGD2	
NDUFA4L2		IGHV1OR15-9	
KRT17P1		STAT4	
ELMOD1		IGHV1OR21-1	
KRT16P2		CIITA	
GDA		IGKV1OR2-6	
EFS		NCF1C	
MMP28		ITGB2-AS1	
NIPAL1		RAC2	
IMPA2		HLA-DQA1	
PYDC1		CD86	
LIPG		IGHV3-63	
SLC10A6		RBP5	
FAM25BP		ANTXRLP1	
ARSF		BTK	
AKR1C1		IGKV1-33	
LCE1E		TRBJ2-3	
NEU2		EBI3	
ENTPD2		RARRES3	
ST6GALNAC1		IGKV1D-39	
KCND3		LINC01624	
PIK3C2G		VCAM1	
OTOP2		CD163	
COL4A5		CCL4	
CES4A		DERL3	
NLRP10		IL10RA	
CITED4		TOX	
RSPO1		MS4A6A	
STYK1		CLNK	
TREX2		ARRDC5	
UCA1		FCRL6	
FABP5		TREML2	
PRSS16		IGKV7-3	
LINC01214		JAML	
EDN2		IGLV1-41	
MYCL		IGKV2D-28	
LLGL2		ZBTB32	
IL1F10		CR1L	
ANXA3		TRG-AS1	
ABCG4		MNDA	
LINC01605		SRGN	
KLK9		FMNL1	
BDKRB2		BCL2L14	
HES5		TRDV1	
IQANK1		TNFSF14	
CTSV		FAM172BP	
IRX3		CLEC9A	
LTB4R2		IL18RAP	
SLC38A5		RASGRP2	
APCDD1		GTSF1L	
TFAP2C		IL21	
SPAG17		BIRC3	
KLK12		KLRB1	
B3GNT3		CD300LF	
HSD17B2		LINC01943	
PRRG4		IGHV3-41	
LCE3A		ADA2	
ADTRP		TRAV6	
DUOXA2		VSIG4	
SLC13A5		C1QC	
WWC1		GATA3	
CYP4F11		PKHD1L1	
LMTK3		CTSW	
S1PR5		TRAV10	
GPR68		CCR8	
TMPRSS11E		C4orf50	
SUSD4		SLC27A2	
PART1		WAS	
UGT1A6		TMC8	
SPTBN2		PDCD1LG2	
ARNTL2		LINC02132	
UGT1A7		CEACAM21	
CARD17		KLRK1	
ADH7		LAIR1	
MALL		IL18BP	
FRMPD1		CD244	
HCAR3		KLHL6	
LINC01133		ITM2A	
CEBPA		C1QB	
MMP3		HLA-DPA1	
FABP5P1		MS4A4A	
NELL2		IGLVI-70	
TPSAB1		IGHV3-35	
WNT5A		TRBV7-3	
F3		TRAV1-1	
DEFB4A		MAP4K1	
KCNJ15		GBP1	
RHCG		GAB3	
LPAR3		IGHV3-19	
KRT31		MPEG1	
ID1		MIAT	
C12orf54		TMEM176B	
LINC00592		GTSF1	
TUFT1		FAM92B	
RIMBP2		ACOXL	
FAM183A		CLEC10A	
VSIG10L		TMEM155	
SMIM5		FCGR1B	
FCER1A		TRBV7-4	
STX19		IGLV5-37	
PLA2R1		LINC00892	
LCE1D		PTCRA	
MAST4		C4B	
KLK1		HLA-DRA	
BRINP1		IGKV3D-7	
AMN		JSRP1	
LINC02562		CXCL10	
LINC02541		SLAMF8	
C5orf46		GPR160	
SMPD3		LINC01806	
LEXM		IGSF6	
PRRG2		FCGR1CP	
CPA3		GIMAP2	
GLTP		JAK3	
TRPV6		LILRA1	
ZNF662		HAVCR2	
GDPD3		GIMAP6	
CXCR1		DOCK8	
PLBD1		IL17REL	
SPNS2		RGS13	
KRT9		IGKV1-13	
BCL11A		C11orf21	
SMAGP		LINC00528	
IL37		ITGB2	
EDDM13		IGHV3-38	
KLF8		IGKV1D-27	
LYPD2		CP	
SYT12		CHRM3-AS2	
KLK14		HLA-DMB	
CRABP1		SLC2A5	
MYO5B		SELPLG	
FABP5P7		C3	
LAMB3		XCL1	
KLF4		CXCL11	
ST13P20		XXYLT1-AS2	
FBXO27		CD274	
TBX1		SIRPB2	
AREG		GNGT2	
S100A16		TMPRSS3	
PERM1		SAMD9L	
HYAL4		GIMAP1	
LINC01671		IGLV4-3	
KCNJ18		GPR31	
TJP3		SIGLEC14	
TACC2		P2RY12	
TPTEP1		CCL4L2	
SLC46A2		LSP1	
AZGP1P1		C3AR1	
MACC1		TNFSF13B	
EFNA3		IGHV1OR16-1	
KRT79		C1QA	
VWA2		HNRNPA1P21	
ASS1		PIK3CD-AS1	
WNT3		GAPT	
PRR9		TRAV30	
KRT17P6		IGLV2-34	
F11R		IGHV3-47	
CA9		HCST	
HOXA9		HLA-DQB1	
KRT17P2		LYN	
B3GNT8		CD4	
LINC00958		GBP1P1	
LINC00885		DPEP2	
SOX7		TOX2	
TMPRSS11F		KIR2DL4	
B3GNT4		ATP1A3	
PTGER3		UCP2	
STAP2		TAP1	
ACKR4		APOL1	
LGALS9C		IGKV1OR22-1	
IL20		PRDM8	
LYNX1		KLRC1	
GMPR		PSMB9	
CLCA4		GCH1	
AQP5		PTGER2	
DNAH3		LILRB2	
CAMK1D		LAPTM5	
FA2H		CCDC141	
CXCL17		TNFSF15	
KNDC1		ATP2A3	
MST1R		AKNA	
DLX5		GPR183	
DNASE1L2		CCL25	
GPRC5D-AS1		SIGLEC5	
AGR2		IGLL1	
COL23A1		IGHV7-81	
PMEL		C12orf42	
RUNDC3A		GPR82	
HTR3A		FDCSP	
ALDH2		TMEM150B	
HR		ACSL5	
HDC		TRBV10-2	
LMO1		TMIGD2	
OVOL1-AS1		NPHS1	
TWIST2		TRGV3	
TMEM61		CTSS	
CA2		FCGR1A	
IL1R2		PARVG	
CHRNA9		APOL6	
GABRE		CLEC4E	
COBL		GIMAP8	
FAM131C		TNFAIP8L2	
DGAT2		SERPINB9	
LSR		CD300A	
MGST1		AIF1	
STARD5		IL4I1	
FRG2HP		RAB39B	
C8orf74		DUSP26	
OCIAD2		SNAP91	
GSTA3		CLEC12A	
ACE2		TMPRSS13	
RHBG		PATL2	
PSG4		CCL8	
SLC6A9		IGKV1D-42	
SCN3B		EOMES	
TSTD1		SUCNR1	
AATBC		IGLV1-50	
FAM83E		HNRNPA1P70	
MISP		CTLA4	
GPX3		GPR141	
LINC00640		HLA-DPB2	
ARHGAP8		E2F3P1	
EYA2		PPP1R16B	
MMP10		IGHV1-12	
SLC2A1		PACSIN1	
FAM83H		KIAA1324	
IL1RL1		TRGV4	
PDPN		SLC7A7	
TMC5		OTOF	
PLEKHG5		HAMP	
SLC18A3		IGKV2-26	
OLFM2		CYTH4	
RIPOR3		DOK2	
MLIP-IT1		MFNG	
FAM3D		IGHV1-14	
BMP7		C2	
TPSB2		CCL3	
KIAA1522		CD200R1	
PIP5KL1		IDO2	
ANKEF1		OCA2	
CYP4B1		EPSTI1	
ARL4D		SERPINA1	
AZGP1		SIDT1	
SPRR2C		TRGV7	
CNGA1		CCRL2	
PSORS1C1		SIGLEC1	
ANO7L1		ACY3	
THBD		LINC00494	
ZC3H12A		IGKV1OR22-5	
MDFI		GFI1	
FAM167A		DOK3	
SPRR3		CCR6	
AKR1C3		MYO1F	
PLA2G4E-AS1		GPA33	
ALOX15B		RUFY4	
OR7E14P		ADH1B	
CRNN		GP1BA	
ARTN		RASSF4	
FUT1		HVCN1	
OCA2		KIR3DX1	
NUP62CL		HCK	
ANKK1		FPR2	
TRPM1		GPR132	
MIR31HG		WDR86-AS1	
RORC		HLA-DQB1-AS1	
CCDC3		ITGAD	
IFFO2		PTGER4	
IRX1		TNFRSF1B	
GPT2		IGLV2-5	
LINC01206		FLI1	
ADCY2		TOGARAM2	
LRRC8E		MIR155HG	
PGBD5		PTGES3P2	
LAMB4		RNASE6	
GRAMD2A		ALOX5	
ZNF488		LINC01055	
PAQR5		ABCB5	
ADGRF1		TRPM1	
TFAP2B		TNFRSF13C	
CELSR1		HLA-DMA	
WISP3		ACOD1	
FAM46B		SH2D1B	
NFE2		SLC15A3	
IGFL2-AS1		HLA-DRB6	
IL17RE		CASS4	
SPRR2F		LY86	
SULT1E1		MDS2	
LGALS9B		CASP5	
EVPLL		CCL3L3	
NPR3		WARS	
CYP2S1		CLIC2	
KCTD4		MAL	
TUBAL3		NFAM1	
SERINC2		SPI1	
SLC38A4		SLCO2B1	
NMNAT3		CD40	
GLTPD2		FCN1	
LRG1		RPL4P1	
CYP2E1		C1orf162	
PGLYRP2		RSPO3	
LCE5A		PKD2L1	
DQX1		ARHGDIB	
SPINK6		MMP12	
KRT3		C19orf38	
ERVMER34-1		APOBEC3D	
GCNT4		GGTA1P	
GPR39		C1orf228	
WNT11		CLEC4C	
RIMS3		CLU	
PKP2		PLD4	
PAX1		KIF21B	
EDAR		IGHV3OR16-15	
LINC01503		LAT2	
KRT13		PIK3R5	
TRIM15		IGHV1-17	
MN1		LINC00926	
SLC22A23		ABI3	
LINC00707		TRIM22	
ADGRD2		SIRPD	
GSTO2		CD300C	
MMP27		IGLV2-28	
TFF3		ARHGAP15	
MMP1		SPATC1	
NPBWR1		RASGRF1	
GLIS1		PMEL	
B4GALNT2		CSF1R	
CDH26		NLRP6	
SH2D4A		STAT1	
TEX101		IFI30	
FTLP14		IGHV3-76	
LINC02178		DBH-AS1	
PLEK2		BHLHE40-AS1	
HPSE		TYROBP	
NRARP		LRRC25	
LINC01750		NMRK2	
CLEC2A		NCR1	
DOC2B		CMKLR1	
IFNWP19		SIGLEC9	
SOSTDC1		IGKV1D-33	
PRRX2		TSPAN10	
TP73		IGKV1OR2-3	
HSPB8		P2RY6	
SDK2		GRIN3A	
NBEAL2		IGLC5	
OCLN		PLA2G7	
SHANK2		IGKV1OR2-11	
MMP7		ITGAM	
SLC5A10		TRBV24-1	
LINC02323		IL27	
EN1		APOBEC3H	
DACT2		PDE6G	
PALMD		BFSP2	
CLDN17		ABI3BP	
COL6A5		IFI44L	
IL1A		IL12B	
UNC5B-AS1		TMEM130	
STARD10		COL4A4	
CLTB		FPR3	
F12		IGHV3OR16-11	
SLC52A1		TRBV23-1	
ABCC2		RIPOR2	
UPK1B		IGHV7-56	
SEMA4B		TRGV5	
LINC01317		P2RY14	
TRIM16		DPEP1	
KCTD11		FCER1G	
FXYD3		DBH	
C16orf74		GPR25	
C11orf87		MARCO	
PLEKHA7		NAT8B	
MAL		AIM2	
MKRN2OS		SEPT6	
MEOX1		LINC00158	
PTPRF		XIRP1	
VDR		LINC01358	
ST8SIA2		CHI3L1	
MSX2		ACSL6	
BDKRB1		PILRA	
VGLL1		IGHV7-27	
FOLR3		IL33	
SLC19A3		TRBV11-1	
HRH3		TFF3	
APELA		CHST4	
CYP4F2		ERMN	
FABP5P11		FAM177B	
ARHGEF34P		UTS2	
ARL14		FAM107B	
MIR193BHG		CMAHP	
KIAA1217		IRF4	
GABRA4		GZMM	
HNRNPA1P27		GMPR	
TMEM54		FAM163B	
RAB3D		SLC5A10	
LTBP1		CSF3R	
TBC1D2		KCNK13	
CASP1P2		LGALS9	
B4GALNT3		FFAR4	
AJUBA		FBP1	
HSD11B2		CACNA1I	
SLC2A9		C5orf56	
CTSG		GRAP2	
VNN3		PTGDR	
FABP5P2		PIK3R6	
CYP8B1		IGHV3-25	
SH3GL3		ABCC3	
SLC16A11		HEATR9	
C10orf55		LILRA5	
IRX6		IGHV4OR15-8	
HRNR		ABCB1	
MEGF6		LINC02245	
CRISP3		XAF1	
ZG16B		ADRA2A	
OTUB2		LCP2	
ZFP42		NME8	
SLC7A4		ADORA2A	
KLHDC7B		SPINT2	
MAP3K9		KLHL14	
LINC01836		TRGV9	
OR7E108P		IGKV1OR10-1	
KRT84		GHRL	
EPCAM		WNT1	
NLRX1		LINC00299	
TPBG		RSAD2	
HSPB3		CNR1	
GRIA3		ERC2	
SCUBE2		NELL2	
NXPH4		CCL2	
SEMA3F		LINC02285	
NKX1-2		FPR1	
IL22RA2		LINC00593	
LGR6		OR52N4	
KRT19		CD14	
APOB		SHD	
FZD10-AS1		CADM3	
MMP13		IL10	
PRSS12		LILRB3	
STMN2		HLA-DRB9	
PDLIM1		ALOX15	
TM7SF2		TMEM200C	
LINC02188		RGS9	
TJP2		SMTNL1	
LINC01805		TSPOAP1-AS1	
CD177		CHRDL1	
SH2D5		COCH	
LINC01305		PLA2G2A	
WNT16		LINC01727	
ADAD2		GALNT6	
BEST2		KCNN3	
NOS1		IGHV3-42	
DOCK9-AS2		STK33	
LINP1		CRB2	
MLANA			
NWD2			
ATP6V1C2			
CA3-AS1			
TMPRSS11A			
TYR			
LCE4A			
GPR78			
LINC01554			
RORA			
CES5A			
FAM110A			
HTR7			
C15orf59			
C2CD4A			
TEPP			
KRT82			
HLF			
ABHD12B			
HOMER2			
SERPINA9			
PON3			
H2AFY2			
CSRP2			
TACR1			
HOXA10-AS			
FOSL2			
LINC00933			
GLRA4			
DGUOK-AS1			
CYP26B1			
MYOSLID			
EDN1			
CES1			
TTC39B			
SOX9			
AADAC			
OTX1			
RAB44			
ADCYAP1			
NLRP2			
ETS2			
PADI3			
STAR			
TMEM238			
FAM69C			
DLL1			
CMA1			
TSLP			
AKR1B10P1			
SLC35F3			
ACOT11			
AOC1			
TENM4			
RHEX			
ABCC3			
PTHLH			
CLCN1			
SAA1			
ZNF385A			
GIPC2			
EMX2OS			
HOOK1			
LINC00519			
ADAM33			
SLC23A1			
GLDN			
MS4A2			
ALOX15			
ANKRD65			
PPP1R3C			
VWA5A			
CA6			
S100A3			
MYO18B			

Table S8: Kaplan-Meier analysis. P value for 10 runs of RCC, tSNE + k-means, hclust and cluster asssignments by Chen et al. and Hoadley et al. for TCGA pan-cancer dataset

cancerType	RCC_1	RCC_2	RCC_3	RCC_4	RCC_5	RCC_6*	RCC_7	RCC_8	RCC_9	RCC_10	tSNE + k-means	hClust	iCluster	RNA_10
LGG	2E-16	2E-16	2E-16	2E-16	2E-16	2E-16	2E-16	2E-16	2E-16	2E-16	1E-04	NA	1E-04	3E-01
SKCM	9E-07	1E-06	2E-06	2E-06	9E-07	5E-07	6E-07	2E-07	2E-06	1E-06	5E-01	1E-04	3E-02	7E-01
KIRC	7E-07	2E-06	7E-06	8E-07	6E-05	1E-04	1E-06	7E-07	3E-06	1E-06	5E-01	5E-03	NA	3E-01
SARC	7E-04	8E-05	3E-04	5E-05	1E-04	1E-04	2E-04	4E-05	5E-05	5E-05	1E-03	2E-02	2E-03	2E-01
BRCA	5E-04	9E-04	6E-01	9E-01	2E-03	2E-04	7E-02	7E-04	1E-02	3E-04	8E-01	4E-04	9E-01	4E-01
UCEC	7E-08	2E-06	3E-06	5E-05	7E-06	2E-04	2E-04	6E-06	3E-07	2E-05	9E-01	8E-04	7E-02	2E-02
LIHC	2E-03	1E-02	2E-03	8E-04	3E-03	1E-03	NA	1E-03	6E-03	9E-03	NA	4E-01	6E-01	7E-01
BLCA	2E-02	1E-02	6E-02	4E-02	2E-02	1E-02	6E-02	2E-02	2E-02	2E-02	3E-02	9E-04	2E-01	4E-01
PAAD	8E-02	9E-02	5E-02	5E-02	5E-02	7E-02	1E-01	5E-02	5E-02	9E-02	NA	1E-01	NA	1E-01
STAD	7E-02	7E-02	6E-01	8E-01	6E-01	7E-02	9E-02	8E-01	1E-01	6E-02	6E-01	2E-01	5E-01	3E-01
GBM	NA	NA	NA	1E-01	NA	1E-01	1E-01	NA	NA	NA	NA	NA	2E-01	7E-01
LUSC	2E-02	2E-01	1E-01	2E-02	1E-01	1E-01	6E-02	2E-02	1E-01	9E-02	2E-01	1E-01	7E-01	9E-01
HNSC	3E-02	1E-05	3E-02	1E-02	7E-02	2E-01	2E-02	2E-02	1E-02	4E-02	6E-04	2E-02	8E-02	4E-01
KIRP	7E-02	5E-02	8E-02	3E-02	9E-02	2E-01	2E-02	2E-02	2E-02	3E-02	4E-01	5E-01	NA	6E-01
OV	5E-01	4E-01	5E-01	9E-02	8E-01	2E-01	NA	5E-01	6E-01	6E-01	NA	5E-01	NA	5E-01
THYM	4E-01	4E-01	4E-01	4E-01	4E-01	2E-01	2E-01	4E-01	3E-01	4E-01	NA	NA	NA	3E-01
ESCA	4E-01	4E-01	5E-01	5E-01	4E-01	4E-01	8E-01	4E-01	4E-01	3E-01	5E-01	2E-01	9E-01	4E-01
COAD	5E-01	6E-01	2E-02	5E-01	3E-02	6E-01	5E-01	3E-02	6E-01	5E-01	NA	2E-01	9E-01	6E-01
TGCT	6E-01	4E-01	6E-01	4E-01	3E-01	6E-01	4E-01	6E-01	4E-01	4E-01	3E-01	4E-01	NA	5E-01
LUAD	8E-06	6E-03	4E-04	9E-03	4E-07	7E-01	1E+00	1E-03	2E-01	3E-03	NA	1E-01	6E-03	8E-01
PCPG	8E-01	6E-01	7E-01	7E-01	7E-01	7E-01	6E-01	7E-01	8E-01	7E-01	NA	NA	NA	9E-01
READ	8E-01	NA	4E-01	4E-01	3E-01	7E-01	NA	NA	1E-02	7E-01	NA	NA	NA	5E-01
UCS	3E-01	4E-01	4E-01	2E-01	3E-01	7E-01	2E-01	4E-01	3E-01	3E-01	NA	7E-01	8E-01	NA
PRAD	8E-01	8E-01	5E-01	8E-01	8E-01	8E-01	7E-01	8E-01	7E-01	8E-01	1E+00	3E-01	NA	4E-01
THCA	7E-01	8E-01	9E-01	8E-01	2E-01	8E-01	8E-01	3E-01	8E-01	6E-02	1E+00	NA	NA	2E-01
CESC	1E+00	9E-01	9E-01	7E-02	6E-01	9E-01	6E-02	1E-01	2E-01	7E-01	5E-01	8E-01	9E-01	5E-02
ACC	NA	NA	NA	NA	NA	NA	NA	NA	NA	NA	NA	NA	NA	8E-01
CHOL	NA	NA	NA	NA	NA	NA	NA	NA	NA	NA	NA	NA	NA	NA
DLBC	NA	NA	NA	NA	NA	NA	NA	NA	NA	NA	NA	6E-01	6E-01	NA
KICH	8E-01	7E-01	8E-01	8E-01	NA	NA	8E-01	9E-01	9E-01	8E-01	NA	8E-01	NA	2E-01
MESO	NA	NA	NA	NA	NA	NA	NA	NA	NA	NA	NA	NA	5E-02	9E-01
UVM	NA	NA	NA	NA	NA	NA	NA	NA	NA	NA	NA	NA	4E-01	5E-01

*Highlighted column is the run used for all the results in main manuscript.

Table S9: Gene markers for SARC clusters from TCGA pan-cancer dataset

K_1	K_2	K_3	K_4
FGF5	PPP1R12B	WNK2	LYVE1
LINC01705	FRMD6-AS2	BCL11A	ADGRD1
WNT7B	MYH11	IGF2-AS	CLEC3B
LINC02257	MIR4506	KIF1A	FBN1
HMGA2	KCNMB1	PODXL2	SCARA5
TMEM158	HMGN2P19	B4GALNT4	LILRB5
RNU6-403P	MYOCD	RFLNA	CD248
PLAUR	LMOD1	RTN4R	TNXB
AIM2	PGM5-AS1	DLX1	ABCA9
MMP13	CARMN	BMP7	AIF1L
RFX8	PTGES3L-AARS	RAP1GAP	HADHAP2
LINC00460	CSRP1	NPTX2	PLAC9
LINC01711	PGM5	MEG9	C6orf132
LINC02154	FBXL22	EFNA2	ASPA
AK5	ASB2	HS6ST2	MEDAG
LINC01605	KLF17	ZNF556	CD209
RIN1	JPH2	FRRS1L	VEGFD
SEMA7A	MBNL1-AS1	BARX1	ABCA6
ULBP2	SBSPON	IGF2	PALM
FOSL1	ACTG2	PHF21B	CLEC4G
IBSP	PLIN4	DLK1	PLA2G2A
TENM2	FLNA	CRABP1	FBLN2
COL10A1	CHRDL2	EPHB3	ABCA8
LINC02454	CNN1	EMILIN3	RNASE1
TM4SF19	MOG	NUDT10	WNT10B
ANPEP	GRIK5	SLC29A4	IL2RA
MT2A	SLMAP	SRCIN1	XPNPEP2
OLR1	MIR1-1HG-AS1	SLC4A3	ADH1C
NALCN	MAPK4	ALX4	KRT223P
LINC00520	SORBS1	PURG	EBF3
ECM1	POPDC2	WNT5A	FOLR2
ATP6V0D2	RIMS2	DUSP9	EFHD1
TNFRSF12A	MRVI1	CNTFR	TNNT3
LINC01655	ALDH1B1	SOX11	CD300LG
ROS1	ITGB1BP2	XKR5	TFPI
EVA1A	TMOD1	SDK2	ADAMTS5
ABCA13	AKAP1	ROBO2	LMX1A
ITGA10	NPAS4	HMGA2	FGD5
TMEM52B	RAB9B	FBN3	APBB1IP
LINC01050	WDR49	MPPED1	PTGES
SERPINB7	PTGES3L	MEG3	CA4
CLEC5A	KLHL23	VANGL2	SIGLEC1
UCN2	ITPR1	C2orf70	DAB2
DCN	PRR26	NKD2	FGF16
PTPN22	IGFBPL1	LMX1B	BTNL9
SERPINE1	SERPINI2	LRRC4B	EBF2
LRRC15	ACTA2-AS1	C1QL4	HTRA3
TNFRSF9	LONRF2	DKKL1	ADH1B
PLEKHN1	CFL2	CASKIN1	DCN
IL2RA	TAGLN	COL9A3	KCNB2
DCSTAMP	RAMP1	RASL11B	TRIM50
GPR39	RASL12	MEX3A	FAM180B
IGF2BP1	LPP	DPYSL5	SIX2
MME	ITGA8	LRRN1	AQP7
SIGLEC15	MIR133A1HG	PDPN	RBP7
PLEK2	SYNPO2	GPR27	MPEG1
FHAD1	CCDC9B	PRKAA2	ECM1
XDH	IL17B	C14orf39	CFD
CATSPER1	RBPMS	PNMA3	SHISA6
LINC01583	PLN	COL26A1	AICDA
TREM1	COL4A6	DLX2	LINC01133
S100A2	PCP4	DLL3	CD36
PAGE5	NAV2-AS2	EPB41L4B	MS4A4A
CCL18	TMEM252	SOX9	ELANE
PHLDA2	SERTM2	KIF26A	ACVRL1
CEMIP	TSPAN2	KLHL35	GRK5
LINC00922	CDH3	SOX8	TRPV3
HLA-DQA1	PRUNE2	PCDH8	PDGFD
CDCP1	ACTA2	DLX6	PLTP
TRPM8	KCNH2	KANK4	KCND2
SPHK1	SMC2-AS1	TMEM98	FHDC1
LINC01615	MPP7	KLHL31	CFB
MYEOV	DGKG	NYAP1	GPR1
POPDC3	RYR3	WIF1	HPGDS
LINC01614	KCNQ4	SHANK2	BMP7
PAEP	AOC3	PLPPR3	SCN4A
MAGEC2	NIPAL4	FAM69B	ELN
RPLP0P2	MORN5	GPLD1	LINC02308
CD82	TACR2	APBA2	IL33
GJB2	PLXNB3	SHISA2	ADGRG2
SPP1	SLC47A1	AFF2	ROS1
LINC00941	TPM2	CECR7	F10
TREM2	PPP1R14A	SMIM10L2A	DKK1
LINC01929	LINC00670	FAM169A	RCVRN
VEPH1	XK	SAMD11	PLAC9P1
APCDD1L-AS1	METTL24	COL23A1	CYP4F12
LINC02201	MYL9	DDX25	SGCG
LINC00973	CCDC81	KIAA1549	MFAP5
ERMN	LINC00578	EDN3	CR1
MAGEA3	DSTN	LINGO1	HS6ST2
CCL20	ADCY5	TMEM132C	CLEC4GP1
CHRNA1	RPE65	TMEM151B	VSIR
LINC00601	FBXO32	PRMT8	MARCO
EPS15P1	STXBP5L	RFPL1S	GPX3
HMGA1	LMO1	PACRG	NTN4
PDPN	PAWR	ZBED9	MRC1
SMCO2	LINC02051	CYP26B1	HSPA12B
LINC00944	RASD2	ZNF663P	ADAMTSL4
ITGAX	ENAM	BEX2	APOL3
HLA-DQB1	FAM184A	IMPDH1P8	ANPEP
KCNN4	ZFP57	GRM4	GALNT15
STEAP3	RBM24	MRAP2	PDPN
SEC14L2	DBNDD2	KCNIP1	TNXA
GFPT2	FGF13-AS1	SNAP91	KLF4
VDR	CHIAP3	SYT13	EMILIN2
ADAM12	HSPB3	EFNB3	LUZP2
CPNE7	COLCA1	GLB1L2	F13A1
MAGEA12	PRPS2	SALL1	HAS1
SLC16A3	CKMT2	SOSTDC1	SLC1A7
BNC1	RIMS3	COL13A1	CD28
CSAG1	LINC01018	NYNRIN	FSTL1
SPATA20P1	RPL7P3	RNF212	CETP
RAB27B	CALD1	COL2A1	CD163L1
MAGEA6	AKAP6	MAP2K6	TIMD4
LINC02126	ALDH1L1	ADGRB1	GAS7
FAM225A	SPECC1	ELFN2	SVEP1
CREG2	PIFO	PDZD4	TMEM176B
MMP9	REEP1	NKD1	TGFBR3
LINC01788	C8orf46	CPAMD8	TMEM236
LINC02544	KBTBD12	ALDH5A1	GPIHBP1
MMP1	GPR21	CACNA1G	C3
AQP9	SPEG	PROM1	MS4A6A
MS4A6A	ITGA7	CCDC8	MYRFL
OSCAR	TRIM46	YBX2	RARRES1
APCDD1L	SLC51B	SLC6A11	P2RY13
MMP8	SUPT20HL2	NKX3-2	TCF21
CRYBB1	NEXMIF	TOX3	LAMA2
CLDN14	HSPB7	CDC42EP5	SDK1
SERPINB4	CAP2	ZDHHC8P1	VIT
HTR7	VIPR2	NEFH	LINC00968
ZFHX4	LINC01765	ZSCAN1	CPZ
GPR158	ITPR1-AS1	TSPAN11	LMO7DN
SLAMF9	FRY-AS1	RIMS4	ABCC8
FAP	LINC01154	BARX1-AS1	IGF2
TGFBI	TCEAL2	SP9	MEOX2
SLC11A1	ROPN1B	FBXL21	CYP4B1
SMILR	HAND2-AS1	RTL1	GFPT2
TEX29	HACD1	LARGE2	FGF18
ADAM8	MYOC	HOXC13-AS	LINC01915
KRT79	HEPH	CCND2	WNT2B
CPN2	LINC01828	CACNA2D2	STEAP4
S100A9	CHI3L2	ERVMER34-1	C4BPB
KRTAP1-5	TMEM179	ZSWIM5	PTPRZ1
DLEU7	SETP2	HOXC13	SERPING1
C6orf132	DOCK3	FAM163B	S1PR2
SBSN	ASB5	HMX1	C1QTNF9
CTHRC1	LDB3	ZIM2-AS1	HIC1
ZFHX4-AS1	RASSF3	ATP8A2	GRIK1-AS1
HK2	LINC00702	SMO	AOX1
WNT10B	IGSF9B	AJUBA	SH2D1A
MT1G	CACNA1C	IRX2	PRG4
PODNL1	TPM1	SIM2	OSR2
SSX1	ADAMTS9-AS2	OCA2	CDC42EP2
APOC1	CERS3-AS1	PCOLCE-AS1	PODN
ACP5	C3orf70	CHGB	FGF10
NKX6-1	SMTN	GPR158	DLGAP1
SERPINB3	PPFIA4	OGDHL	TMEM176A
CHN1	TSPOAP1-AS1	FAM171A2	GALNT16
AGMAT	MIR4697HG	PCLO	SH2D1B
ANXA2P2	SEL1L2	TENM2	CHL1
KCNS1	RASL11A	ZNF488	PI16
LINC00565	KLHL38	ASPG	SHE
LINC01776	CSDC2	BTBD11	LXN
HAS2	GKAP1	ERC2	CHST7
PLEKHS1	WFDC10B	KIF26B	GGTA1P
SIM1	EPPK1	COL9A1	CCL14
GALNT5	RBM38	PRAME	FABP4
HTRA3	TNFAIP8L3	DLX3	LINC01354
TNIP3	EGF	CHST8	CCL8
ITGBL1	PGR	STK33	SLC14A2-AS1
SIGLEC10	MYOM1	FAM19A5	LINC01684
IL20RB	GBP2	KIRREL2	GALNT17
FCGBP	ADAM22	HIF3A	TMEM88
LINC00547	NEURL1B	LINC01006	PTPRH
CYP19A1	SYNM	CSPG4P10	NCKAP5
LINC02081	DDO	CDH22	TMEM233
MDFI	SPATA4	OBSCN-AS1	LINC02511
CDX1	RDH5	TBX1	CRYBB1
MEOX2	SLC47A1P1	DNAH14	ABCC2
KCNK2	RYR2	FBLL1	LRRC10B
NOTUM	PBX1	TBX5	CD34
VIM	ZNF37CP	LRFN2	PLA2G5
KLHL35	GNAO1	LYPD6B	CHRDL1
REP15	CASP12	MSI1	EN1
TIMP1	REEP2	CCDC181	ACSL5
SOX11	LINC02310	PLPPR1	C10orf128
GJA1	ACTN1	LGR5	PLA2G2D
LINC02535	PRIMA1	ZIC2	KRT24
	SLC25A4	CRTAC1	GIMAP1
	SHROOM3	TMEM130	FYN
	DCAF12L2	SIX1	LINC02202
	HSD17B6	JPH4	GIMAP8
	MTUS2	RBFOX3	TNFRSF1B
	PCYT1B	LRRN2	C7
	FENDRR	COL4A6	PLPP3
	COLCA2	NOTUM	MMP27
	KIFC2	IL17RD	TNFRSF8
	CASQ2	FAM189A1	GPRC5B
	PDLIM3	EYA1	CLEC10A
	PRKAA2	KCNF1	HSPB6
	TTLL7	DLX6-AS1	C17orf58
	CASZ1	IGDCC3	SPRR2B
	FAM129A	MAGEL2	CILP
	MEF2C-AS1	ZNF667	CXCL1
	TSHB	CCND2-AS1	IL12RB2
	HS6ST3	CA10	NTRK2
	INPP5J	FAM110C	APOL4
	SOCS2	PEG3	LRRN4CL
	WFDC2	DPYSL4	LINC01960
	TSPOAP1	ITM2C	CMKLR1
	MBNL1	ABCC8	SPSB4
	SCRG1	SPTBN2	TWIST2
	CELSR2	WASF3	RPH3AL
	LINC02269	DGCR5	KCNJ9
	SLC24A3	AMER2	KRT222
	TGFB1I1	SMIM10L2B	SIGLEC11
	NFASC	STRA6	APOLD1
	KCND3	VAX2	FEZF1
	NBEAP1	LINC02036	TPPP3
	DES	SEMA6A	STK32B
	PRKAG2	B4GALT6	CCL23
	MAOB	MGAT3	KLRB1
	TRAF5	ART5	PTX3
	ADRA1A	ALOX12B	SASH1
	TACC2	PCDHA2	CD55
	TNS1	EEF1A2	LINC02384
	LINC01750	CAMKV	FCER2
	PCSK7	GPRIN2	LMX1B
	LAMA3	CRHR1	EBI3
	TPD52L1	COL11A2	S100A9
	A2M	FZD10	KLHL35
	KANK2	PHACTR3	WARS2-IT1
	MAMDC2	WNK4	FMO2
	ACTC1	NTN3	TEX26-AS1
	CRYAB	SFRP5	CD200R1
	HSPB8	DNAJC12	LINC01886
	AKAP5	AGBL4	DEFB1
	PDLIM5	EN2	SERPINB2
	SGCA	GDF10	SEMA3G
	SVIL	ADD2	PDGFRL
	SLFN13	ELAVL3	LINC01091
	ZNF774	FZD10-AS1	EPB41L3
	PRR32	CNTN2	SPRR2F
	WFDC3	TH	LMO7DN-IT1
	DACT3	HOXC12	CD38
	CACNB2	CITED1	AKR1C2
	AOC4P	AQP5	KLHDC7B
	PTCHD1	CACNA1G-AS1	TLR4
	GNAZ	LINC01305	GIMAP5
	GALNT17	LRRC4C	IGFBP6
	BTC	EBF3	LINC01235
	WFDC1	SORCS2	PGR
	HPD	NGFR	CXCL10
	MYRIP	WNT5A-AS1	ADRA1B
	C9orf3	RIMBP2	EFEMP1
	C12orf54	NKAIN4	IGF2-AS
	C11orf96	PDE9A	FEZF1-AS1
	FRMPD3	KCNH3	C17orf99
	LINC00844	PLEKHG4B	IDO1
	PALLD	SEZ6L	PAMR1
	SOD3	ZNF98	GATA6
	C8orf88	PNMA8A	AADACL2
	IRF6	FGF18	PYHIN1
	KCNK3	C5orf38	GIMAP6
	DNAJB5	TPTE2P1	KCTD8
	CDC42EP3	CDK5R2	LINC00645
	ZNF620	TNK1	TSKS
	NAV2	SLC7A10	NKAIN1
	FRY	GRIK3	LINC01028
	DRD2	HOOK1	COX4I2
	LYPLAL1-AS1	BNC1	MS4A4E
	KCNMA1	GFRA1	C4BPA
	MAP1B	GBX1	ANGPTL5
	KLHL30	INA	SMURF2
	LINC01592	FZD3	CORO2B
	CYFIP2	CILP2	METTL7B
	EOGT	ZNF385C	PON3
	CA3	NELL1	DPT
	DMD	CADM1	C2orf88
	FHL1	DMKN	THBD
	LRRC53	FGFR3	GIMAP7
	OXCT1-AS1	ZBBX	CCDC80
	BAG2	NELL2	IL20RB
	SV2A	MATN3	LINC00671
	LIMS2	IGLON5	PLPPR4
	PDZRN3-AS1	NOS1	P2RY12
	PARM1	KLF14	RAB3IL1
	DACT3-AS1	ARMC4	KCNJ16
	LHFPL1	PRDM11	PTH2R
	FAM181A-AS1	SOX6	RFPL4B
	FNDC9	BMP3	ANKRD55
	DIRAS1	PLCH1	FCRLA
	RAB23	SLC25A48	PXDNL
	NEXN	SYCP2L	P2RY8
	KLHL10	TENM4	CREB5
	HAND2	FAM183A	PLAC1
	LEFTY2	WNT10B	HMGA2
	KLHL42	HOXB13	LVRN
	SETMAR	IQCA1	AQP3
	GSTA7P	KCNQ2	ABCG1
	LINC01435	KIF12	TNFSF14
	PDE4D	CRLF1	LAMC2
	NXNL2	SLIT1	ZNF385A
	LINC01016	FLRT1	PCDH12
	PARD6B	NSG1	LINC00639
	LYNX1	ESRRG	CRACR2A
	MICAL1	MIR770	CR2
	TCEAL1	EDN2	PCOLCE2
	GREM1	BMP5	CPB1
	FNBP1	TRO	MLXIPL
	SOCS2-AS1	HOTAIR	TNFAIP2
	FAXC	TFAP2A	GAP43
	MAOA	CELF5	TGM5
	ANKRD18A	DIO3	SPRR2E
	AR	CXXC4	FFAR2
	CASQ1	SYT16	LINC01230
	GACAT2	TMEM59L	COL9A3
	TTPA	AARD	LINC02289
	DCX	CHRNA4	CD79B
	ADGRE5	CYP2J2	IGF1
	LINC01778	DOK7	FCGRT
	CSRP2	SNCAIP	TMEM184A
	HMCN2	FGD5P1	PI3
	ACVR2B-AS1	TPRXL	ABCA9-AS1
	RNF216-IT1	SEPT3	MGAT4C
	ZDHHC23	GOLGA6L9	MAP1LC3C
	PTGER3	FOXN4	SPTA1
	TCEAL6	SEPT6	FNDC1
	KANK1	LINC00887	BMP4
	KLHL30-AS1	HNF4G	CHL1-AS2
	DGKI	SEMA4G	LRG1
	MAPK10	CFAP221	IL12B
	MIR1-1HG	GABRG3	BNC1
	ADHFE1	SSPO	PCSK5
	PROM1	CACNA1D	CASP10
	KCNN2	NAP1L3	ADAM12
	MCAM	FAM222A	SOAT2
	BCO1	SEMA4F	NRROS
	DTNA	SCUBE1	FCGBP
	NKD1	LINC01105	ZFHX4
	STON1	MAPK15	HOXC5
	GAS6-AS2	SYNGR1	H19
	SLC8A1	OBSCN	ROPN1L
	GOLGA8DP	MYCN	BDKRB2
	LINC00114	LINC02037	PROCR
	RPL10P7	CLCNKA	TMPRSS11A
	CBX7	NRSN1	LRRN3
	TACC1	GFRA2	LINC01645
	FGF13	RBP4	RN7SL659P
	MAP3K20	ASIC2	KCNJ5
	TP53INP2	IRX4	CARMIL1
	CAND2	ZNF334	IL1RL1
	KLHL13	KCNS1	BCAS1
	LINC00570	CTSV	ICAM2
	DOC2GP	DIRAS2	DLX3
	NWD1	CHODL	IGDCC3
	DAND5	ZYG11A	CRHBP
	PPP1R3B	MAFA	TRIM71
	ARHGEF37	CBX2	FAM124B
	PTTG2	GAS2	KLF2
	MITF	TEKT2	HAS2
	SLC22A3	NTF4	CLCA1
	NPNT	PCDHAC1	MME
	ARHGEF26	BRSK2	GPR158
	SORT1	FGF19	NGFR
	PI15	C2CD4C	CD19
	MSRB3	BEGAIN	OMD
	ANXA13	SCX	LINC00941
	RAB3D	ENTPD8	LINC01260
	C15orf41	SARDH	LINC02381
	FLNB-AS1	TERT	OIT3
	FAM201A	SELENOV	TNRC6C-AS1
	MTTP	NPAS3	C9orf66
	FCRLA	ZNF280A	COLGALT2
	CDKL1	INAVA	DNMBP-AS1
	NECAB1	CSRNP3	VGLL2
	GOLGA2P5	FBXO16	SFRP2
	ROPN1	TLE1	CRABP2
	MGARP	SOX9-AS1	ACKR3
	PFKP	FREM2	SPATA8
	NOS1AP	ESRP1	PPARG
	PPARGC1A	IGFBP2	TEX26
	TMEM200B	NUDT11	WDR86-AS1
	KLF8	POU3F3	SPRR2A
	ABCC9	DGCR9	FDCSP
	PLXNB1	SMAD9	DIO2
	LINC01354	SULT4A1	ANO4
	PNCK	NTF3	DHRS3
	STBD1	CNNM1	SOX11
	PLXNA4	PROX1-AS1	LINC01140
	TCEAL3	AMN	PODXL2
	RGS9	NKX6-1	CPNE8
	GABBR2	POU6F2	RPS4Y1
	IL24	CFAP47	
	MICAL3	GALNT14	
	ENO2	SHANK1	
	FUT9	ZNF536	
	ALDH3A1	TMEM63C	
	SNAP25	GCGR	
	ITGA1	DIO3OS	
	KY	SHISA7	
	CRLF2	FOXB1	
	PPM1E	CLUL1	
	CD9	KIAA1210	
	ADAMTS8	PAX7	
	NXPH3	LRRC3	
	HSPB1	DIRAS1	
	SH3BGRL	UGT3A2	
	UPK3A	CRISPLD1	
	ASB16	LBX1	
	TMEM56	ZNF391	
	XKR5	TGM3	
	MRGPRF-AS1	USP43	
	ADAMTS9-AS1	NXPH4	
	CCKBR	PDZD2	
	MIR497HG	WDR87	
	HHATL-AS1	LINC01122	
	LINC01160	ZFHX4	
	TCEAL4	ZFR2	
	LDOC1	NPW	
	ARL4D	ADAMTSL2	
	THSD4	WNK3	
	PPP1R3C	ESPN	
	LIPI	EFHD1	
	PRELP	ECEL1	
	LINC01489	CAMSAP3	
	LINC01797	NAT8L	
	EBF4	MYL3	
	LINC02052	CUX2	
	MROH7-TTC4	TRAM1L1	
	PDCD6IPP1	STOX1	
	OXCT1	LBX1-AS1	
	TNNI3K	SSTR5	
	RIIAD1	KCNJ4	
	ATP2B4	LGR6	
	TMEM74	ATP6V1B1	
	RAB33A	DPY19L2P4	
	CYCSP10	PCDHAC2	
	VWC2L	SIX3	
	RHBDL3	ZIM2	
	FAM46C	FAM69C	
	TMEM35A	BMP4	
	RPS15AP38	ZDHHC11B	
	TBC1D1	TBX5-AS1	
	MYO3B	SH3GL3	
	LINC01634	PNMA6A	
	PPDPFL	WASIR2	
	DIXDC1	FSTL4	
	MPL	ZIC3	
	LINC00519	PLAG1	
	TPPP	ATP7B	
	CDH1	NUS1P2	
	PHLDB2	RSPO1	
	IL33	SFRP1	
	SLC25A23	TRIM71	
	GRIN2A	SCARA5	
	DPYSL5	PCDHA3	
	CLCNKA	NKAIN1	
	WSCD2	NTSR1	
	P2RX1	ZNF280B	
	EMILIN3	PLA2G4D	
	HAPLN2	VSTM2B	
	CELF3	PRKCZ	
	TP73	ALKAL2	
	TEAD3	EDAR	
	PDE1C	RASGEF1C	
	RERG	C2orf40	
	PDZRN3	COL25A1	
	BSPRY	CACNA1B	
	STAC2	KLHL29	
	OPTC	TET1	
	HSPB1P1	OLFM2	
	SLC2A9	GALNT17	
	CPNE6	TUBB2B	
	SLC22A2	SALRNA1	
	LINC02373	LRRC38	
	MEIS2	ZFHX4-AS1	
	SNRK-AS1	GSG1L	
	CCDC158	LRFN1	
	PPFIA2	AJAP1	
	PCDH7	SLC6A15	
	TRIM45	ERBB3	
	L3MBTL1	MYCNOS	
	DNAH11	TDRD5	
	BCAN	STUM	
	C9orf47	LINC00621	
	XIAPP3	PRRT4	
	MTSS1L	CHAD	
	ALPK3	MYH14	
	LINC00689	PCDH19	
	SCN2A	RGS6	
	GPR17	TMEM52	
	GLRA4	BTNL2	
	HSPB6	ST8SIA2	
	SGIP1	DISP3	
	ZMYND10	ZNF215	
	KHK	CACHD1	
	SORCS3	ENPP5	
	ABCB5	KNDC1	
	CCDC144NL-AS	FBXO2	
	NALT1	CYTL1	
	LINC02015	LMX1A	
	FERMT2	RCOR2	
	SMOC1	ATP1B2	
	MROH7	EPCAM	
	FAM85B	GAL	
	LINC00881	PPP1R1B	
	MRGPRF	C1QTNF12	
	PPP1R12A	MNX1	
	PHKG1	KCNC3	
	ZBTB16	LINC01801	
	HPGD	NBAT1	
	GADD45G	COLEC11	
	SPANXA2-OT1	CPSF1P1	
	PCDH20	MBL1P	
	MYBL1	ADAMTS3	
	RABGAP1	ADRA2C	
	PAK3	HPCAL4	
	KCNMA1-AS1	KERA	
	BVES-AS1	PTH1R	
	STON1-GTF2A1	DNAH2	
	KCNB1	PNPLA3	
	MTCL1	CFAP46	
	MASP1	PROK2	
	VTN	PCDHA1	
	PDE5A	CELF4	
	OSBPL10	BPIFB4	
	PHKA1	TCERG1L	
	SACS-AS1	CXCL14	
	PLD5	FAM71E1	
	CLU	ELFN1-AS1	
	DPP6	DQX1	
	NACC2	CYP39A1	
	ALDH1L1-AS2	CYS1	
	HEY2	NR2E1	
	TPRG1	FOXF2	
	SGSM1	DACH2	
	CTSV	KRTDAP	
	CHRDL1	ETV4	
	ARTN	CCDC177	
	LINC01193	HCG23	
	PHYHIP	KBTBD7	
	PCA3	ABCA5	
	DCST2	SLC22A31	
	HSPB2	GFRA3	
	TJP2	RTL9	
	PHYHD1	HS3ST4	
	NMNAT2	THSD7B	
	ANO5	DBH-AS1	
	SHISA8	EVX1	
	CA9	JARID2	
	CCDC190	CERS1	
	MEIS1	HRASLS	
	LINC02094	RSPO4	
	ISOC1	FAAH	
	GCNT4	TUBBP5	
	MAATS1	PLK5	
	SLC20A2	HAND1	
	ZPLD1	SCN5A	
	MIR29B2CHG	SEMA6D	
	SEMA4B	FAM19A2	
	STK33	MATN1	
	TMEM47	TRPM5	
	SUSD5	TLE1P1	
	FILIP1L	LINC01738	
	EVA1C	KCNN1	
	FAM181A	MAP6	
	TCF23	USH1C	
	TRDN	HMGCLL1	
	PPM1L		
	ZNF710-AS1		
	BVES		
	PHEX		
	MAGEC3		
	PITPNM3		
	NCS1		
	LGALS9C		
	DEUP1		
	SSXP10		
	SYNC		
	SERPINA6		
	HSPA4L		
	DUSP19		
	ACTA1		
	CNR1		
	IL20		
	DCAF12L1		
	TRIM9		
	NT5DC3		
	CHRM2		
	ANKS1B		
	CDK18		
	RRAD		
	ST6GALNAC5		
	RANBP3L		
	VWA3B		
	RNF112		
	KCNA4		
	RGN		
	LMO3		
	SCN3A		
	SYNE1-AS1		
	B3GAT2		
	SP5		
	LINC01619		
	CBLN1		
	CNTNAP3		
	CCDC110		
	HOPX		
	PPP1R12A-AS1		
	RNF150		
	RGS2		
	MAPT		
	ZNF233		
	PKD1L2		
	LRRC14B		
	LINC01550		
	ABRA		
	LINC01798		
	CDKN2B-AS1		
	COL4A2-AS1		
	ZNF860		
	RGS7BP		
	SH3GL2		
	PRODH		
	CACNA1C-IT2		
	ACSM1		
	RPS10P7		
	NR1H4		
	C22orf23		
	C1QL4		
	C1QL1		
	SLC38A1		
	TES		
	STOX1		
	ADGRL3		
	TMIE		
	LRRC37A9P		
	SEPT3		
	HOGA1		
	KALRN		
	CAMK1		
	FAM218A		
	ST6GAL2		
	B3GALT5		
	PDE3A		
	C6orf223		
	SRF		
	ANO1		
	GRM2		
	SUSD4		
	C9orf135		
	KBTBD13		
	GPR135		
	CACNA1H		
	INTS4P2		
	KRT18P5		
	GNAL		
	FAM189A2		
	NPTN-IT1		
	C1orf61		
	C12orf77		
	LRIG1		
	RDM1		
	ARMC3		
	PRRG4		
	MIR9-3HG		
	HR		
	PLS3-AS1		
	AJM1		
	VCL		
	PGM5P4		
	ELAVL3		
	RPL7P38		
	OVCH2		
	CEND1		
	C2orf40		
	SLC6A17		
	ARNT2		
	LRRC7		
	GIPC3		
	FAM84A		
	KRT18P17		
	C14orf180		
	NKX2-5		
	DMTN		
	RPS6KA5		
	RAB17		
	RN7SL262P		
	FAM178B		
	ATCAY		
	FAM13C		
	PPP1R1A		
	ESR1		
	LGALS9B		
	PGPEP1L		
	LINC01198		
	BCHE		
	CCDC146		
	TRIM63		
	ACOT11		
	RSPO3		
	ILK		
	GPD1L		
	PRDM16		
	GNG4		
	CIPC		
	BEX1		
	NUP210		
	RALYL		
	AHCYP2		
	KCNE4		
	FAM212B		
	GRB14		
	MUC1		
	FHOD3		
	KCNJ3		
	LINC01629		
	LAMA5-AS1		
	TENM2		
	SUPT20HL1		
	NMUR1		
	LSMEM2		
	LBR		
	PPP4R4		
	ART4		
	SLC8A1-AS1		
	ANO3		
	PDE1B		
	THEGL		
	FAM46B		
	SLC9C2		
	STMND1		
	ST5		
	STK32A-AS1		
	TRIM6		
	CX3CL1		
	VTCN1		
	SRC		
	EML6		
	ACTN1-AS1		
	VWA7		
	IL22RA1		
	LINC01799		
	RNF38		
	PDK3		
	ACTN2		
	RTP1		
	RSPO1		
	MLPH		
	NFYC-AS1		
	HAS3		
	CES1		
	PTCHD1-AS		
	FYCO1		
	MRGPRD		
	SLC38A11		
	IGFN1		
	SYNE2		
	NT5M		
	SNORA77		
	FAM212B-AS1		
	SLFNL1-AS1		
	LINC02447		
	LINC01841		
	LAMA5		
	ZNF219		
	HADH		
	LINC00940		
	CT62		
	DLEU2L		
	CCT6P1		
	CPEB4		
	TP53TG5		
	PMEL		
	ACOT1		
	CHRNA3		
	CLSTN2		
	LMNTD1		
	SPTB		
	ATL1		
	SPATA46		
	HSPA2		
	PRSS27		
	NPC1L1		
	INHBA-AS1		
	FAM13B		
	MIR27B		
	FBLIM1		
	NMRK2		
	RNF217-AS1		
	B3GALT5-AS1		
	MIR4786		
	FEM1B		
	NTF3		
	TUBA4A		
	PPFIA2-AS1		
	OR10AB1P		
	GPR62		
	MTNR1B		
	ANKRD45		
	LRRC36		
	BARD1		
	ABCC8		
	DMPK		
	PDLIM7		
	GREM2		
	TMEM108		
	PPIAP16		
	PCSK1		
	RPS6KL1		
	ANKRD20A18P		
	SPINK2		
	SSTR1		
	AKT3		
	LINC00310		
	SARM1		
	RASGRP2		
	KCTD19		
	RAB3C		
	RND2		
	FPGT-TNNI3K		
	ANKRD20A10P		
	PDZK1IP1		
	EMILIN1		
	TRPC5OS		
	AGAP11		
	AMIGO2		
	PART1		
	MCHR1		
	SNAP25-AS1		
	BHLHA15		
	EEF1A2		
	CKB		
	FOXN4		
	CNTNAP3B		
	SAMMSON		
	PHKA1P1		
	LARGE1		
	SH3BGR		
	MIR3164		
	LINC01366		
	PPP1R14C		
	UBE2QL1		
	PPP1R17		
	SERTM1		
	PAK5		
	PLCL1		
	RERG-IT1		
	VLDLR		
	ITIH3		
	MYO3A		
	WDR17		
	CTF1		
	EEF1A1P33		
	GJC1		
	TYRP1		
	RGS22		
	LINC02145		
	OSTN		
	KCNB2		
	CPXM2		
	EP300-AS1		
	BRIP1		
	SLC26A7		
	LRRN4		
	STMN4		
	HSD17B1P1		
	CSPG4		
	PGM5P4-AS1		
	VLDLR-AS1		
	USP3-AS1		
	TMEM61		
	TTBK1		
	ATP2B2		
	SPINK13		
	CFAP221		
	HERC5		
	CYP21A1P		
	ALDH3A2		
	CNTNAP3P2		
	MYLKP1		
	PRL		
	ITLN2		
	PALM2-AKAP2		
	FMO5		
	MKX		
	LINC01123		
	CCNE2		
	CMTM5		
	CORO6		
	STK32A		
	GRIA2		
	PRDX3P1		
	CRTAC1		
	LINC01229		
	SEPT7P9		
	LINC00473		
	FAM124A		
	ALG1L13P		
	NDUFA3P4		
	AARD		
	NOXA1		
	KRT9		
	ANKRD20A21P		
	EDN2		
	TMEM27		
	CACNA1C-AS1		
	MUC4		
	TMEM40		
	WNK2		
	LINC01278		
	S1PR3		
	MAST4-AS1		
	KIAA0408		
	SAMD13		
	PLA2G10		
	CHIA		
	LONRF1		
	XIST		
	SYT10		
	HHATL		
	SFRP1		
	ILDR2		
	LINC00298		
	MUC20P1		
	SLC47A2		
	ANKRD20A4		
	CCDC185		
	ERN2		
	C7		
	LINC01944		
	TPTE2P3		
	SLC5A2		
	ENPP7P1		
	C16orf89		
	KCNJ4		
	LINC01124		

Table S10: Gene markers for BJ cell line from Pollen dataset

K_1	K_2	K_3
	RPL21	RPSAP58
	RPL7	PABPC3
	BZW1	COX6A1P2
	H3F3A	H3F3C
	RPS3A	ZC3H14
	CRK	ARNTL2
	RPL9	KDM2A
	RPS27	TVP23B
	LSM12	ACADM
	FABP5	HIPK2
	RPL12	HECTD1
	PPIA	ZFR
	RPS26	TOB1
	RPSA	TNRC6B
	RPL18A	NUDCD3
	RPL41	AHCYL1
	PSMC1	P4HA2
	RPLP0	CLMP
	RPS28	M6PR
	RPL23A	PITRM1
	EEF1A1	NQO1
	RPL13A	C1GALT1C1
	NQO1	REST
	HMGN2	TRAK2
	RPS7	PEAK1
	RPL10	NCOR1
	HNRNPA1	GPNMB
	SPCS2	MYL9
	PGAM1	RBM3
	EEF1B2	GPR176
	C4orf32	PGD
	RPL6	NDUFS2
	VDAC2	ANXA4
	NPM1	PGK1
	RPL7A	GLIPR2
	MELK	LRP12
	EIF4E	ETNK1
	ITGB1	MEIS1
	ADAM9	MTCH1
	NHP2	SKIV2L2
	DNAJB6	PPP4R1
	NKTR	EIF4G3
	RAP1B	ANO6
		TGFBR1
		CAP2
		MGLL
		PTK2
		AXL
		PSMD1
		CBX1
		SLC44A1
		SACS
		APC
		CTTN
		CAMK2D
		PPFIBP1
		CSRP1
		FUT8
		CAV1
		SARS
		RAB10
		IFI16
		PNPO
		IFITM3
		CAST
		PSMC4
		OXTR
		KRT7
		PAM

Table S11: Gene markers for Oligodendrocytes cells from Darmanis dataset

K_1	K_2	K_3
HNRNPH1	FTH1	ABCA2
PPP1CB	HSPA1A	MAG
PIP4K2A	TMSB4X	APLP1
SLC12A2	MOG	MOG
TRA2A	FTL	PHLDB1
TSPYL1	RPL23A	ELOVL1
CDC42	PTGDS	BIN1
TOB1	MAL	MAP7D1
DOCK5	ABCA2	TUBB4A
KCNJ2	RPS15	STMN4
SERINC5	TUBB4A	HSPA2
CAPZA1		TSC22D4
PEG3		ADAMTS4
CLDN11		MAL
DPYSL2		HAPLN2
FNIP1		SYNGR2
MAP1B		TTYH1
WTAP		DDR1
PTBP2		CDC42EP1
NFIA		ACTB
ARRDC3		CDK18
CXADR		EFHD1
FOXN2		PLLP
SET		JUNB
CPEB4		NACAD
CTNNB1		GPRC5B
TRIM59		TUFM
ANKRD40		PTGDS
TIAL1		SHISA4
CDC42BPA		TECR
TAOK1		GSN
STRN3		MARCKSL1
SRSF6		CMTM5
HMGCS1		RNH1
TBL1XR1		SIRT2
SLITRK2		PSAP
YWHAG		PREX1
R3HDM2		PRKCSH
CD47		PTOV1
ANK2		GAPDH
PAFAH1B2		RGL1
		CSRP1
		CKB
		COMT
		GAMT
		PLEKHB1
		NKX6-2
		GNPTG
		GUK1

Table S12: Gene markers for endothelial cells from darmanis dataset

K_1	K_2	K_3
TAGLN	PODXL	PTGDS
RGS5	NEDD9	DCN
NOTCH3	A2M	FBLN1
HIGD1B	S1PR1	C7
DCN	TM4SF18	COL1A2
PDGFRB	ITM2A	OGN
MYO1B	ABCG2	C1S
PLXDC1	GPR116	KIAA1199
GJC1	CLDN5	COL1A1
NDUFA4L2	RNASE1	APOD
S1PR3	IFI27	CYP1B1
ARHGEF7	FOXF1	CXCL12
CPM	ABLIM1	ISLR
COL1A2	ELTD1	PCOLCE
ENPEP	ESAM	CLU
MYL9	LXN	OLFML3
CRIP1	EMP1	MFAP4
CD248	SLC39A10	C1R
ABCC9	CD34	RARRES2
TPM2	ICAM2	CLDN11
CDH6	DOCK9	FBLN2
CCDC102B	C1orf64	MGP
OLFML2A	VWF	THBS2
SEMA5A	CDH5	COL6A1
ADAMTS4	C1orf54	VCAN
EDNRA	MGST2	MXRA8
ARHGEF17	ROBO4	FMOD
PTN	LPHN2	LTBP2
PLCE1	ECSCR	THBS1
PPP1R14A	FLT1	MMP2
ATP1A2	PLEKHG1	COL3A1
EHD2	RELL1	FLRT2
COL5A2		PTN
LGALS1		GFPT2
		PRELP
		FBLN5
		CDR1
		IGFBP2
		SERPING1
		S100A6
		LAMA2
		AKR1C1
		IGFBP6
		CYBRD1
		SFRP4
		COL6A3
		CEBPB
		LGALS3BP
		NOTCH2NL
		PDGFRB
		CCDC80
		NR2F1
		ABCA9
		PLTP
		LUM
		ATP1A2
		COL6A2
		NR4A3
		MRC2
		PLAC9

Table S13: Gene markers for fetal quiescent cells from Darmanis dataset

K_1	K_2	K_3
	PLXNA4	FAM49A
	EPHA3	LMO7
	FRMD4B	FBXW7
	DOK5	EFNA5
	SEMA3A	LMO3
	SATB2	GRM5
	LIMCH1	CNTN1
		SLC35F1
		LDB2
		CAMKV
		CD47
		ZDHHC2
		PRICKLE2
		DLG2
		PGM2L1

Table S14: Macrophage clusters as determined by RCC from Netfel dataset

Clusters	Microglia geneset	Macrophage geneset	Hypoxic geneset	Non_hypoxic geneset	Complement pathway	Molecular Function	Biological Process	Cellular Component	Pathway	Genes
1	present						positive regulation of myeloid cell differentiation	transcription factor AP-1 complex	IL6-mediated signaling events, METS affect on Macrophage Differentiation, HIF-1-alpha transcription factor network	CSF1R,P2RY12, SELPLG, GPR56
2	present	present	present				Regulation of immune system process	complement component C1 complex, blood microparticle	Classical Complement Pathway	C1QA,C1QB, C1QC
3		present	present			ribosome binding	purine ribonucleoside triphosphate biosynthetic process	secretory granule lumen, mitochondrial part		EIF5A,RPSA, EIF2S1
4		present	present		present	glyceraldehyde-3-phosphate dehydrogenase (NAD+) (phosphorylating) activity	glucose catabolic process, myeloid leukocyte mediated immunity, myeloid leukocyte activation, neutrophil activation	tertiary granule, myelin sheath	HIF-1-alpha transcription factor network, glycolysis	HK2,NDRG1, HMOX1,ALDOA,LDHA,ENO1,PLIN2
5		present	present		present			cytoplasmic vesicle membrane		CD55,ANXA1, RAC2
6					present		actin filament network formation	focal adhesion		LCP1,ARPC5
7										
8						DNA-binding transcription activator activity, RNA polymerase II-specific	positive regulation of chemokine biosynthetic process	nuclear chromatin	Interleukin-10 signaling, Toll-like receptor signaling pathway	IL1B,CCL3L2, CCL3,EGR1,CD74
9				present						

Supplementary File 1 - Human tissue dataset : Gender association with RCC clusters

SMTSD	Run_1	Run_2	Run_3	Run_4	Run_5	Run_6	Run_7	Run_8	Run_9	Run_10	p-val <= 0.0
Breast - Mammary Tissue	0.00050	0.00050	0.00050	0.00050	0.00050	0.00050	0.00050	0.00050	0.00050	0.00050	10
Skin - Not Sun Exposed (Suprapubic)	0.00300	0.00550	0.00250	0.00100	0.00100	0.00300	0.00300	0.00250	0.00400	0.00350	10
Brain - Cerebellum	0.00094	0.00094	0.00094	0.00183	0.00183	0.00183	0.00183	0.00094	0.00094	0.00183	10
Pancreas	0.00050	0.00050	0.00050	0.00100	0.00050	0.00100	0.00050	0.00100	NA	0.00050	9
Thyroid	0.01000	0.00050	0.01249	0.00850	0.00050	0.00200	0.00100	0.00050	0.00050	0.03348	8
Adipose - Visceral (Omentum)	0.00050	0.00050	0.06997	0.03648	0.00350	0.00050	0.00050	0.14343	0.00050	0.00050	7
Skin - Sun Exposed (Lower leg)	0.07596	0.00150	0.00100	0.11994	0.00350	0.00200	0.00350	0.00050	0.00350	0.01249	7
Adipose - Subcutaneous	0.00050	0.06982	0.06816	0.11161	0.08819	0.00050	0.17029	0.05350	0.06816	0.11081	2
Brain - Cortex	NA	NA	NA	NA	NA	NA	NA	0.00050	0.00050	NA	2
Cells - Transformed fibroblasts	0.44378	0.22739	0.60770	0.02599	0.00700	0.22239	0.12594	0.00550	0.16242	0.46727	2
Small Intestine - Terminal Ileum	0.45927	0.04607	0.00750	0.23825	0.04607	0.11817	0.01299	0.12391	0.12370	0.03198	1
Liver	0.51224	0.49525	0.00100	0.51024	0.53523	0.45977	0.44759	0.51274	0.44759	0.34300	1
Muscle - Skeletal	0.11844	0.14543	0.13143	0.14343	0.14343	0.07696	0.14593	0.12694	0.11594	0.13643	0
Artery - Tibial	0.43553	0.02249	0.05147	0.03498	0.01599	0.02299	0.02249	0.03948	0.04248	0.12144	0
Artery - Coronary	0.66167	0.51174	0.49275	0.36582	0.66549	0.35532	0.66817	0.34583	0.65330	NA	0
Heart - Atrial Appendage	0.06347	0.05547	0.14343	0.05197	0.04748	0.05047	0.05047	0.05497	0.03048	0.03898	0
Ovary	NA	NA	NA	NA	NA	NA	NA	NA	NA	NA	0
Uterus	NA	NA	NA	NA	NA	NA	NA	NA	NA	NA	0
Vagina	NA	NA	NA	NA	NA	NA	NA	NA	NA	NA	0
Minor Salivary Gland	0.73711	0.73711	0.73711	0.73711	0.73711	0.73711	0.73711	0.73711	0.73386	0.73711	0
Adrenal Gland	0.60670	0.59670	0.81291	0.63618	0.62969	0.63168	0.58871	0.65467	0.64818	0.59170	0
Lung	0.04098	0.04648	0.04198	0.02649	0.03698	0.01449	0.02499	0.04748	0.04598	0.03998	0
Spleen	NA	0.25182	0.25182	0.25182	NA	0.12798	NA	NA	NA	NA	0
Esophagus - Muscularis	0.09195	0.06097	0.08846	0.03598	0.05397	0.03448	0.03148	0.02749	0.03598	0.03548	0
Esophagus - Mucosa	0.04798	0.12494	0.10595	0.36482	0.80360	0.04048	0.03948	0.32884	0.75812	0.36032	0
Esophagus - Gastroesophageal Junction	0.82809	0.72564	0.89955	0.80760	0.79660	0.70715	0.53123	0.52274	0.72964	0.59770	0
Stomach	1.00000	1.00000	0.85651	0.72198	1.00000	0.85306	0.85717	0.85306	0.13243	0.85704	0
Colon - Sigmoid	0.84608	0.14193	0.17941	0.13843	0.09295	1.00000	0.84608	1.00000	0.84622	0.84608	0
Colon - Transverse	0.22639	0.23538	0.37781	0.15192	0.57871	0.08396	0.12644	0.06197	0.12044	0.16642	0
Prostate	NA	NA	NA	NA	NA	NA	NA	NA	NA	NA	0
Testis	NA	NA	NA	NA	NA	NA	NA	NA	NA	NA	0
Nerve - Tibial	0.10595	0.11794	0.08346	0.10245	0.06747	0.09795	0.06547	0.09245	0.09995	0.07846	0
Heart - Left Ventricle	1.00000	1.00000	0.86575	1.00000	0.74688	1.00000	1.00000	1.00000	0.86575	0.87107	0
Whole Blood	0.01199	0.06897	0.03098	0.04398	0.02199	0.03848	0.04198	0.03548	0.03548	0.05047	0
Artery - Aorta	0.21539	0.83658	0.56922	0.74532	0.74319	0.84008	0.68916	0.74532	0.87556	NA	0
Cells - EBV-transformed lymphocytes	0.48326	0.41429	0.36882	0.47276	0.37031	0.40315	0.37431	0.37831	0.35582	0.34633	0
Pituitary	0.48956	0.48956	0.48956	0.81212	0.48956	0.48956	0.48956	0.48956	0.48956	0.48956	0
Kidney - Cortex	NA	NA	NA	NA	NA	NA	NA	NA	NA	NA	0
Brain - Hippocampus	1.00000	0.61791	1.00000	1.00000	1.00000	0.61559	1.00000	1.00000	0.76462	1.00000	0
Brain - Substantia nigra	0.16857	0.36575	0.50915	NA	0.27049	0.54273	0.54273	0.77861	0.54273	0.50915	0
Brain - Anterior cingulate cortex (BA24)	0.43525	0.43525	0.43525	0.43525	0.43525	0.57166	0.20575	0.36682	0.36832	0.43525	0
Brain - Frontal Cortex (BA9)	NA	NA	NA	NA	NA	NA	NA	0.02249	0.03348	NA	0
Brain - Cerebellar Hemisphere	0.43701	0.43701	0.43701	0.14619	0.13373	0.14619	0.14619	0.43701	0.43701	0.14619	0
Brain - Caudate (basal ganglia)	0.51024	0.37581	0.48076	0.86507	0.65217	0.39780	0.43228	0.45227	0.37381	0.65617	0
Brain - Nucleus accumbens (basal ganglia)	0.16105	0.04412	0.09212	0.04412	0.09212	0.08726	0.07756	0.08726	0.04412	0.09212	0
Brain - Putamen (basal ganglia)	0.62643	0.81201	0.63112	0.81201	0.63640	0.81201	0.81201	0.81201	0.81201	0.62893	0
Brain - Hypothalamus	NA	NA	NA	1.00000	0.03598	NA	NA	0.11244	NA	NA	0
Brain - Spinal cord (cervical c-1)	0.04479	NA	0.08315	NA	0.31824	NA	NA	0.13246	NA	0.30648	0
Brain - Amygdala	0.55459	0.55459	0.77024	0.76543	0.55459	0.76027	0.76543	0.76543	0.08446	0.55459	0
Fallopian Tube	NA	NA	NA	NA	NA	NA	NA	NA	NA	NA	0
Bladder	NA	NA	NA	NA	NA	NA	NA	NA	NA	NA	0
Cervix - Ectocervix	NA	NA	NA	NA	NA	NA	NA	NA	NA	NA	0
Cervix - Endocervix	NA	NA	NA	NA	NA	NA	NA	NA	NA	NA	0

Supplementary File 1 - Human tissue dataset : Age association with RCC clusters

SMTSD	Run_1	Run_2	Run_3	Run_4	Run_5	Run_6	Run_7	Run_8	Run_9	Run_10	p-val <= 0.[illegible]
Muscle - Skeletal	0.00150	0.00050	0.00050	0.00050	0.00050	0.00100	0.00050	0.00100	0.00100	0.00150	10
Thyroid	0.00300	0.00050	0.00250	0.00850	0.00100	0.00050	0.00200	0.00400	0.00100	0.00350	10
Esophagus - Mucosa	0.00050	0.00050	0.00050	0.00100	0.00150	0.00050	0.00050	0.00050	0.00200	0.00050	10
Colon - Sigmoid	0.00050	0.00050	0.00050	0.00050	0.00050	0.00050	0.00100	0.00050	0.00100	0.00050	10
Whole Blood	0.00050	0.00050	0.00050	0.00050	0.00050	0.00050	0.00050	0.00050	0.00050	0.00100	10
Adipose - Subcutaneous	0.00350	0.00300	0.00400	0.00350	0.00400	0.03898	0.00400	0.00950	0.00600	0.00250	9
Artery - Tibial	0.20790	0.00250	0.00450	0.00250	0.00300	0.00150	0.00350	0.00300	0.00050	0.00150	9
Adipose - Visceral (Omentum)	0.00950	0.01549	0.00700	0.00550	0.02299	0.02049	0.01399	0.00950	0.02199	0.00450	5
Liver	0.00750	0.00600	0.03098	0.00650	0.00900	0.00400	0.64918	0.01199	0.64568	0.49475	5
Lung	0.01549	0.02099	0.01649	0.00550	0.01599	0.00550	0.00150	0.01249	0.00900	0.01799	4
Colon - Transverse	0.00500	0.92104	0.15092	0.21139	0.58071	0.00800	0.01699	0.00250	0.00200	0.01549	4
Artery - Aorta	0.00050	0.13643	0.40380	0.13543	0.15192	0.00650	0.00050	0.12444	0.09645	NA	3
Prostate	0.00100	NA	NA	NA	NA	NA	NA	NA	NA	0.00050	2
Cells - Transformed fibroblasts	0.04948	0.10095	0.04598	0.01999	0.00350	0.11694	0.09845	0.00250	0.09595	0.04798	2
Skin - Sun Exposed (Lower leg)	0.03248	0.06247	0.05647	0.04748	0.00900	0.02849	0.04148	0.05597	0.04598	0.01949	1
Artery - Coronary	0.06197	0.10045	0.02649	0.08996	0.89455	0.04548	0.12294	0.09445	0.55422	NA	0
Heart - Atrial Appendage	0.37831	0.34133	0.62619	0.33283	0.47976	0.39530	0.36632	0.48276	0.46177	0.38281	0
Ovary	NA	0.68516	NA	NA	NA	NA	NA	0.30435	NA	NA	0
Uterus	NA	NA	NA	NA	NA	NA	NA	NA	NA	NA	0
Vagina	0.37531	0.64968	0.46827	0.64268	0.15192	0.12944	0.44978	0.44678	0.30835	0.43478	0
Breast - Mammary Tissue	0.72964	0.08296	0.17991	0.23738	0.42179	0.58221	0.41579	0.54073	0.28486	0.36432	0
Skin - Not Sun Exposed (Suprapubic)	0.79310	0.25787	0.27436	0.75762	0.06347	0.42779	0.40230	0.30735	0.40980	0.43828	0
Minor Salivary Gland	0.80110	0.79460	0.79210	0.79460	0.79260	0.78011	0.77111	0.78811	0.65817	0.78361	0
Brain - Cortex	NA	NA	NA	NA	NA	NA	NA	0.39280	0.35832	NA	0
Adrenal Gland	0.07596	0.12694	0.69665	0.07596	0.08646	0.09195	0.06297	0.27486	0.16242	0.06347	0
Spleen	NA	0.32934	0.33983	0.35032	NA	0.16142	NA	NA	NA	NA	0
Pancreas	0.25937	0.52074	0.37831	0.39030	0.52074	0.36682	0.37281	0.34833	NA	0.19440	0
Esophagus - Muscularis	0.17691	0.23138	0.19640	0.12944	0.05647	0.16292	0.14993	0.11194	0.24288	0.27636	0
Esophagus - Gastroesophageal Junction	0.05797	0.04648	0.06047	0.06847	0.05947	0.01949	0.06147	0.04498	0.10545	0.06947	0
Stomach	0.30535	0.30985	0.33633	0.30835	0.30385	0.22639	0.36732	0.24038	0.42829	0.36032	0
Small Intestine - Terminal Ileum	0.48676	0.22789	0.50025	0.87706	0.20890	0.31434	0.46327	0.42929	0.29235	0.24788	0
Testis	0.32934	0.27336	0.37181	0.24788	0.26987	0.38431	0.28636	0.31334	0.37431	0.26537	0
Nerve - Tibial	0.28986	0.25087	0.28786	0.26437	0.29185	0.29785	0.25237	0.32234	0.23688	0.30835	0
Heart - Left Ventricle	0.03298	0.03548	0.02249	0.03498	0.04048	0.02399	0.02949	0.01699	0.02049	0.01799	0
Brain - Cerebellum	0.09995	0.09645	0.09295	0.16992	0.14743	0.15242	0.15992	0.10745	0.09145	0.15642	0
Cells - EBV-transformed lymphocytes	0.13293	0.07646	0.14393	0.10845	0.15992	0.05247	0.11244	0.14543	0.10095	0.14593	0
Pituitary	0.85057	0.85707	0.85307	0.83558	0.85407	0.86507	0.86107	0.85707	0.83608	0.84308	0
Kidney - Cortex	NA	NA	NA	NA	NA	NA	NA	NA	NA	NA	0
Brain - Hippocampus	0.43578	0.84858	0.18441	0.70565	0.49375	0.80510	0.32584	0.30035	0.39130	0.52074	0
Brain - Substantia nigra	0.11594	0.31884	0.20590	NA	0.03048	0.33483	0.33383	0.37581	0.33533	0.20340	0
Brain - Anterior cingulate cortex (BA24)	0.88456	0.88456	0.88006	0.88406	0.90405	0.96052	0.87556	0.05697	0.05197	0.85957	0
Brain - Frontal Cortex (BA9)	NA	NA	NA	NA	NA	NA	NA	0.31084	0.22539	NA	0
Brain - Cerebellar Hemisphere	0.16192	0.14843	0.15792	0.17391	0.25587	0.16942	0.17541	0.16042	0.16092	0.16992	0
Brain - Caudate (basal ganglia)	0.02799	0.11294	0.03198	0.05297	0.07746	0.13343	0.10695	0.09995	0.11394	0.06097	0
Brain - Nucleus accumbens (basal ganglia)	0.28786	0.40780	0.41479	0.40430	0.43078	0.28186	0.42379	0.28436	0.40880	0.43178	0
Brain - Putamen (basal ganglia)	0.17491	0.84908	0.13193	0.82859	0.19890	0.84908	0.84908	0.83758	0.83758	0.29585	0
Brain - Hypothalamus	NA	NA	NA	0.05047	0.07646	NA	NA	0.02699	NA	NA	0
Brain - Spinal cord (cervical c-1)	0.58971	NA	0.69915	NA	0.63618	NA	NA	0.77211	NA	0.50625	0
Brain - Amygdala	0.35332	0.35282	0.46477	0.48426	0.33383	0.33783	0.52674	0.50225	0.80360	0.32334	0
Fallopian Tube	NA	NA	NA	NA	NA	NA	NA	NA	NA	NA	0
Bladder	NA	NA	NA	NA	NA	NA	NA	NA	NA	NA	0
Cervix - Ectocervix	NA	NA	NA	NA	NA	NA	NA	NA	NA	NA	0
Cervix - Endocervix	NA	NA	NA	NA	NA	NA	NA	NA	NA	NA	0

Supplementary File 1 - Human tissue dataset : Cause of death (COD) association with RCC clusters

SMTSD	Run_1	Run_2	Run_3	Run_4	Run_5	Run_6	Run_7	Run_8	Run_9	Run_10	p-val <= 0.0
Adipose - Subcutaneous	0.000500	0.000500	0.000500	0.000500	0.000500	0.000500	0.000500	0.000500	0.000500	0.000500	10
Muscle - Skeletal	0.000500	0.000500	0.000500	0.000500	0.000500	0.000500	0.000500	0.000500	0.000500	0.000500	10
Heart - Atrial Appendage	0.000500	0.000500	0.000500	0.000500	0.000500	0.000500	0.000500	0.000500	0.000500	0.000500	10
Adipose - Visceral (Omentum)	0.000500	0.000500	0.000500	0.000500	0.000500	0.000500	0.000500	0.000500	0.000500	0.000500	10
Breast - Mammary Tissue	0.001000	0.000500	0.000500	0.000500	0.000500	0.000500	0.000500	0.000500	0.000500	0.000500	10
Skin - Not Sun Exposed (Suprapubic)	0.000500	0.000500	0.000500	0.000500	0.000500	0.000500	0.000500	0.000500	0.000500	0.000500	10
Thyroid	0.000500	0.000500	0.000500	0.000500	0.000500	0.000500	0.000500	0.000500	0.000500	0.000500	10
Lung	0.000500	0.000500	0.000500	0.000500	0.000500	0.000500	0.000500	0.000500	0.000500	0.000500	10
Esophagus - Muscularis	0.000500	0.000500	0.000500	0.000500	0.000500	0.000500	0.000500	0.000500	0.000500	0.000500	10
Esophagus - Mucosa	0.000500	0.000500	0.000500	0.000500	0.000500	0.000500	0.000500	0.000500	0.000500	0.000500	10
Esophagus - Gastroesophageal Junction	0.000500	0.000500	0.000500	0.000500	0.000500	0.000500	0.000500	0.000500	0.000500	0.000500	10
Nerve - Tibial	0.000500	0.000500	0.000500	0.000500	0.000500	0.000500	0.000500	0.000500	0.000500	0.000500	10
Skin - Sun Exposed (Lower leg)	0.000500	0.000500	0.000500	0.000500	0.000500	0.000500	0.000500	0.000500	0.000500	0.000500	10
Heart - Left Ventricle	0.000500	0.000500	0.000500	0.000500	0.000500	0.000500	0.000500	0.000500	0.000500	0.000500	10
Brain - Cerebellum	0.000500	0.000500	0.000500	0.000500	0.000500	0.000500	0.000500	0.000500	0.000500	0.001000	10
Whole Blood	0.000500	0.000500	0.000500	0.000500	0.000500	0.000500	0.000500	0.000500	0.000500	0.000500	10
Cells - EBV-transformed lymphocytes	0.005497	0.001999	0.002499	0.004498	0.004998	0.000500	0.002499	0.005497	0.003998	0.003498	10
Liver	0.000500	0.000500	0.000500	0.000500	0.000500	0.000500	0.000500	0.000500	0.000500	0.000500	10
Brain - Cerebellar Hemisphere	0.001999	0.002999	0.004998	0.001000	0.000500	0.001000	0.001000	0.001999	0.004998	0.001499	10
Artery - Tibial	0.750625	0.000500	0.000500	0.000500	0.000500	0.000500	0.000500	0.000500	0.000500	0.000500	9
Adrenal Gland	0.000500	0.000500	0.236382	0.000500	0.000500	0.000500	0.000500	0.000500	0.000500	0.000500	9
Colon - Transverse	0.000500	0.202899	0.000500	0.000500	0.594703	0.000500	0.000500	0.000500	0.000500	0.001000	8
Artery - Coronary	0.000500	0.000500	0.000500	0.000500	0.165917	0.000500	0.000500	0.000500	0.253873	NA	7
Colon - Sigmoid	0.023488	0.000500	0.000500	0.000500	0.000500	0.002999	0.015992	0.000500	0.023488	0.014493	6
Cells - Transformed fibroblasts	0.097451	0.006997	0.066467	0.004998	0.022989	0.006497	0.001999	0.036482	0.001499	0.096952	5
Brain - Spinal cord (cervical c-1)	0.007496	NA	0.001999	NA	0.001499	NA	NA	0.002499	NA	0.001000	5
Artery - Aorta	0.000500	0.001499	0.052974	0.268366	0.315842	0.081459	0.000500	0.253873	0.196902	NA	3
Brain - Cortex	NA	NA	NA	NA	NA	NA	NA	0.000500	0.000500	NA	2
Brain - Anterior cingulate cortex (BA24)	0.063468	0.078961	0.059470	0.059970	0.063468	0.448276	0.052974	0.009495	0.004998	0.055972	2
Brain - Frontal Cortex (BA9)	NA	NA	NA	NA	NA	NA	NA	0.000500	0.000500	NA	2
Ovary	NA	0.632684	NA	NA	NA	NA	NA	0.000500	NA	NA	1
Pancreas	0.007496	0.010495	0.016492	0.014993	0.011994	0.010995	0.016992	0.016492	NA	0.063968	1
Uterus	NA	NA	NA	NA	NA	NA	NA	NA	NA	NA	0
Vagina	0.150425	0.302349	0.063468	0.294353	0.030485	0.021989	0.058971	0.053473	0.035482	0.055972	0
Minor Salivary Gland	0.439780	0.433283	0.438781	0.441779	0.431784	0.424788	0.432784	0.425287	0.556722	0.435282	0
Spleen	NA	0.493753	0.513243	0.497251	NA	0.480260	NA	NA	NA	NA	0
Stomach	0.362319	0.232884	0.249875	0.268866	0.252374	0.200900	0.388306	0.195402	0.073463	0.369315	0
Small Intestine - Terminal Ileum	0.706647	1.000000	0.510245	0.675662	1.000000	1.000000	0.170915	1.000000	0.419290	0.157921	0
Prostate	0.130935	NA	NA	NA	NA	NA	NA	NA	NA	0.126937	0
Testis	0.242379	0.196902	0.137931	0.198401	0.218391	0.143928	0.209395	0.171914	0.121439	0.204898	0
Pituitary	0.325337	0.348826	0.355322	0.382309	0.356822	0.350825	0.342829	0.338331	0.363318	0.337331	0
Kidney - Cortex	NA	NA	NA	NA	NA	NA	NA	NA	NA	NA	0
Brain - Hippocampus	0.974513	0.915542	1.000000	0.988006	0.890555	0.900550	0.975512	0.973513	0.972014	0.894553	0
Brain - Substantia nigra	0.555222	0.450275	0.220890	NA	0.362319	0.630685	0.629685	0.343328	0.636682	0.236382	0
Brain - Caudate (basal ganglia)	0.484258	0.139930	0.204398	0.192904	0.057971	0.141429	0.125437	0.114443	0.142429	0.051474	0
Brain - Nucleus accumbens (basal ganglia)	0.062469	0.127436	0.103448	0.137931	0.100950	0.071464	0.079960	0.070965	0.110945	0.105447	0
Brain - Putamen (basal ganglia)	0.238881	0.481759	0.284858	0.502249	0.260870	0.503748	0.500250	0.492254	0.512744	0.212394	0
Brain - Hypothalamus	NA	NA	NA	0.213893	0.145427	NA	NA	0.624688	NA	NA	0
Brain - Amygdala	0.494753	0.511744	0.522239	0.490255	0.525237	0.494253	0.468266	0.502749	0.664168	0.517741	0
Fallopian Tube	NA	NA	NA	NA	NA	NA	NA	NA	NA	NA	0
Bladder	NA	NA	NA	NA	NA	NA	NA	NA	NA	NA	0
Cervix - Ectocervix	NA	NA	NA	NA	NA	NA	NA	NA	NA	NA	0
Cervix - Endocervix	NA	NA	NA	NA	NA	NA	NA	NA	NA	NA	0

Supplementary File 1 - Human tissue dataset : Gender, Age, COD association with tSNE + k-means clusters

SMTSD	Gender	Age	COD
Adipose - Visceral (Omentum)	0.01110	0.00050	0.00050
Esophagus - Muscularis	0.06997	0.00050	0.00050
Whole Blood	0.10045	0.00050	0.00050
Lung	0.28432	0.00050	0.00050
Adipose - Subcutaneous	0.42132	0.00050	0.00050
Colon - Sigmoid	0.47727	0.00050	0.00050
Colon - Transverse	0.53873	0.00050	0.00050
Artery - Aorta	1.00000	0.00050	0.00050
Esophagus - Gastroesophageal Junction	0.05347	0.00450	0.00050
Nerve - Tibial	0.32943	0.01149	0.00050
Muscle - Skeletal	0.11394	0.02099	0.00050
Skin - Not Sun Exposed (Suprapubic)	0.01522	0.02199	0.00050
Skin - Sun Exposed (Lower leg)	0.43646	0.06097	0.00050
Artery - Coronary	0.83819	0.06647	0.00050
Breast - Mammary Tissue	0.00050	0.31534	0.00050
Adrenal Gland	0.85419	0.38681	0.00050
Stomach	0.22189	0.05097	0.00100
Testis	NA	0.18191	0.00150
Brain - Hippocampus	0.41279	0.12194	0.08596
Vagina	NA	0.59270	0.11144
Brain - Nucleus accumbens (basal ganglia)	0.18067	0.15742	0.12444
Brain - Hypothalamus	0.70465	0.55222	0.22189
Brain - Caudate (basal ganglia)	0.50282	0.27436	0.30735
Brain - Frontal Cortex (BA9)	0.31439	0.54573	0.43278
Brain - Anterior cingulate cortex (BA24)	0.28460	0.78661	0.44578
Brain - Amygdala	0.03420	0.70565	0.66467
Brain - Putamen (basal ganglia)	0.80174	0.73063	0.93303
Small Intestine - Terminal Ileum	0.35625	0.36732	1.00000
Minor Salivary Gland	0.05575	0.98601	1.00000
Artery - Tibial	NA	NA	NA
Heart - Atrial Appendage	NA	NA	NA
Ovary	NA	NA	NA
Uterus	NA	NA	NA
Brain - Cortex	NA	NA	NA
Thyroid	NA	NA	NA
Spleen	NA	NA	NA
Pancreas	NA	NA	NA
Esophagus - Mucosa	NA	NA	NA
Prostate	NA	NA	NA
Heart - Left Ventricle	NA	NA	NA
Brain - Cerebellum	NA	NA	NA
Cells - Transformed fibroblasts	NA	NA	NA
Cells - EBV-transformed lymphocytes	NA	NA	NA
Pituitary	NA	NA	NA
Liver	NA	NA	NA
Kidney - Cortex	NA	NA	NA
Brain - Substantia nigra	NA	NA	NA
Brain - Cerebellar Hemisphere	NA	NA	NA
Brain - Spinal cord (cervical c-1)	NA	NA	NA
Fallopian Tube	NA	NA	NA
Bladder	NA	NA	NA
Cervix - Ectocervix	NA	NA	NA
Cervix - Endocervix	NA	NA	NA

Supplementary File 1 - Human tissue dataset : Gender, Age, COD association with hclust clusters

SMTSD	Gender	Age	COD
Adipose - Subcutaneous	0.02499	0.89255	0.01000
Muscle - Skeletal	NA	NA	NA
Artery - Tibial	0.04698	0.22539	0.42279
Artery - Coronary	1.00000	0.40730	0.75612
Heart - Atrial Appendage	0.04185	0.75562	0.03648
Adipose - Visceral (Omentum)	NA	NA	NA
Ovary	NA	NA	NA
Uterus	NA	NA	NA
Vagina	NA	0.23238	0.15042
Breast - Mammary Tissue	0.00050	0.63018	0.74663
Skin - Not Sun Exposed (Suprapubic)	NA	NA	NA
Minor Salivary Gland	NA	NA	NA
Brain - Cortex	NA	NA	NA
Adrenal Gland	0.03952	0.84458	0.06247
Thyroid	0.11108	0.19340	0.53223
Lung	NA	NA	NA
Spleen	NA	NA	NA
Pancreas	NA	NA	NA
Esophagus - Muscularis	0.00200	0.95202	0.01199
Esophagus - Mucosa	0.10231	0.00050	0.00050
Esophagus - Gastroesophageal Junction	0.54531	0.26837	0.40480
Stomach	0.26581	0.28086	0.29635
Colon - Sigmoid	0.39424	0.72014	0.90655
Small Intestine - Terminal Ileum	0.13952	0.14943	0.11294
Colon - Transverse	0.28686	0.00100	0.00050
Prostate	NA	NA	NA
Testis	NA	NA	NA
Nerve - Tibial	NA	NA	NA
Skin - Sun Exposed (Lower leg)	NA	NA	NA
Heart - Left Ventricle	0.61527	0.35282	0.05147
Brain - Cerebellum	NA	NA	NA
Cells - Transformed fibroblasts	NA	NA	NA
Whole Blood	0.12594	0.96252	0.00050
Artery - Aorta	0.17141	0.00050	0.18341
Cells - EBV-transformed lymphocytes	NA	NA	NA
Pituitary	NA	NA	NA
Liver	NA	NA	NA
Kidney - Cortex	NA	NA	NA
Brain - Hippocampus	0.54664	0.38631	0.20990
Brain - Substantia nigra	0.42812	0.33283	0.52224
Brain - Anterior cingulate cortex (BA24)	NA	NA	NA
Brain - Frontal Cortex (BA9)	NA	NA	NA
Brain - Cerebellar Hemisphere	NA	NA	NA
Brain - Caudate (basal ganglia)	1.00000	0.29535	0.36232
Brain - Nucleus accumbens (basal ganglia)	NA	NA	NA
Brain - Putamen (basal ganglia)	NA	NA	NA
Brain - Hypothalamus	NA	NA	NA
Brain - Spinal cord (cervical c-1)	1.00000	0.24438	0.41779
Brain - Amygdala	NA	NA	NA
Fallopian Tube	NA	NA	NA
Bladder	NA	NA	NA
Cervix - Ectocervix	NA	NA	NA
Cervix - Endocervix	NA	NA	NA

Supplementary File 2 - Subtypes found per known attribute: Ivy GAP dataset

	RCC	tSNE_km	hClust	mclust
CT	2	2	2	3
CTpan	2	2	1	2
CTmvp	1	1	1	1
LE	1	1	1	1

Supplementary File 2 - Subtypes found per known attribute: Human tissue dataset

	RCC	tSNE_km	hclust
Artery - Aorta	8	2	3
Lung	8	2	1
Artery - Tibial	7	1	3
Esophagus - Mucosa	7	1	2
Whole Blood	6	3	4
Adipose - Visceral (Omentum)	6	2	1
Thyroid	6	1	2
Cells - Transformed fibroblasts	6	1	1
Colon - Transverse	5	3	3
Artery - Coronary	5	2	2
Adrenal Gland	5	2	2
Skin - Not Sun Exposed (Suprapubic)	5	2	1
Skin - Sun Exposed (Lower leg)	5	2	1
Breast - Mammary Tissue	4	3	4
Small Intestine - Terminal Ileum	4	2	2
Heart - Atrial Appendage	4	1	2
Esophagus - Muscularis	3	4	3
Esophagus - Gastroesophageal Junction	3	4	2
Muscle - Skeletal	3	3	1
Brain - Caudate (basal ganglia)	3	2	2
Nerve - Tibial	3	2	1
Pancreas	3	1	1
Cells - EBV-transformed lymphocytes	3	1	1
Stomach	2	4	2
Vagina	2	3	4
Brain - Hippocampus	2	3	2
Adipose - Subcutaneous	2	2	3
Colon - Sigmoid	2	2	2
Minor Salivary Gland	2	2	1
Brain - Anterior cingulate cortex (BA24)	2	2	1
Brain - Amygdala	2	2	1
Testis	2	2	1
Brain - Nucleus accumbens (basal ganglia)	2	2	1
Brain - Putamen (basal ganglia)	2	2	1
Brain - Substantia nigra	2	1	2
Heart - Left Ventricle	2	1	2
Liver	2	1	1
Brain - Cerebellum	2	1	1
Brain - Cerebellar Hemisphere	2	1	1
Pituitary	2	1	1
Brain - Hypothalamus	1	3	1
Brain - Frontal Cortex (BA9)	1	2	1
Brain - Spinal cord (cervical c-1)	1	1	2
Ovary	1	1	1
Uterus	1	1	1
Brain - Cortex	1	1	1
Spleen	1	1	1
Prostate	1	1	1
Kidney - Cortex	1	1	1
Bladder	0	0	0
Fallopian Tube	0	0	0
Cervix - Endocervix	0	0	0
Cervix - Ectocervix	0	0	0

Supplementary File 2 - Subtypes found per known attribute: TCGA pan-cancer dataset

	RCC	tSNE_km	hClust
PRAD	11	2	2
LGG	10	3	1
COAD	9	1	2
UCEC	8	2	4
HNSC	7	3	3
THCA	7	2	1
BRCA	6	4	6
BLCA	6	3	5
KIRP	6	3	2
LIHC	6	1	2
PCPG	6	1	1
KIRC	5	3	4
STAD	5	2	6
READ	5	1	1
ESCA	4	2	5
SKCM	4	2	4
SARC	4	2	2
LUSC	3	4	4
CESC	3	2	4
UCS	3	1	2
GBM	3	1	1
THYM	3	1	1
TGCT	2	2	2
OV	2	1	3
PAAD	2	1	3
LUAD	2	1	2
DLBC	1	1	2
KICH	1	1	2
ACC	1	1	1
CHOL	1	1	1
MESO	1	1	1
UVM	1	1	1

Supplementary File 2 - Subtypes found per known attribute: Biase dataset

	RCC	tSNE_km	SC3
2cell	1	2	1
4cell	1	2	1
zygote	1	1	1
ICM	0	0	0
TE	0	0	0

Supplementary File 2 - Subtypes found per known attribute: Pollen dataset

	RCC	tSNE_km	SC3
BJ	3	1	1
GW16	2	1	2
HL60	1	3	2
Kera	1	2	1
2338	1	1	1
2339	1	1	1
GW21.3	1	1	1
iPS	1	1	1
K562	1	1	1
NPC	1	1	1
GW21	0	1	0

Supplementary File 2 - Subtypes found per known attribute: Darmanis dataset

	RCC	tSNE_km	SC3
Neoplastic_BT_S2	9	2	2
ImmuneCell17	7	10	8
Endothelial17	3	2	1
Neoplastic_BT_S1	3	1	2
FetalQuiescent	3	1	1
Neoplastic_BT_S4	3	1	1
Neoplastic_BT_S6	2	1	1
Oligodendrocyte17	2	1	1
Astrocytes15	1	1	1
Astrocytes17	1	1	1
FetalReplicating	1	1	1
Hybrid	1	1	1
ImmuneCell15	1	1	1
Neuron15	1	1	1
Neuron17	1	1	1
Oligodendrocyte15	1	1	1
OPC17	1	1	1
Endothelial15	0	1	1
OPC15	0	1	1

Supplementary File 2 - Subtypes found per known attribute: Neftel dataset

	RCC	tSNE_km	SC3
Macrophage	8	3	9
MGH100_Malignant	8	3	1
BT771_Malignant	7	4	3
MGH121_Malignant	7	4	2
MGH104_Malignant	7	2	1
MGH66_Malignant	6	5	2
MGH105_Malignant	6	3	4
MGH124_Malignant	6	3	2
BT749_Malignant	6	2	2
MGH102_Malignant	6	2	2
BT1160_Malignant	6	1	1
MGH143CD45neg_Malignant	5	2	2
MGH136CD45neg_Malignant	5	2	1
MGH115_Malignant	5	1	2
MGH129CD45neg_Malignant	4	3	2
MGH122_Malignant	4	2	1
MGH101_Malignant	4	1	1
MGH110_Malignant	4	1	1
MGH125_Malignant	3	2	2
MGH106_Malignant	3	2	1
BT830_Malignant	3	1	1
BT920_Malignant	3	1	1
MGH113_Malignant	3	1	1
MGH151CD45neg_Malignant	2	2	1
MGH85_Malignant	2	2	1
Oligodendrocyte	2	1	1
BT786_Malignant	2	1	1
MGH152CD45neg_Malignant	2	1	1
T-cell	1	1	1
MGH128CD45neg_Malignant	1	1	1
BT1187_Malignant	0	0	0
